# Supplementary material for: Confining charge-transfer complex in a metal-organic framework for photocatalytic CO2 reduction in water
Source: Nat Commun. 2023 Jul 26;14:4508. doi: 10.1038/s41467-023-40117-z (PMC10371996; doi:10.1038/s41467-023-40117-z)
Supplement: Supplementary file 1 — Supplementary Information [file 41467_2023_40117_MOESM1_ESM.pdf]

Supplementary Information

Confining Charge-Transfer Complex in a metal-organic framework for Photocatalytic CO_2 Reduction in Water

Sanchita Karmakar[1], Soumitra Barman[1], Faruk Ahamed Rahimi[1], Darsi Rambabu[1], Sukhendu Nath[2] and Tapas Kumar Maji[1]

[1]Molecular Materials Laboratory, Chemistry and Physics of Material Unit (CPMU),
School of Advance Material (SAMat),
Jawaharlal Nehru Centre for Advanced Scientific Research, Jakkur, Bangalore-560064, India
[2]Ultrafast Spectroscopy Section, Radiation & Photochemistry Division, Bhabha Atomic Research Centre, Mumbai 400 085, India.
*Email: tmaji@jncasr.ac.in; Tel: 91-8022082826

Experimental Section:

Synthesis of Microporous MOF-808 [$Zr_6O_4(OH)_4(BTC)_2(HCOO)_6$]: Microporous MOF-808 was synthesized using a slightly modified reported procedure.[1,2] H_3BTC (2.1 g, 10 mmol) and $ZrOCl_2\cdot 8H_2O$ (9.7 g, 30 mmol) were dissolved in DMF/formic acid (300 mL/300 mL) and placed in a 1-L screw-capped glass jar, which was heated to 130 °C for two days. A white precipitate was collected by filtration and washed three times with 200 mL of fresh DMF. As-synthesized microporous MOF-808 was then immersed in 100 mL of anhydrous DMF for three days, during which time the DMF was replaced three times per day. The DMF-exchanged compound was filtrated off and immersed in 100 mL of water for three days, during which time the water was replaced three times per day. Water exchanged material was then immersed in 100 mL of anhydrous acetone for three days, during which time the acetone was replaced three times per day. The acetone-exchanged sample was then evacuated at 150 °C for 24 h to yield an activated sample (Yield: 4.5 g, 70 % based on Zr).

Synthesis of Microporous MOF-808-PBA: In a 15 ml glass vial, 5 ml 0.1 M DMF/MeOH (1:2) solution of pyrenebutyric acid (PBA) molecule was added to 0.100 g of activated microporous MOF-808. The reaction mixture was placed in a 60 ºC oven for 20 days. Notably, during this period, the PBA molecule solution was replaced after three days. After 20 days, a pale yellow colour MOF was collected by filtration and washed several times with methanol to remove the unreacted PBA molecule. The exchanged product dried in oven at 60 ºC under vacuum. ^{1}H-NMR spectra of digested MOF-808-PBA (400 MHz, D_2O, ppm) δ: 8.1 (s, BTC), 8.2 (s, HCOOH), peak area ratio (BTC: HCOOH) = 6.0:3.0.

Calculation for CH_4 Selectivity:

We have calculated the selectivity based on the previous literature reports using the following equation.[3,4]

$$\text{Selectivity}_{\text{CH}_4} = \frac{8 * n_{\text{CH}_4}}{\left(8 * n_{\text{CH}_4}\right) + \left(2 * n_{\text{H}_2}\right)} \text{ x } 100\ \%$$

Where we have accounted the number of electrons required to convert CO_2 to CH_4 as well as the yield (mmol) of the products in the CO_2 photoreduction process. The product selectivity for CO_2 reduction to CH_4 on an electronic basis (8 e^- for the formation of CH_4 and 2 e^- for H_2)

has been calculated using the following equation, where n represented the yield (mmol) of the products in the CO_2 photoreduction after 16 h.

$$\text{Selectivity}_{\mathrm{CH_4}} = \frac{8 * 7.3\, mmol\, g^{-1}}{(8 * 7.3\, mmol\, g^{-1}) + (2 * 0.3\, mmol\, g^{-1})} \text{ x } 100\ \%$$

= 99 %

Electrochemical Characterization:

The Mott-Schottky and impedance measurement were carried out with a CHI760E electrochemical workstation (CH Instruments, USA) *via* a conventional three-electrode system in a 0.2 M Na_2SO_4 aqueous solution.

Preparation of working electrode for Mott-Schottky and impedance measurement:

2.5 mg of compound **(**MOF-808 / MOF-808-PBA / MOF-808-PBA-MV**)** was dispersed in a solution of 250 μL water, 250 μL isopropyl alcohol (IPA) and 10 μL of Nafion to prepare a homogenous slurry. Subsequently, 12 μL of the slurry was coated on a glassy carbon electrode and dried at room temperature. The Ag/AgCl electrode was employed as the reference electrode, and the platinum plate was used as the counter electrode. A white LED lamp (LEICA KL1600 LED) was used during impedance measurement.

Preparation of working electrode for transient photocurrent:

2.5 mg of compound **(**MOF-808/ MOF-808-PBA/ MOF-808-PBA-MV**)** was dispersed in a solution of 250 μL water, 250 μL isopropyl alcohol (IPA) and 10 μL of Nafion to prepare a homogenous slurry. Subsequently, 300 μL of the slurry was coated on an ITO glass plate (1 cm x 1 cm) and dried at room temperature. The Ag/AgCl electrode was employed as the reference electrode, and the platinum plate was used as the counter electrode, respectively. The transient photocurrent responses were carried out under visible-light irradiation conditions (300 W Xenon arc lamp). The photocurrent experiment was performed in 0.2 M Na_2SO_4 solution. For the photocurrent measurement, we applied a potential of 0.35 V vs Ag/AgCl while performing chronoamperometry measurement. During photocurrent measurement, after applying 0.35 V of applied potential. photocurrent measurement was performed in presence of the sacrificial electron donor. To perform the experiment, we dispersed the catalyst along with BNAH and thereafter coated it over ITO.

In situ diffuse reflectance FT-IR (DRIFT) measurement:

In situ diffuse reflectance FT-IR measurement was carried out by FT-IR spectrometer (BRUKER Vertex 70V) with a designed reaction cell. The catalyst was spin-coated over a glass slide and placed in the center of the designed reaction cell. A high vacuum pump was used to pump out all the gases from the reaction cell. Then pure CO_2 (99.99%) gas and H_2O vapour were passed into the reactor for 5 minutes. At last, visible light was turned on, and the IR signal was in-situ collected through the MCT detector along with the reaction time. To perform the *in-situ* experiment in the presence of BNAH and TEA, we dispersed the catalyst along with BNAH and thereafter coated it over a glass surface. Before starting the experiment, TEA (10 μL) was added to the glass surface. After that, a similar procedure was followed, as mentioned above. *In situ* DRIFT study during $^{13}CO_2$ and CO reduction, we purged the reaction mixture with $^{13}CO_2$ and CO, respectively as feeding gas instead of CO_2,.

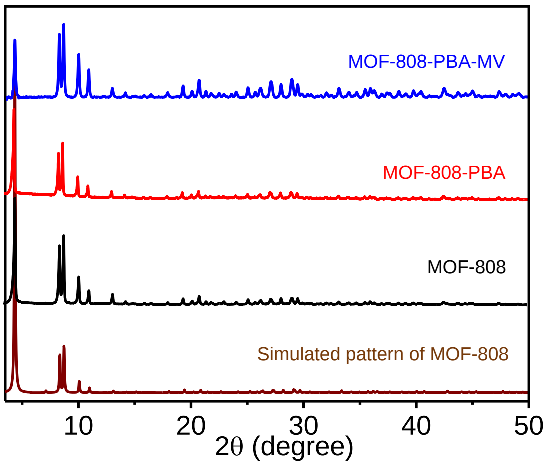

Supplementary Fig. 1 Powder XRD patterns. Comparison of PXRD pattern of as-synthesized MOF-808, MOF-808-PBA and MOF-808-PBA-MV with the simulated pattern of MOF-808.

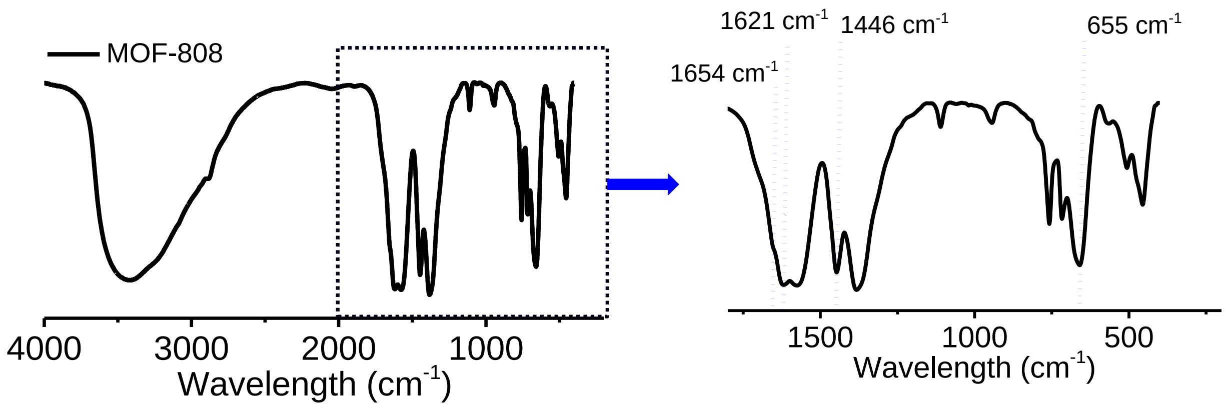

Supplementary Fig. 2 FTIR spectra of MOF-808. The FTIR spectra of MOF-808 exhibited peaks at 1621 and 1446 cm^{-1}, which were attributed to the aromatic ring of the BTC linker.[5] Notably, a peak at 1654 cm^{-1} was ascribed to the coordinated carboxylate group, thereby confirming the coordination of the BTC ligand onto the Zr^{IV} cluster of the framework. Importantly, a peak at 650 cm^{-1} was assigned to the asymmetric stretching frequency for the Zr-O bond.[5]

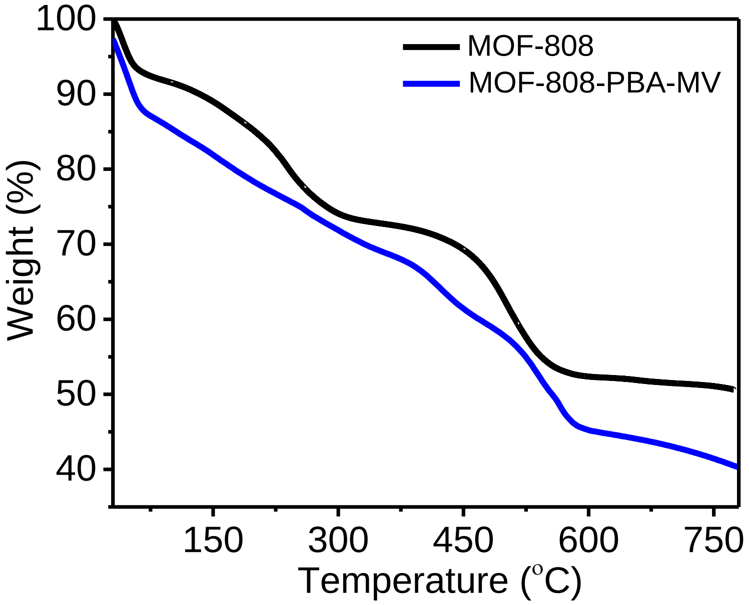

Supplementary Fig. 3 Thermogavimetric analysis. TGA plot of MOF-808 and MOF-808-PBA-MV under N_2 atmosphere at 5 °C min^{-1}. Thermogravimetric analysis of MOF-808 showed a typical two-step weight loss between the temperature range of 225-325 ˚C and 440-570 ˚C correspond to the loss of formate and 1,3,5-benzenetricarboxylate (BTC) linkers, respectively. Finally, MOF-808 exhibited weight loss of around 49.1 % up to 800 ˚C. Whereas, TGA analysis of MOF-808-PBA-MV showed a weight loss around 59.8 % up to 800 ˚C, which revealed an additional 10.7 % weight loss up to 800 °C as compared to pristine MOF-808, implementing the presence of PBA-MV moiety into the pore of MOF-808-PBA-MV.

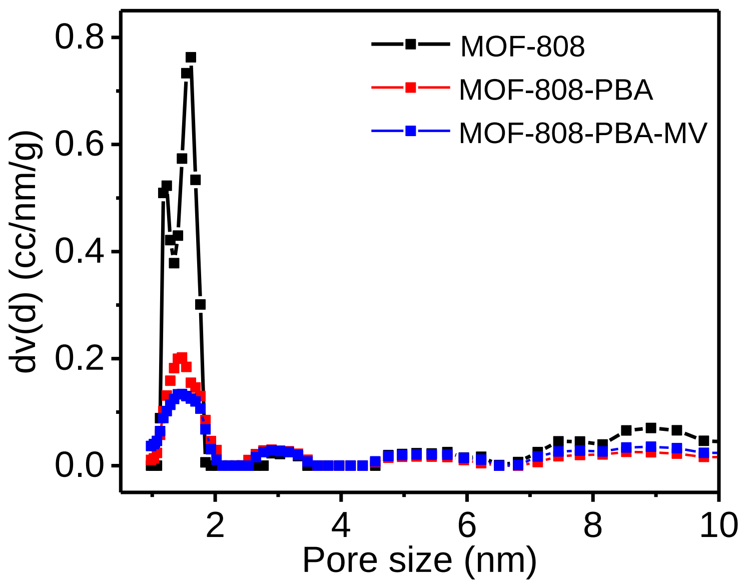

Supplementary Fig. 4 Pore size distribution. Pore size distribution for MOF-808, MOF-808-PBA, and MOF-808-PBA-MV.

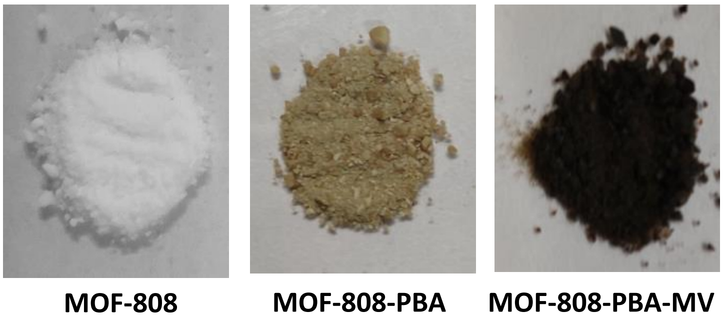

Supplementary Fig. 5 Photgraphic images. Photographic images of MOF-808, MOF-808-PBA, and MOF-808-PBA-MV. After the successful PBA molecule exchange process, the pristine MOF-808 changed from white to pale yellow. Further incorporating the MV into MOF-808-PBA, the colour turns dark brown from pale yellow, confirming the CT complex formation.

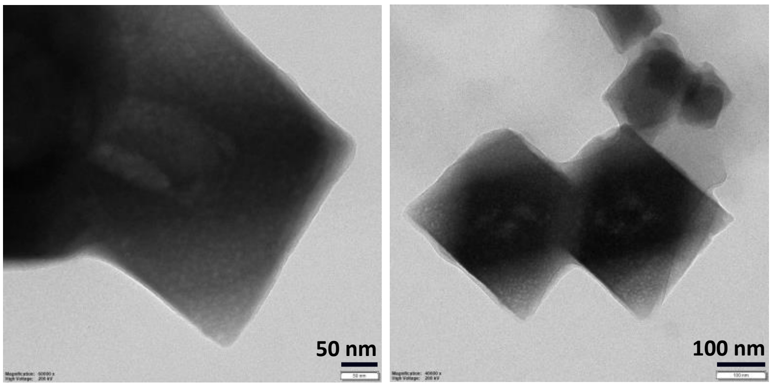

Supplementary Fig. 6 HRTEM images. HRTEM images of MOF-808-PBA.

NMR of **MOF-808** and **MOF-808-PBA**: The ^{1}H NMR spectra of digested MOF-808 in KOH/D_2O showed two prominent peaks at 8.2 and 8.1 ppm, attributed to the formate and benzene tricarboxylate acid (BTC) linkers, respectively.[6] The corresponding linker ratio of formate and BTC was calculated to be 5:6. In comparison, digested MOF-808-PBA in KOH/D_2O revealed that the formate peak integration diminished to one from five with the presence of additional peaks related to the PBA molecule. This result strongly indicated that four formate ligands of the Zr_6 cluster were substituted by the PBA molecule.

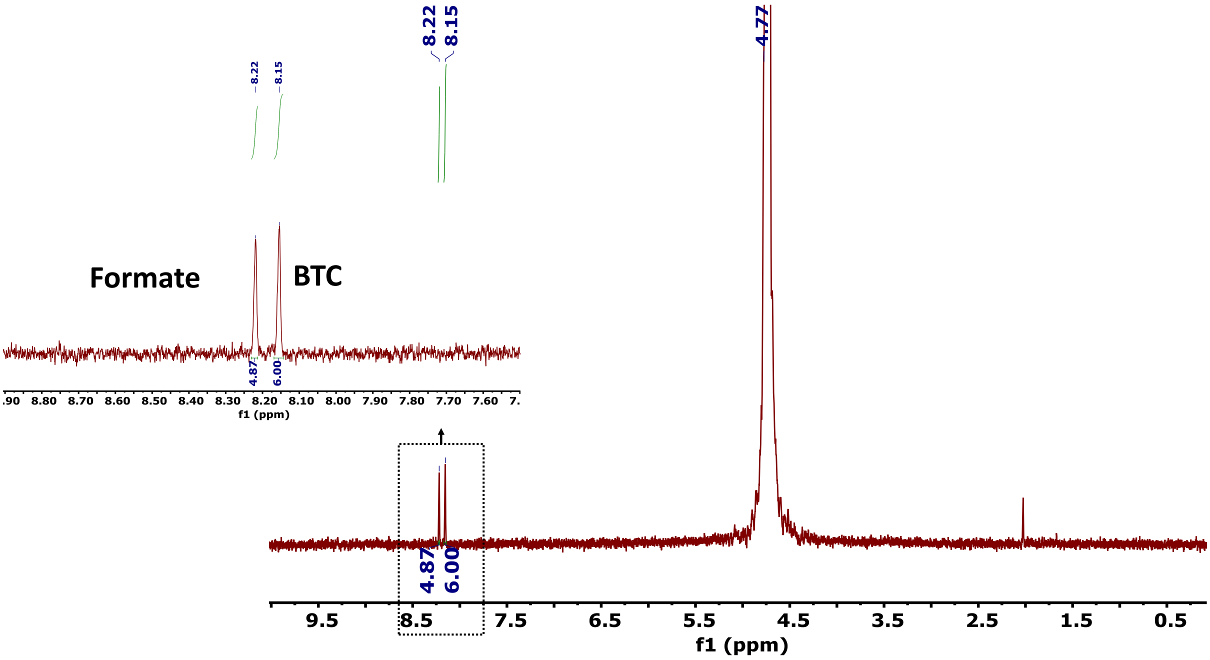

Supplementary Fig. 7 ^{1}H NMR spectra. ^{1}H NMR spectra for the digested MOF-808 in KOH/D_2O.

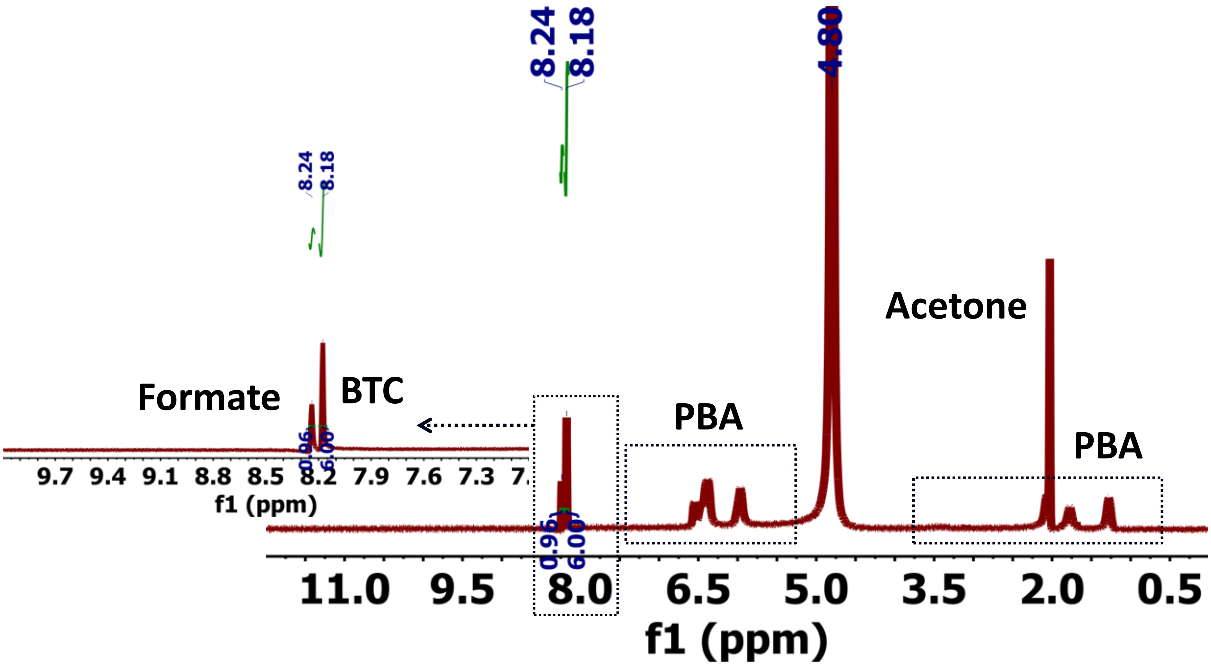

Supplementary Fig. 8 ^{1}H NMR spectra. ^{1}H NMR spectra for digested MOF-808-PBA in KOH/D_2O.

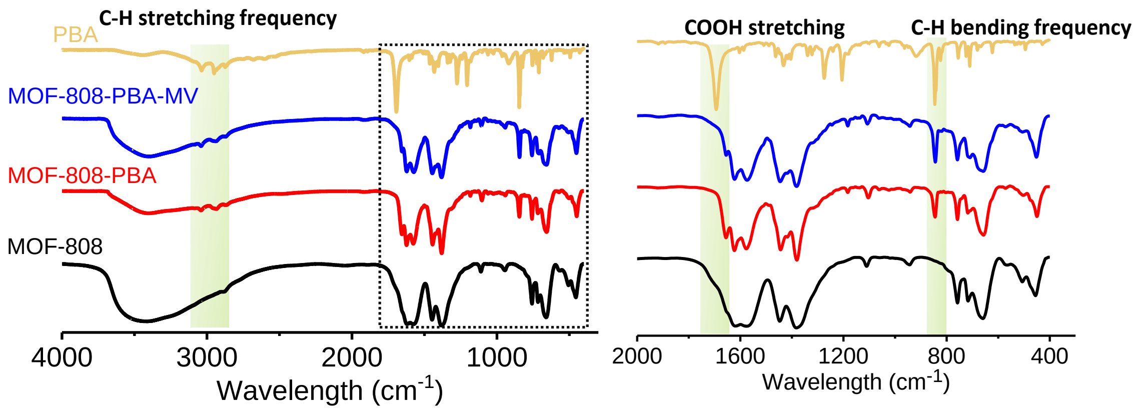

Supplementary Fig. 9 FT-IR spectra. FT-IR spectra for MOF-808, MOF-808-PBA, MOF-808-PBA-MV, and PBA. FT-IR spectra of MOF-808-PBA showed several new peaks at 2945, 1658 and 846 cm^{-1}, corresponding to C-H stretching, C=O stretching, and C-H bending frequency of PBA, respectively.[7]

Photophysical properties of PBA:

Usually, pyrene luminescence consists of both monomer and excimer luminescence. The monomeric emission band of PBA consists of five major vibronic bands with well-defined peaks at 376, 382, 387, 396 and 416 nm when excited at 320 nm. These correspond to the $\pi \rightarrow \pi^*$ transition of PBA. Concentration-dependent emission spectra were recorded to clarify the important role of concentration for the excimer formation. The result showed pure

monomeric emission with a concentration of 0.01 mM. Whereas, upon an increase in PBA concentration, the emission spectra showed the appearance of a very broad, red-shifted, new structureless band with a band maximum at 480 nm due to the formation of PBA excimer. Consequently, the emission spectrum of PBA in solid-state also exhibited a band at 480 nm.[8]

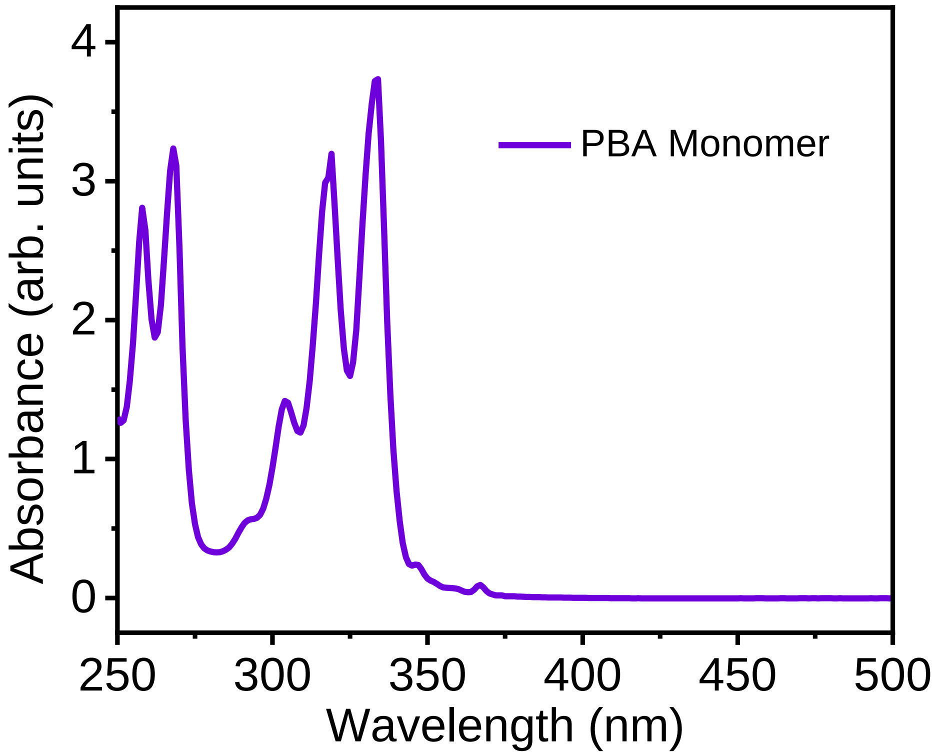

Supplementary Fig. 10 UV-vis spectrum. UV-vis spectrum of PBA (0.01 mM) monomer in MeOH**.**

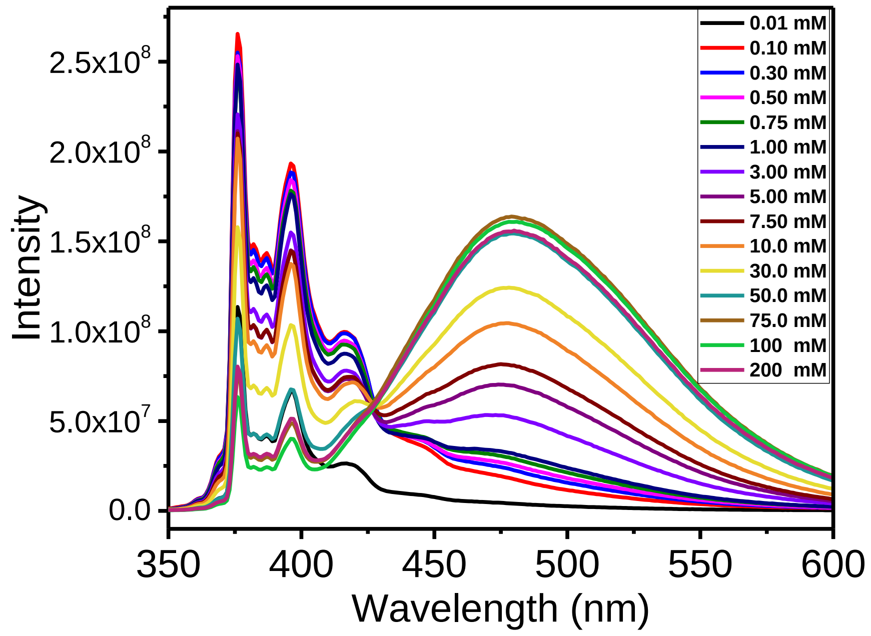

Supplementary Fig. 11 Emission spectra. Emission spectra of PBA in solution state (MeOH) with increasing PBA concentration (λ_{ex} = 320 nm).

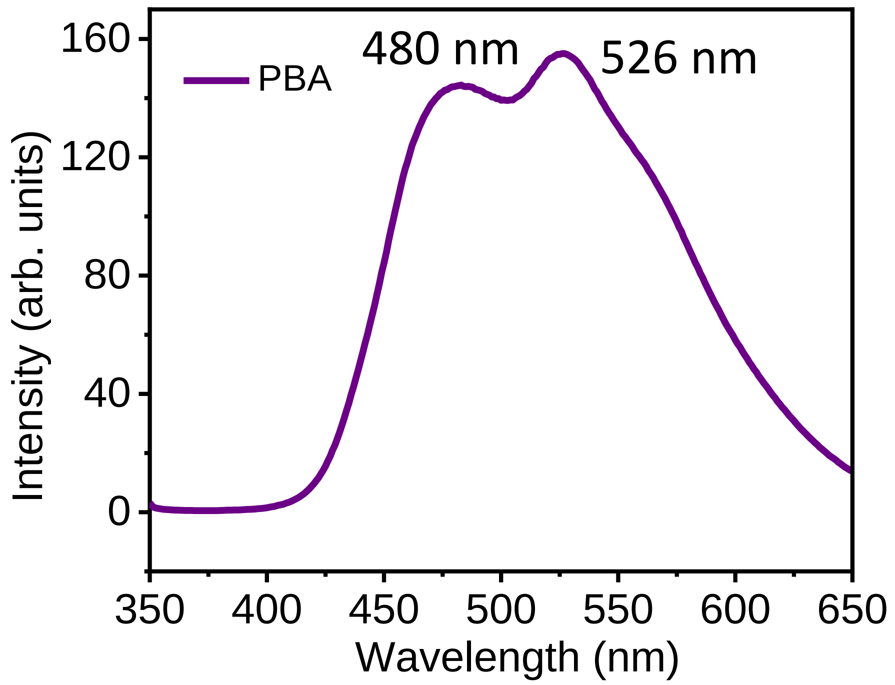

Supplementary Fig. 12 Emission spectra. Emission spectra of PBA in solid-state, showing excimer emission (λ_{ex} = 330 nm).

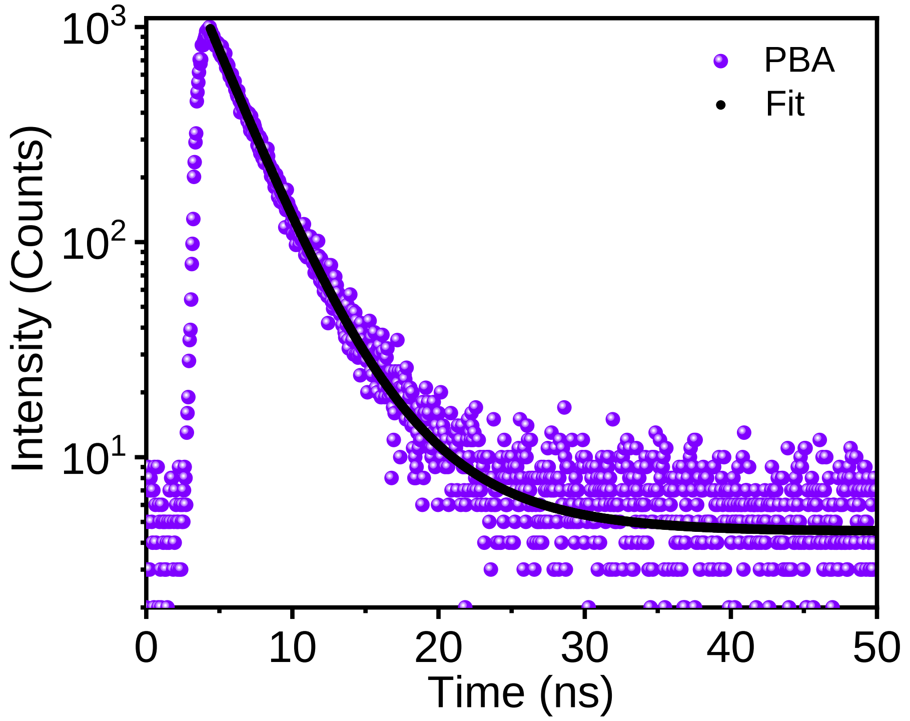

Supplementary Fig. 13 Lifetime experiment. Time-resolved luminescence decay of PBA monomer (0.01 mM) in methanol (λ_{ex} = 330 nm, λ_{col} = 400 nm).

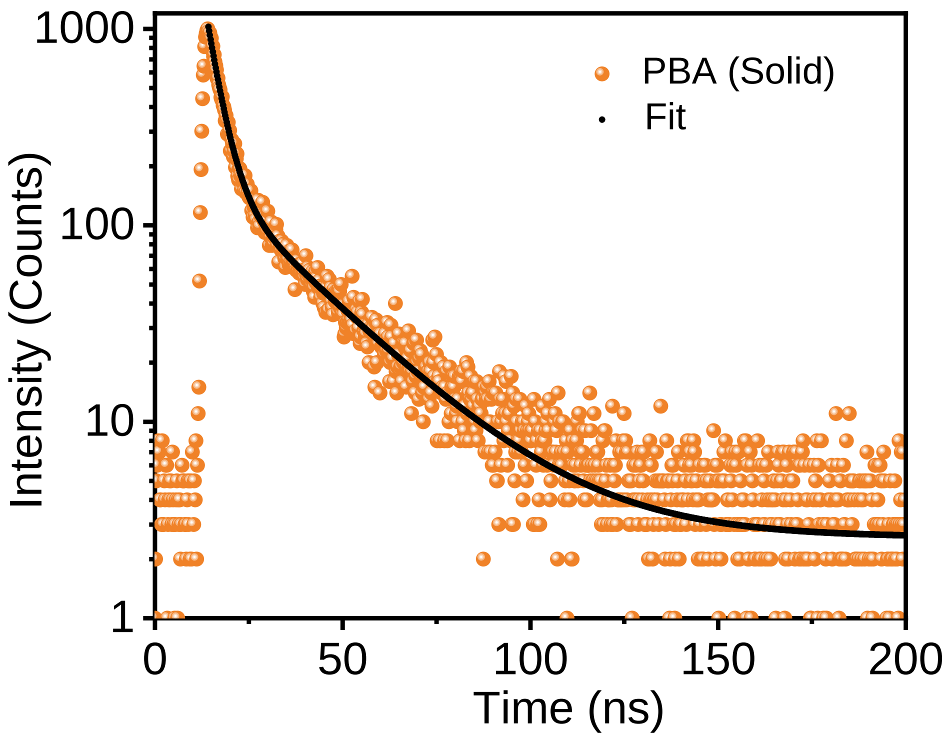

Supplementary Fig. 14 Lifetime experiment. Time-resolved luminescence decay of PBA excimer in solid-state (λ_{ex} = 330 nm, λ_{col} = 480 nm).

Supplementary Table 1: Summary of Time-resolved photoluminescence decay of MOF-808-PBA, MOF-808-PBA-MV, and PBA monomer.

**Compounds**	τ_1	τ_2	τ_3	τ_4	τ_{av}
**MOF-808-PBA**	2.29 ns (23.27 %)	23.36 ns (23.63 %)	49.8 ns (34.33 %)	0.321 ns (18.77 %)	23.2 ns
**MOF-808-PBA-MV**	0.55 ns (83.43 %)	4.03 (16.56 %)	-	-	1.12 ns
PBA monomer	2.535 ns (83.60 %)	5.593 ns (16.40 %)	-	-	3.01 ns
PBA solid	3.36 ns (44.55%)	23.47 ns (55.45 %)	-	-	14.51

Supplementary Table 2: Assignment of the CT band in the experimental solid-state absorption spectrum of **MOF-808-PBA-MV** with different transitions.

Absorption maximum (Experimental)	Theoretical	Transition	Type of transition	% Contribution
540 nm (450-800 nm)	596.01 nm	**HOMO→LUMO+1 (c= 0.49760)**	**CT**	**49.5%**
		HOMO→LUMO+2 (c= -0.31389)	CT	19.7%
		HOMO→LUMO+3 (c= 0.35477)	CT	25.2%

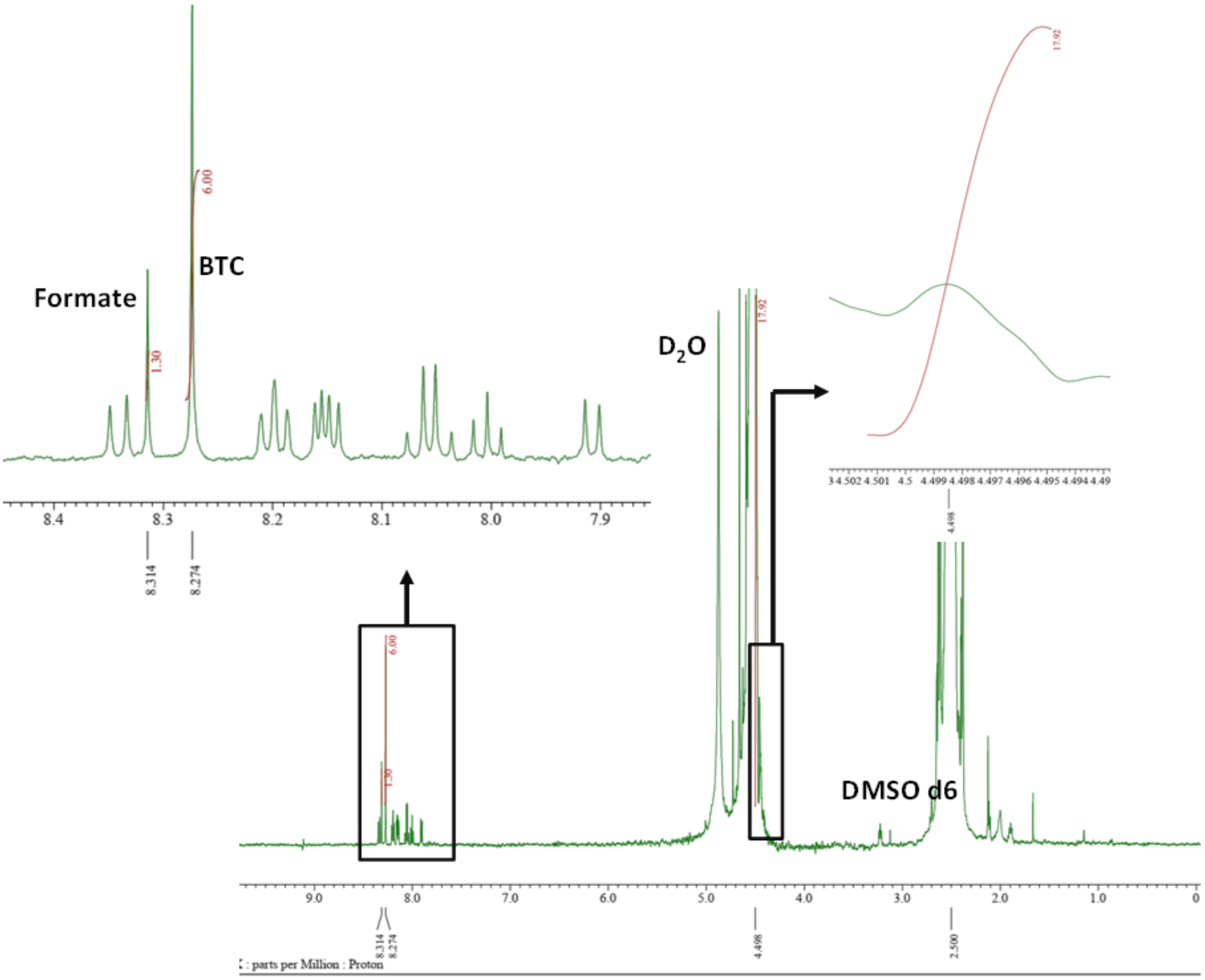

Supplementary Fig. 15 ^{1}H NMR spectra. ^{1}H NMR spectra for digested MOF-808-PBA-MV in KOH/D_2O/DMSO-d_6. Note: the addition of DMSO-d_6 was required to dissolve the PBA molecule.

Note: The ^{1}H NMR spectrum of digested MOF-808-PBA-MV in KOH/D_2O/DMSO-d_6 showed a prominent peak at 8.31 ppm attributed to the formate ligand, and integration of this peak suggested one formate unit remained in the framework after the post-synthetic modification. Therefore it is strongly suggested four formate units were exchanged with PBA molecule. It is worth mentioning that the addition of DMSO-d_6 was required to dissolve the PBA molecule. Additionally, there are several overlapping peaks in the region of 7.85 to 8.33 ppm, which correspond to the aromatic protons of PBA, MV^{2+} and BTC ligand. To further conclude the precise amount of MV^{2+} in the framework, we carefully looked to the aliphatic region for the CH_3 proton peak of MV^{2+}, which appeared at 4.49 ppm and integration of this particular peak suggested that 18 protons are present in the digested NMR. Hence, it implies the precise ratio of PBA and MV^{2+} is 4:3 in the MOF structure per formula unit.

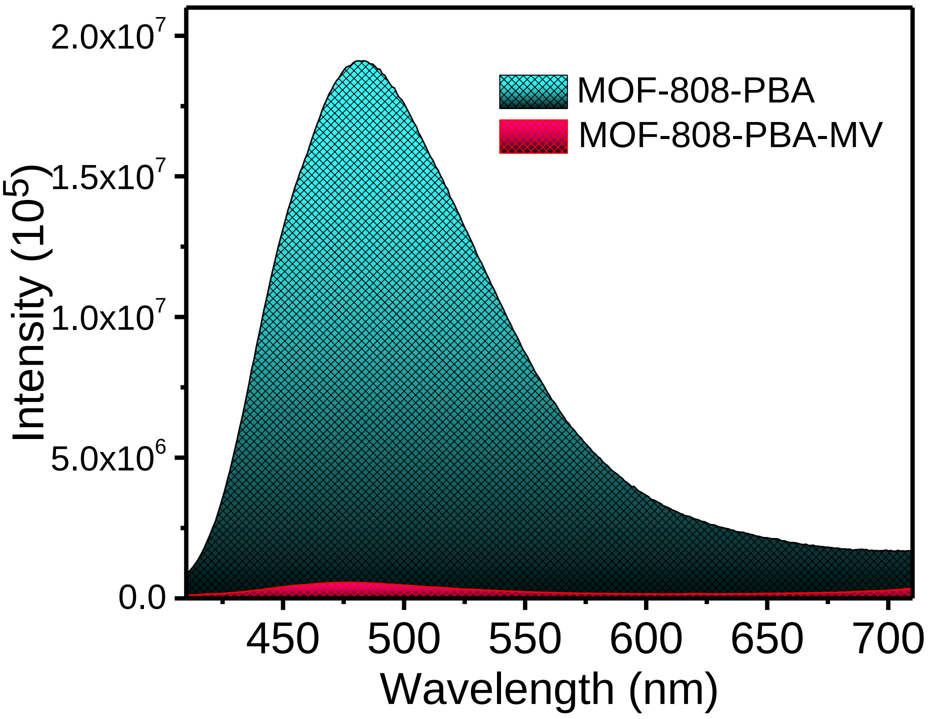

Supplementary Fig. 16 Photoluminescence spectra. Photoluminescence spectra of MOF-808-PBA and MOF-808-PBA-MV. (λex = 320 nm). [Note: $I_{MOF\text{-}808\text{-}PBA} = 2.195 * 10^9$; $I_{MOF\text{-}808\text{-}PBA\text{-}MV} = 6.942 * 10^7$ (I: integrated area under the curve)]

The extent of CT complex formation inside the confined pore was quantified from the integrated area of the PL spectra and found to be 96.84 % of PBA interacts with the MV resulting CT complex.

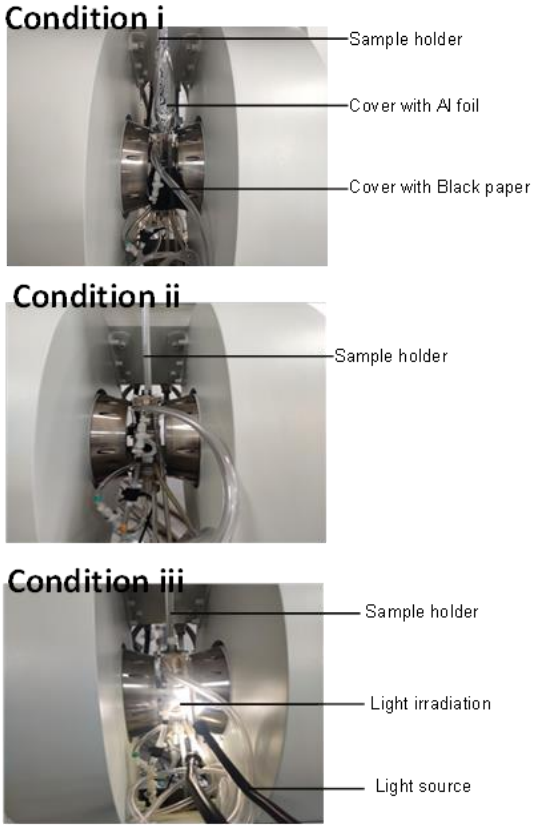

Supplementary Fig. 17 Images of the set-up utilized for EPR spectra. Condition i: Under completely dark conditions. Condition ii: Exposed under normal daylight. Condition iii: Irradiated with visible light source (Leica KL1600 LED).

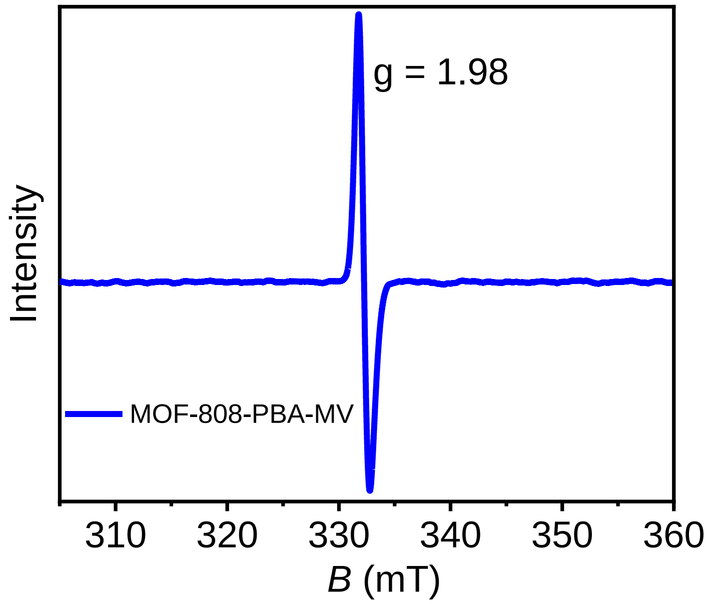

Supplementary Fig. 18 EPR spectrum. EPR spectra of MOF-808-PBA-MV under normal daylight (Condition ii).

Preparation of MOF-808-PBA-MV_d:

MOF-808-PBA-MV_d was prepared under completely dark conditions by wrapping the vial with aluminium foil by following a similar experimental method for preparing the MOF-808-PBA-MV, mentioned in the experimental section in the main text.

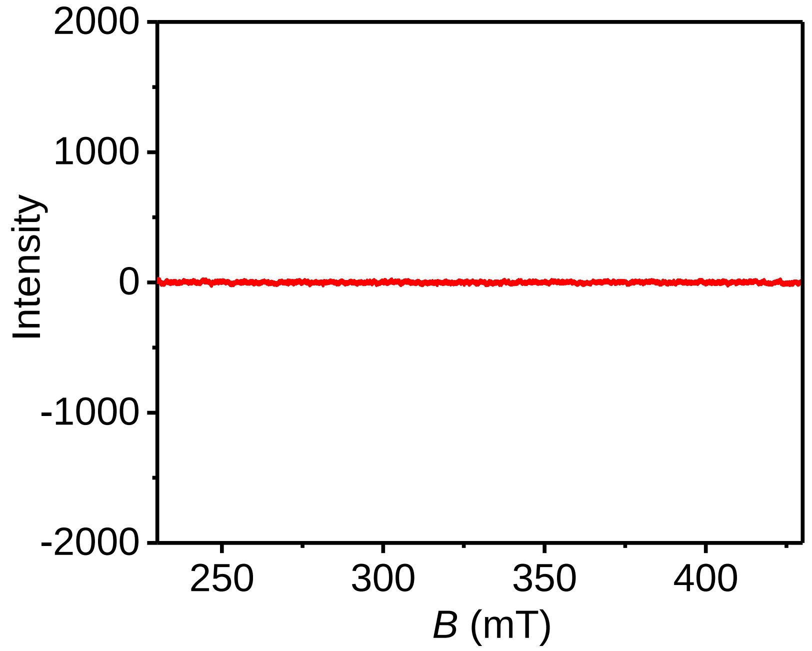

Supplementary Fig. 19 EPR spectrum. EPR spectrum of MOF-808-PBA-MV_d under dark condition (Condition i).

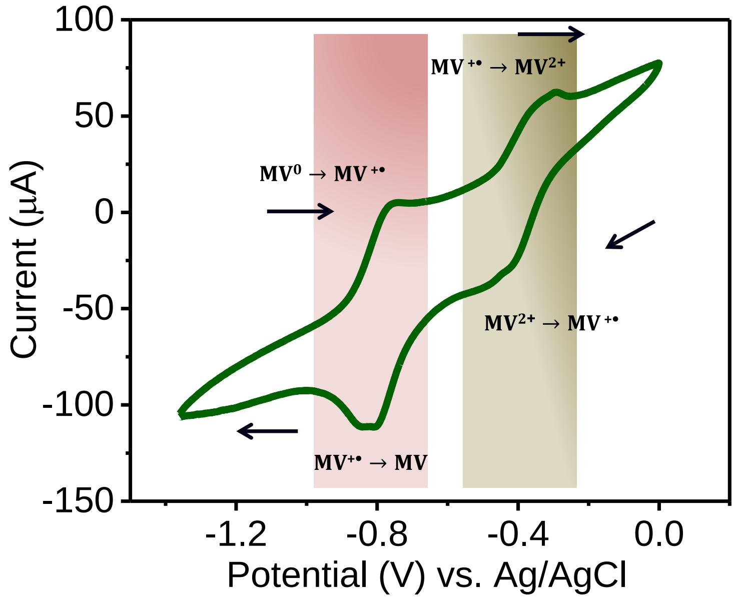

Supplementary Fig. 20 Cyclic voltammogram. Cyclic voltammogram of MV^{2+} (green) recorded in MeCN ($TBAPF_6$ 0.1 M).

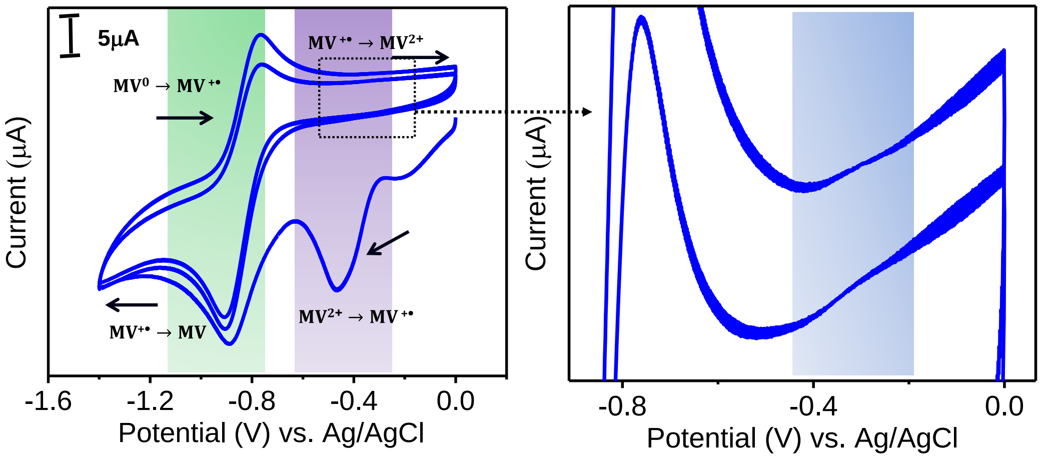

Supplementary Fig. 21 Cyclic voltammogram. Cyclic voltammogram of MOF-808-PBA-MV (green) recorded in MeCN ($TBAPF_6$ 0.1 M).

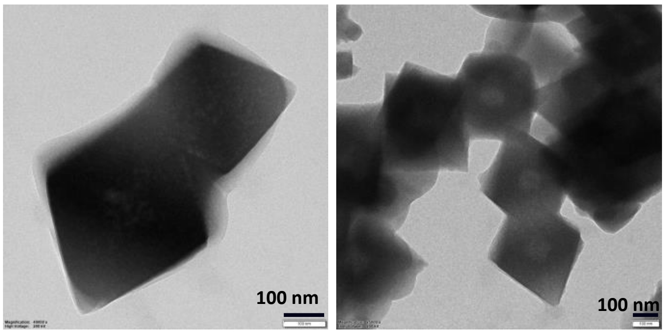

Supplementary Fig. 22 HRTEM images. HRTEM images of MOF-808-PBA-MV.

EDX and elemental mapping of MOF-808-PBA and MOF-808-PBA-MV: The EDX and elemental mapping of MOF-808-PBA-MV exhibited homogeneous distribution of nitrogen (2.7 wt%), including all the elements present in MOF-808-PBA, implying the integration of MV into the framework of MOF-808-PBA.

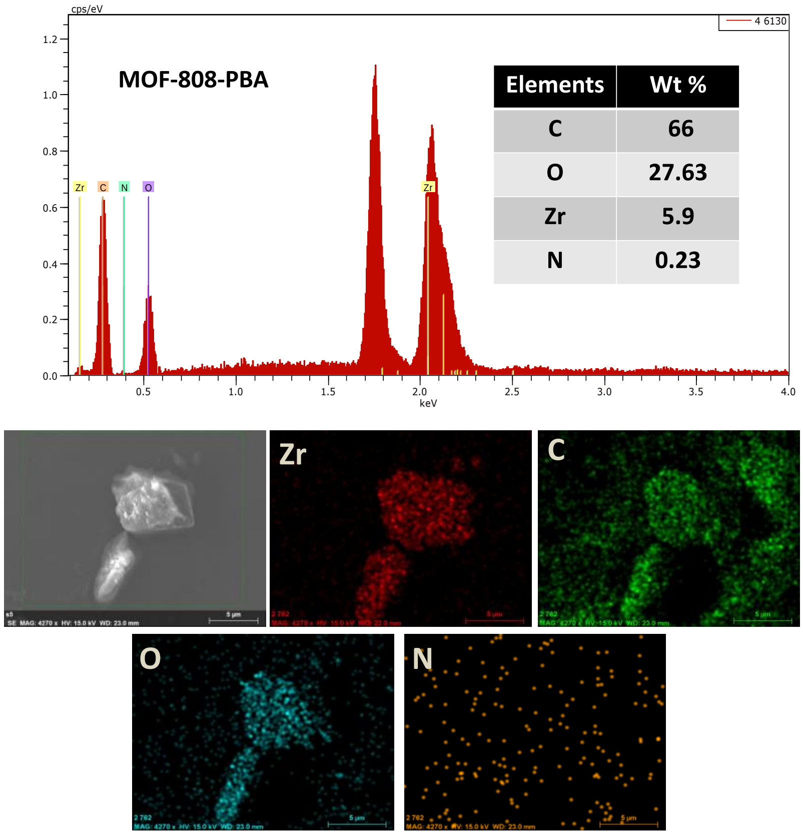

Supplementary Fig. 23 EDX and elemental mapping. EDX and elemental mapping of MOF-808-PBA.

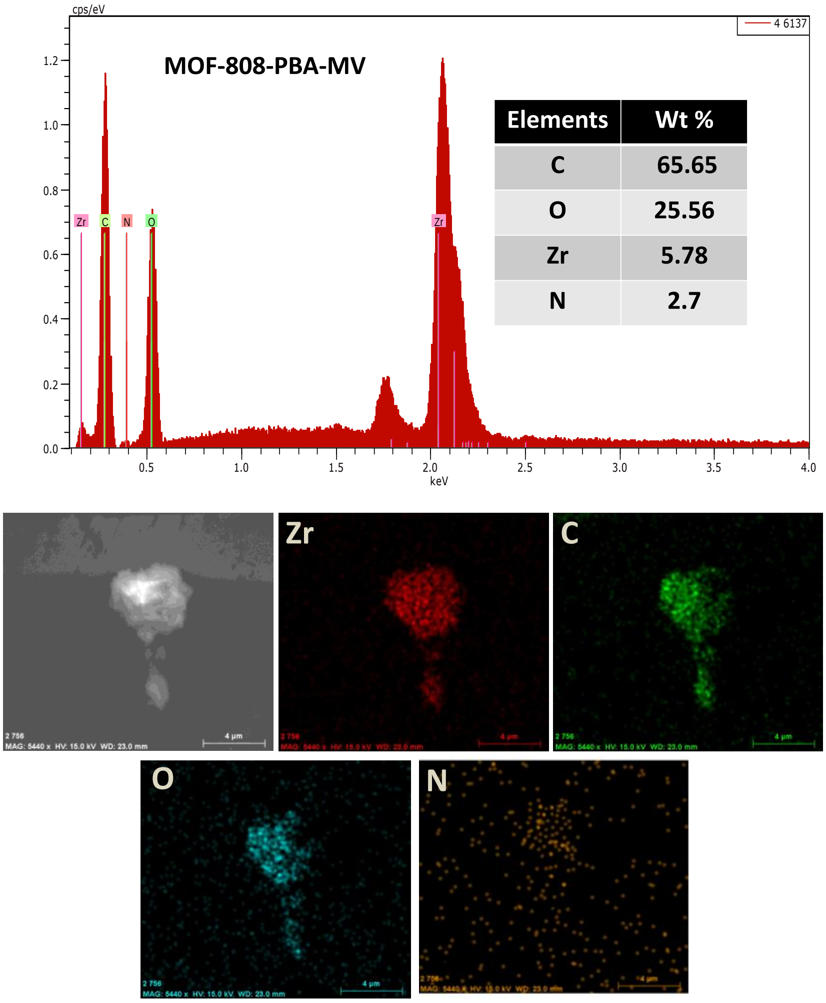

Elements	Wt %
C	65.65
O	25.56
Zr	5.78
N	2.7

Supplementary Fig. 24 EDX and elemental mapping. EDX and elemental mapping of MOF-808-PBA-MV.

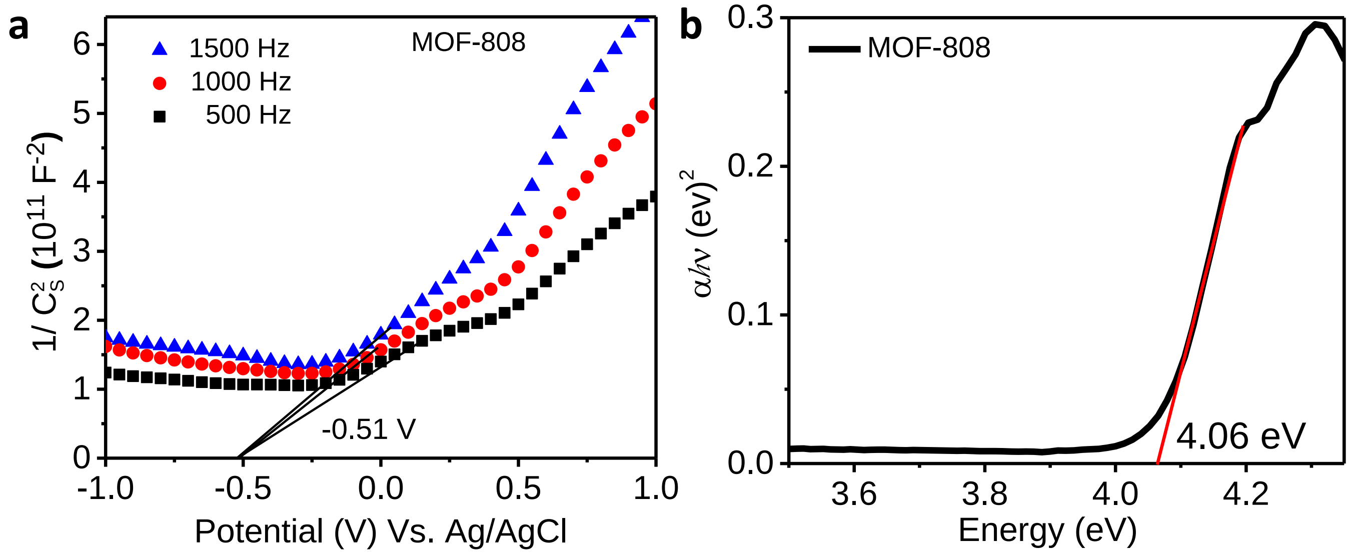

Supplementary Fig. 25 a Mott-Schottky and Tauc plot analysis of MOF-808. Mott-Schottky (MS) plot for MOF-808 in 0.2 M Na_2SO_4 aqueous solution. **b** Tauc plot-optical bandgap energy calculation for MOF-808.

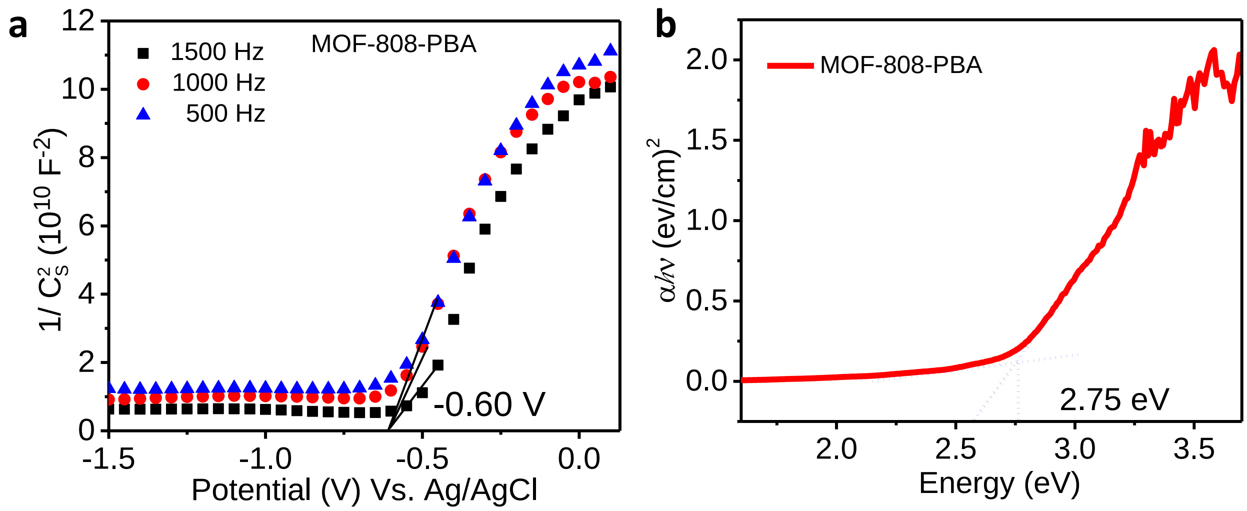

Supplementary Fig. 26 Mott-Schottky and Tauc plot analysis of MOF-808-PBA. **a** Mott-Schottky (MS) plot for MOF-808-PBA in 0.2 M Na_2SO_4 aqueous solution. **b** Tauc plot-optical bandgap energy calculation for MOF-808-PBA.

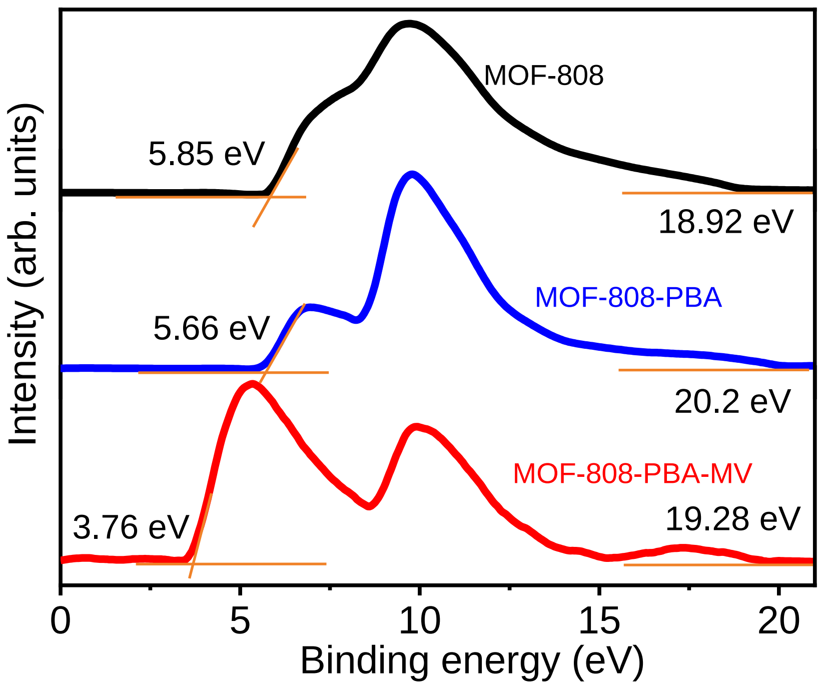

Supplementary Fig. 27 UPS spectra. UPS spectra in the cutoff and the onset energy regions of MOF-808, MOF-808-PBA, MOF-808-PBA-MV**.**

Supplementary Table 3 The energy levels within the MOF-808, MOF-808-PBA, MOF-808-PBA-MV obtained from the UPS and Mott-Schottky tests.

COMPOUNDS		MOF-808	MOF-808-PBA	MOF-808-PBA-MV
Bandgap		4.06 eV	2.75 eV	1.93 eV
UPS	HOMO	3.75 V	2.28 V	1.3 V
	LUMO	-0.31 V	-0.47 V	-0.63 V
M-S	HOMO	3.75 V	2.26 V	1.22 V
	LUMO	-0.31 V	-0.4 V	-0.67 V

Discussion on catalytic property of [MOF-808(PBA+MV)-Phy mix.]

To get a better insight into homogeneous and heterogeneous distribution of CT complex in MOF through post-synthetic modification, we performed additional experiments by preparing two compounds: one by physically mixing MOF-808 with PBA [MOF-808(PBA)-Phy mix.], and another by physically mixing MOF-808 with PBA and MV [MOF-808(PBA+MV)-Phy

mix.]. The physical mixtures were obtained by grinding the MOF with the respective molecule by maintaining the stoichiometry (as similar to post-synthetic modification) in the presence of a small quantity of DMF/MeOH. To evaluate the formation of the CT complex, the absorption spectrum of the MOF-808-PBA-MV was compared to the absorption spectra of MOF-808 (PBA+MV)-Phy mix (Supplementary Fig. 28). The results showed that MOF-808(PBA+MV)-Phy mix. exhibited an energetically high CT state, which was significantly different from the CT state observed in MOF-808-PBA-MV. Additionally, photocatalytic CO_2 reduction experiment was performed using the physical mixture in the presence of BNAH/TEA. The results showed minimal activity compared to MOF-808-PBA-MV (Supplementary Table 7). This poor activity can be attributed to the unstable CT complex in the solution state of MOF-808(PBA+MV)-Phy mix. which is clearly observed in UV-vis spectra (Supplementary Fig. 29). The absence of CT band implies that the CT complex formed by the physical mixture on the MOF-808 surface is not stable and disintegrate in the aqueous medium and thus could not act as light-harvesting agent to channelize the electron transfer the catalytic Zr-oxo cluster. However, MOF-808-PBA-MV formed a stable CT complex and optical activity due to the CT complex remaining stable as realized after the multiple catalytic cycles. In summary, the study suggests that the post-synthetic modification of MOF-808 with PBA-MV leads to a homogenous distribution of the CT complex, resulting in different electronic band structures compared to heterogeneous and irregular distribution of the CT complex obtained by the physical mixture. Such change in band structure in the stable post-modified MOF (MOF-808-PBA-MV) resulted in higher catalytic activity compared to the physical mixture of MOF-808, PBA, and MV.

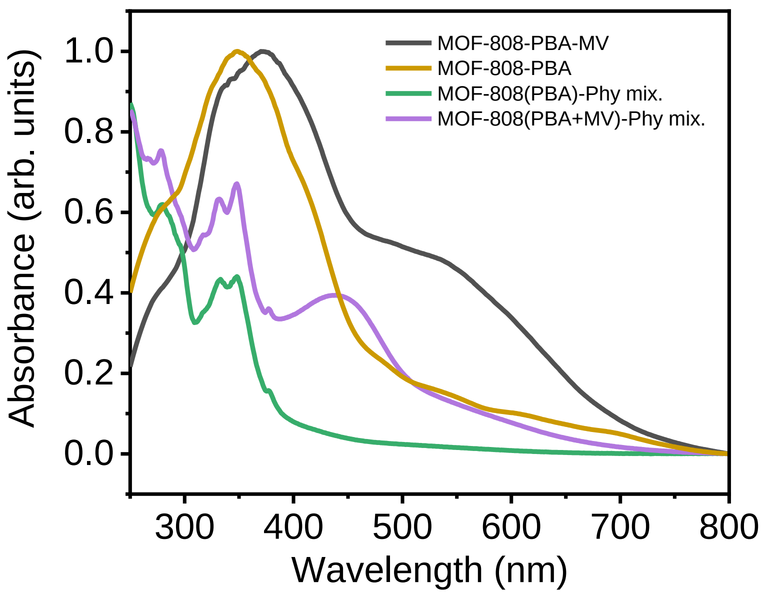

Supplementary Fig. 28 UV-vis spectra. UV-vis spectra of MOF-808-PBA-MV, MOF-808-PBA, MOF-808-(PBA)-Phy mix., MOF-808-(PBA+MV)-Phy mix.

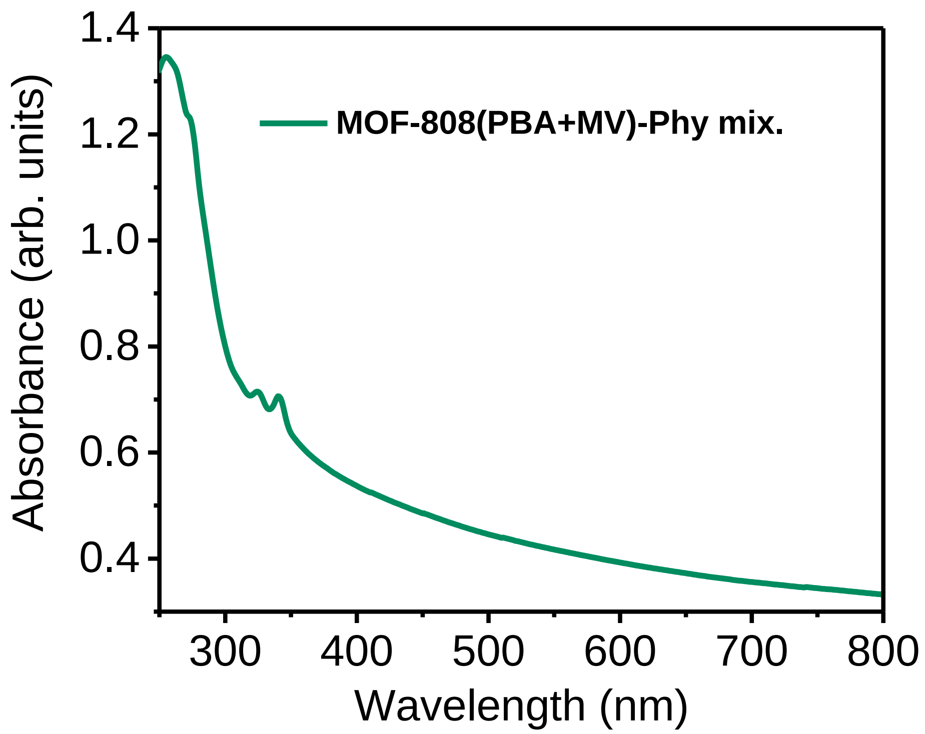

Supplementary Fig. 29 UV-vis spectra. UV-vis spectra of MOF-808(PBA+MV)-Phy mix.

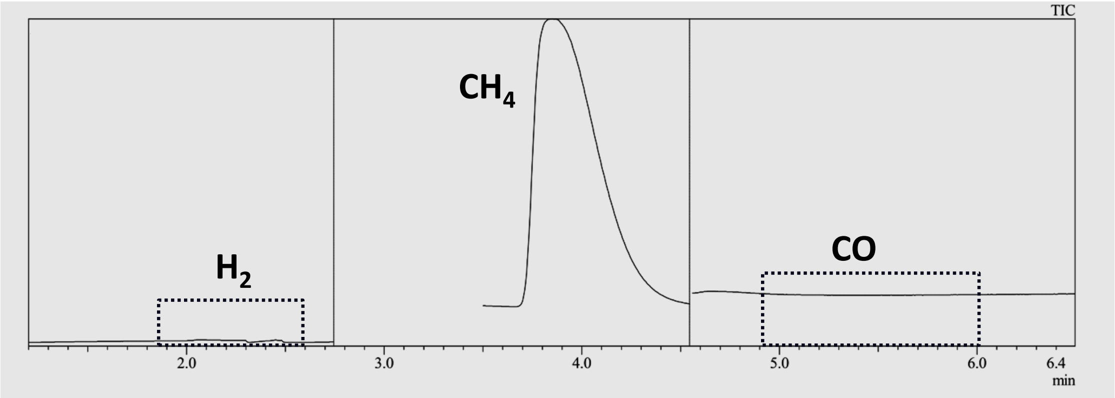

Supplementary Fig. 30 Gas chromatogram of CO_2RR. Gas chromatogram of CO_2 reduction of MOF-808-PBA-MV in aqueous medium using BNAH and TEA as sacrificial electron donor.

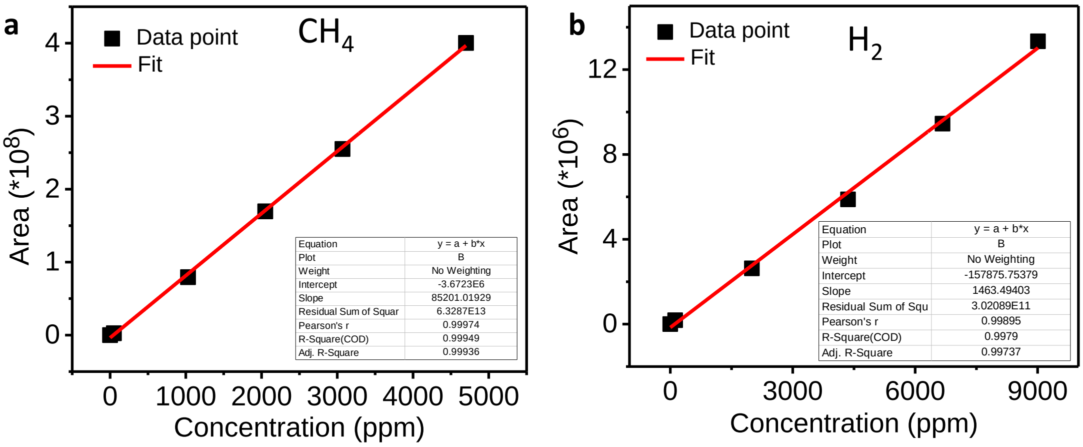

Supplementary Fig. 31 Calibration curve for GC-MS. **a** Calibration curve for GC-MS quantification of CH_4. **b** Calibration curve for GC-MS quantification of H_2.

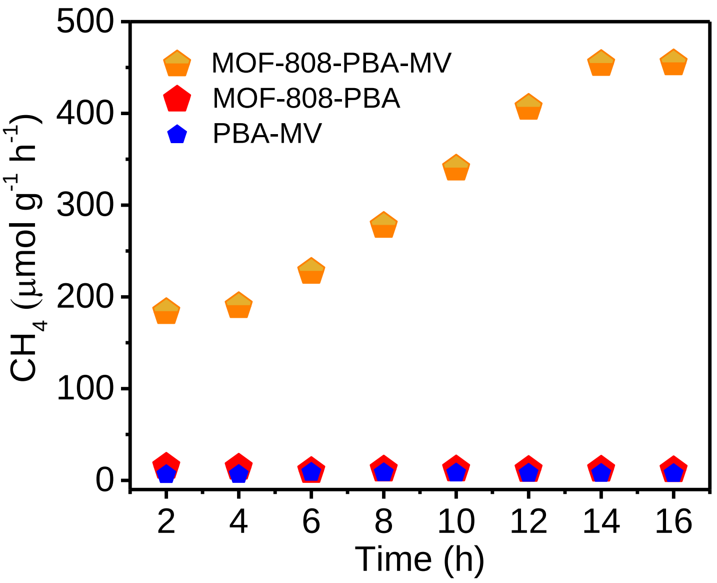

Supplementary Fig. 32 CH_4 production rate of CO_2RR. CH_4 production rate using MOF-808-PBA-MV (Yellow), MOF-808-PBA (Red) and homogeneous PBA-MV solution (blue).

Supplementary Table 4: Comparison of some state-of-art heterogeneous photocatalysts employed for photocatalytic CH_4 production.

Photocatalyst	Light Source	Sacrificial Agent	Solvent	Product	Product yield	Rate	Selectivity	Reference
**V_S-$CuInS_8$**	Visible light	-	spin-dropped on a quartz glass, 2 ml volume of DI water was injected	CH_4	208.8 μmol g^{-1} in 24 h	8.7 μmol $g^{-1}h^{-1}$	100 %	[9]
**MOF-808-Cu-Ni**	300 W Xe lamp > 420-760 nm	TEOA	MeCN/ H_2O	CH_4	1587 μmol g^{-1}	158.7 μmol $g^{-1}h^{-1}$	97.5 %	[10]
**Pt wt. % sensitized 0.35-BT-30**	simulated solar light	-	Water vapour	CH_4 along with C_2H_4	482.12 μmol g^{-1} in 24 h	80.35 μmol $g^{-1}h^{-1}$	-	[11]
**MOF-525-Co**	300 W Xe lamp (400 nm < λ < 800 nm	TEOA	MeCN	CH_4 and CO	0.42 μmol of CH_4 along with 2.42 μmol of CO (2 mg catalyst) i.e 210 μmol g^{-1} of CH_4 along with 1210 μmol g^{-1} of CO in 6 h	36.76 μmol $g^{-1}h^{-1}$ (CH_4) and 200.6 μmol $g^{-1}h^{-1}$	40.97 % (CH_4)	[12]
**$Cu_3(BTC)_2$@TiO_2**	UV irradiation	-	H_2O Vapor	CH_4	10.56 μmol g^{-1} in 4 h	2.64 μmol $g^{-1}h^{-1}$	100 %	[13]
**CPO-27-Mg/TiO_2**	365 nm UV light	-	H_2O Vapor	CH_4 and CO	23.5 μmol g^{-1} (CH_4)		36.49 % (CH_4)	[14]

					and 40.9 μmol g^{-1} (CO) in 10 h			
**(Au/Cu)/TiO_2**	Simulated solar light	-	Water	CH_4 and H_2	1400 μmol g^{-1} (CH_4) in 46 h		97 %	[15]
**Z-scheme CdS–WO_3**	300 W Xe lamp (λ > 420 nm)	-	Water	CH_4 along with CH_3OH		1.02 μmol $g^{-1}h^{-1}$	N.R	[16]
**Zn/PMOF**	UV-Vis	-	water	CH_4	34.8 μmol g^{-1}	8.7 μmol $g^{-1}h^{-1}$ in 4 h	NR	[17]
**Cu_2O@Cu@UiO-66-NH_2**	300 W Xe lamp (λ > 400 nm)	TEOA	glass fiber film	CH_4 and CO	41.5 μmol g^{-1}(CH_4) and 104.5 μmol g^{-1} (CO) in 5 h	8.3 μmol $g^{-1}h^{-1}$ (CH_4) and 20.9 μmol $g^{-1}h^{-1}$ (CO)	61.36 % (CH_4)	[18]
**5%A-PCN/CdSe−DETA**	300 W Xe lamp (λ > 420 nm)	-	Thin film	CH_4 and CO		2.19 μmol $g^{-1}h^{-1}$ (CH_4) and 25.87 μmol $g^{-1}h^{-1}$ (CO)	25.29 % (CH_4)	[19]
**MIL-101(Cr)−Ag**	300 W Xe lamp (400 nm < λ < 780 nm)	TEOA	glass fiber film	CH_4 and CO		427.5 μmol $g^{-1}h^{-1}$ (CH_4) and 808.2 μmol $g^{-1}h^{-1}$ (CO) in 18 h	62.90 %	[20]
**MOF-808-PBA-MV**	**>420 nm**	**BNAH/ TEA**	**Water**	**CH_4 and H_2**	**7.274 mmolg^{-1} (CH_4) and 0.369 mmolg^{-1} of H_2 in 16 h**	**460 μmol $g^{-1}h^{-1}$**	**99 % (CH_4)**	**This Work**

Supplementary Table 5: Comparison of the photocatalytic activities with the representative MOF based system.

Photocatalyst	Light Source	Sacrificial Agent	Solvent	Product	Product yield	Rate	Selectivity	Reference
**PCN-136 (Zr based MOF)**	300 W Xe lamp > 420-800 nm	TIPA	MeCN/ H_2O	HCOOH	10.52 μmol (20 mg) i.e. 526 μmol g^{-1} in 12 h	43.83 μmol $g^{-1}h^{-1}$	100 %	[21]
**MOF-808-Cu-Ni**	300 W Xe lamp > 420-760 nm	TEOA	MeCN/ H_2O	CH_4	1587 μmol g^{-1}	158.7 μmol $g^{-1}h^{-1}$	97.5 %	[10]
**MOF-525-Co**	300 W Xe lamp (400 nm < λ < 800 nm	TEOA	MeCN	CH_4 and CO	0.42 μmol of CH_4 along with 2.42 μmol of CO (2 mg catalyst) i.e 210 μmol g^{-1} of CH_4 along with 1210 μmol g^{-1} of CO in 6 h	36.76 μmol $g^{-1}h^{-1}$ (CH_4) and 200.6 μmol $g^{-1}h^{-1}$	40.97 % (CH_4)	[12]
**PCN-222**	300 W Xe lamp (400 nm < λ < 800 nm	TEOA	MeCN/ H_2O	HCOOH	30 μmol (50 mg) i.e. 600 μmol g^{-1} in 10 h	60 μmol g^{-1} h^{-1}	100 %	[22]
**Cu-Ru-MOF**	450 nm LED light	-	gas phase reaction purged with CO_2 and H_2	EtOH	N.R	9650 μmol·g^{-1} h^{-1}	>99 %	[23]
**Eu-Ru(phen)$_3$-MOF**	420 nm < λ < 800 nm	TEOA	MeCN	HCOOH	N.R	321.9 μmol h^{-1} $mmol_{MOF}^{-1}$	100 %	[24]
**NH_2-MIL-125(Ti)**	500 W Xe lamp (420 nm < λ < 800 nm	TEOA	MeCN	HCOOH	8.14 μmol (50 mg) i.e. 162.8 μmol g^{-1} in 10 h	16.28 μmol·g^{-1} h^{-1}	100 %	[25]
**MOF-253-Ru(bpy)$_2$Cl$_2$**	Xe lamp (420 nm < λ < 800 nm	TEOA	MeCN	HCOOH along with CO, H_2	0.67 μmol (HCOOH), 1.86 μmol (CO), 0.09 μmol (H_2) (5 mg) i.e. 134 μmol g^{-1} (HCOOH), 372 μmol g^{-1} (CO), 18 μmol g^{-1} (H_2) in 8 h	16.75μmol·g^{-1}·h^{-1} (HCOOH), 46.5 μmol·g^{-1}·h^{-1} (CO), 2.25 μmol·g^{-1}·h^{-1} (H_2) i	25.64% and 71.19 %	[26]

**$MAPbI_3$@PCN-221(Fe)**	300 W Xe (λ > 400 nm)	-	MeCN/H_2O (1: 0.012)	CO and CH_4	531 μmol g^{-1} (CO) and 1028 μmol g^{-1} (CH_4) in 25 h	21.24 μmol·g^{-1}·h^{-1} (CO) and 41.24 μmol·g^{-1}·h^{-1} (CH_4)	36.19 % (CO) 63.81 % (CH_4)	[27]
**Ti-Zr-MOF-525**	150 W Xe lamp (>400 nm)	-	Water vapour	CH_4 and CO	2.14 ± 0.09 μmol·g-1 (CH_4) 0.79 ± 0.05 μmol·g-1 (CO) in 6 h	1.52 μmol·g^{-1}·h^{-1} (CH_4) 0.50 μmol·g^{-1}·h^{-1} (CO)	87.13 % (CH_4)	[28]
**$CsPbBr_3$/ZIF-8**	AM 1.5G, 150 mW cm^{-2}	-	H_2O vapour	CO and CH_4	5.434 μmol g^{-1} (CH_4) 1.515 μmol g^{-1} CO) in 3 h	1.81 μmol $g^{-1}h^{-1}$ (CH_4) and 0.505 μmol $g^{-1}h^{-1}$ (CO)	93.48 % (CH_4)	[29]
**$CsPbBr_3$/ZIF-67**					10.537 μmol g^{-1} (CH_4) 2.301 μmol g^{-1} (CO) in 3 h	3.51 μmol $g^{-1}h^{-1}$ (CH_4) and 0.767μmol $g^{-1}h^{-1}$ (CO)	94.82 % (CH_4)	
**MOF-808-PBA-MV**	**>420 nm**	**BNAH, Triethyl amine (TEA)**	**Water**	**CH_4 and H_2**	**7.274 mmolg^{-1} (CH_4) and 0.369 mmolg^{-1} of H_2**	**460 μmol $g^{-1}h^{-1}$**	**99 % (CH_4)**	**This Work**

TIPA = Triisopropanolamine
TEOA = Triethanolamine

DMAc = Dimethylacetamide

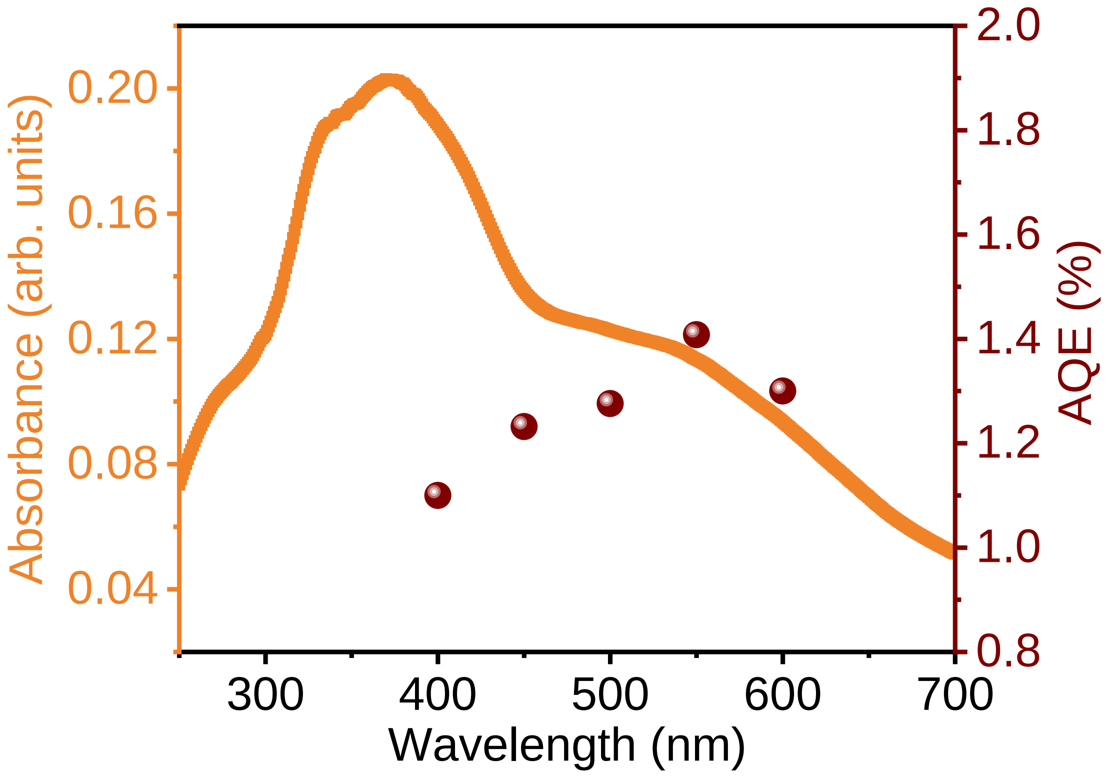

Supplementary Fig. 33 apparent quantum efficiency (AQE %) of MOF-808-PBA-MV in CO_2RR. UV-vis absorption spectra and apparent quantum efficiency (AQE %) plot for MOF-808-PBA-MV.

Apparent Quantum Efficiency Calculation:

Apparent quantum efficiency (AQE) is defined as the following equation: [1]

$$\text{AQE}\ \% = \frac{\text{Number of reacted electron}}{\text{Number of incident photon}} * 100\ \% \qquad \text{Eq. 1}$$

Eight electrons are required to convert CO_2 to CH_4.

$$AQE_{CH_4} = \frac{8 * \text{The number of evolved } \mathrm{CH_4} \text{ molecules}}{\text{The number of incident photon}} * 100 \quad \% \qquad \text{Eq. 2}$$

Two electrons are required to convert H_2O to H_2

$$AQE_{H_2} = \frac{2 * \text{The number of evolved } \mathrm{H_2} \text{ molecules}}{\text{The number of incident photon}} * 100 \quad \% \qquad \text{Eq. 3}$$

In the following, we described the AQE determination at 400, 450, 500, 550,600 nm over MOF-808-PBA-MV. The amounts of CH_4 produced were measured by applying different band-pass filters (400,450,500,550 and 600 nm) for 6 hours. The average intensity of irradiation with different band-pass filters was determined by a light meter (LI-COR Biosciences, LI-250A) at room temperature.

$$N = \frac{E\lambda}{hc} \qquad \text{Eq. 4}$$

e.g The number of incident photons (N) at 500 nm

$$N = \frac{5.6 * 10^{-3} * 1 * 3600 * 6 * 500 * 10^{-9}}{6.626 * 10^{-34} * 3 * 10^{8}} = 3.04256 * 10^{20}$$

$$AQE{-}CH_4(550) = \frac{8 * 6.023 * 10^{23} * 1.115 * 10^{-6}}{3.04256 * 10^{20}} * 100\,\% = 1.2\,\%$$

$$AQE{-}H_2(550) = \frac{2 * 6.023 * 10^{23} * 0.7735 * 10^{-6}}{3.04256 * 10^{20}} * 100\,\% = 0.208\,\%$$

Supplementary Table 6: Apparent quantum efficiency (AQE) % in different monochromatic light.

Wavelength (nm)	Power (mW cm^{-2})	Number of incident photon (N)	The amount of CH_4 evolved (μmol)	The amount of H_2 evolved (μmol)	Apparent quantum efficiency (AQE) %
**400 nm**	3.5	$1.5212 * 10^{20}$	0.236	0.441	1.1
**450 nm**	3.54	$1.731*10^{20}$	0.294	0.592	1.232
**500 nm**	5.6	$3.04256*10^{20}$	0.3725	0.3940	1.276
**550 nm**	7.5	$4.48234*10^{20}$	1.115	0.7735	1.408
**600 nm**	8.1	$5.28101*10^{20}$	0.8768	1.75	1.3

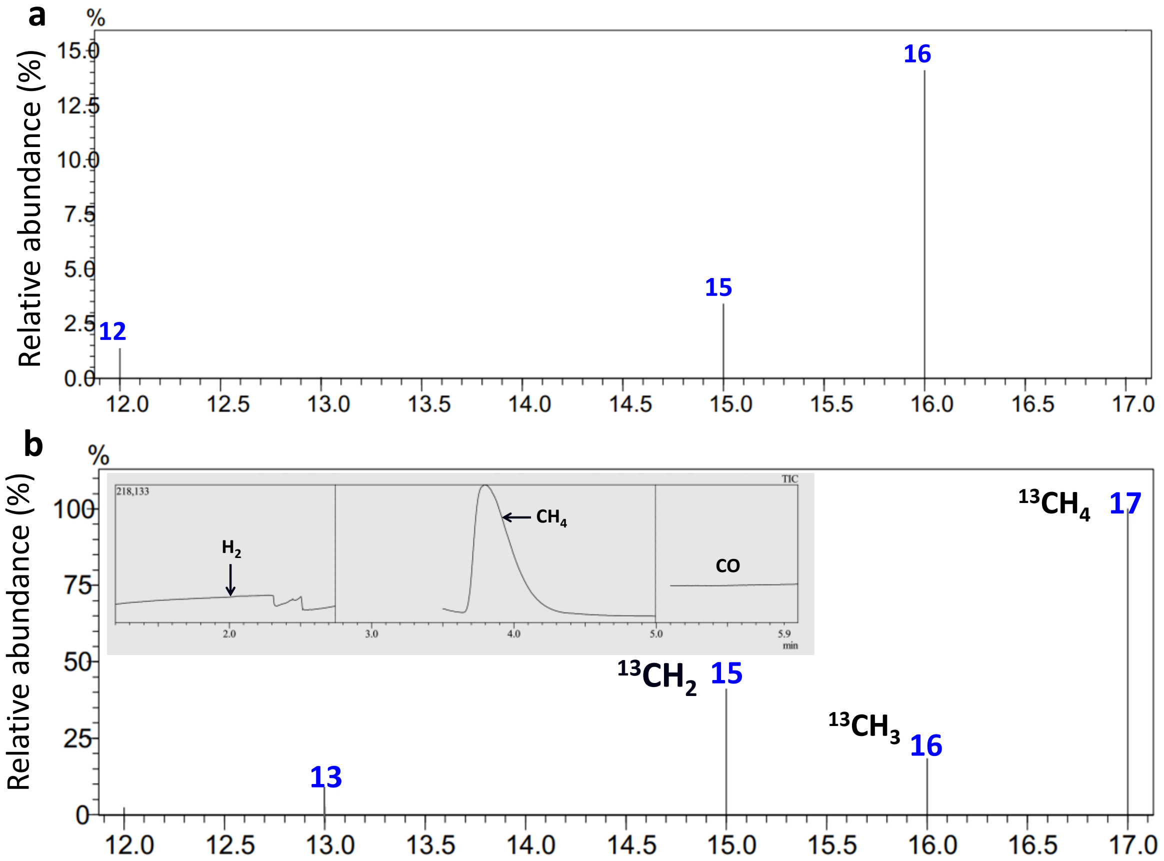

Supplementary Fig. 34 Origine of the CO_2 reduction product by using $^{12}CO_2$ and $^{13}CO_2$. **a** $^{12}CO_2$ mass-spectrum of produced $^{12}CH_4$ (m/z = 16) via isotope $^{12}CO_2$ reduction under visible light over MOF-808-PBA-MV. **b** $^{13}CO_2$ mass-spectrum of produced $^{13}CH_4$ (m/z = 16) via isotope $^{13}CO_2$ reduction under visible light over MOF-808-PBA-MV. Inset shows a typical chromatogram of $^{13}CH_4$ peak.

Control experiment of photocatalytic CO_2 reduction: PBA grafted MOF-808-PBA exhibited 180 μmol g^{-1} of CH_4 along with 76 μmol g^{-1} of H_2 in 16 h, and the production amount did not increase significantly with irradiation time, demonstrating the unsteady electron relay process from PBA to the catalytic centre, reflecting the sluggish kinetics towards CH_4 production (Rate: 12 μmolg^{-1} h^{-1}). Afterwards, by directly mixing PBA (0.5 mM) and MV (0.5 mM) in a homogeneous reaction condition, 120 μmol g^{-1} of CH_4 and 50 μmol g^{-1} of H_2 were produced, also demonstrating the important role of electron flow in a confined nanospace.

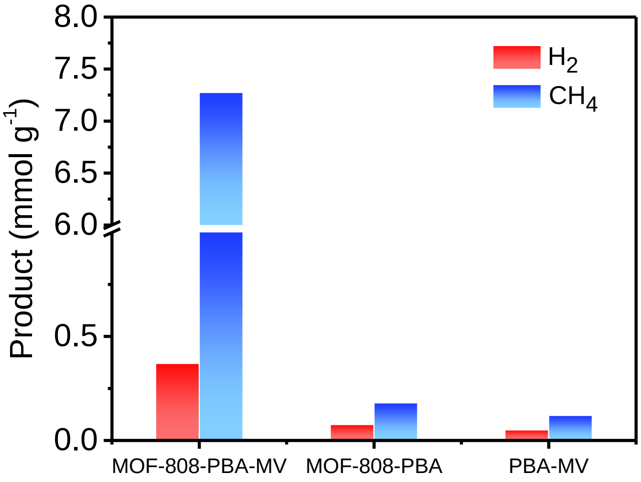

Supplementary Fig. 35 Comparison study of photocatalytic CO_2 reduction. Comparison of photocatalytic product generation by using MOF-808-PBA-MV along with MOF-808-PBA and homogeneous PBA-MV solution in water medium using BNAH and TEA as a sacrificial electron donor.

Supplementary Table 7: Comparision studies of photocatalytic CO_2 reduction.

Catalyst	Reaction condition	Sacrificial Agent	Products		
			**H_2 (μmol g^{-1})**	**CO (μmol g^{-1})**	**CH_4 (μmol g^{-1})**
MOF-808-PBA-MV	300 W Xe lamp,>400 nm CO_2 saturated water medium	BNAH, Triethylamine (TEA)	369.3	-	7274
MOF-808-PBA-MV	300 W Xe lamp,>400 nm CO_2 saturated water medium	-	67	54	460
MOF-808-PBA-MV	300 W Xe lamp,>400 nm CO_2 saturated water medium	BNAH	265	230	3567
MOF-808-PBA-MV	300 W Xe lamp,>400 nm CO_2 saturated water medium	Triethylamine (TEA)	291	456	1200
MOF-808-PBA	300 W Xe lamp,>400 nm CO_2 saturated water medium	BNAH, Triethylamine (TEA)	76	-	180
PBA-MV (Homogeneous)	300 W Xe lamp,>400 nm CO_2 saturated water medium	BNAH, Triethylamine (TEA)	50	-	120
**MOF-808**	300 W Xe lamp,>400 nm	BNAH, Triethylamine (TEA)	90	-	102

	CO_2 saturated water medium				
MOF-808(PBA+MV)-Phy mix.	300 W Xe lamp,>400 nm CO_2 saturated water medium	BNAH, Triethylamine (TEA)	104	45	56
Without catalyst	300 W Xe lamp,>400 nm CO_2 saturated water medium	BNAH, Triethylamine (TEA)	274	97	40
**MOF-808-PBA-MV**	300 W Xe lamp,>400 nm Ar saturated water medium	BNAH, Triethylamine (TEA)	601	0	0
Without catalyst	300 W Xe lamp,>400 nm CO_2 saturated water medium	Without sacrificial agent	0	0	0

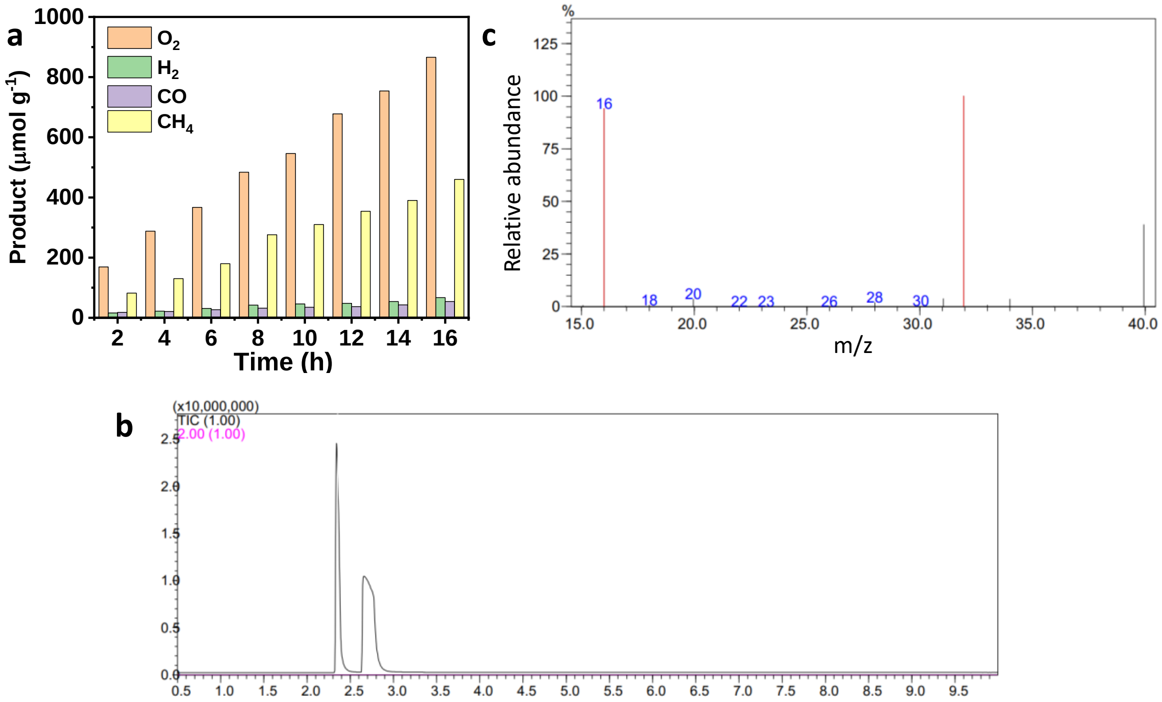

Supplementary Fig. 36 a Oxidative product during CO2RR. O_2 production (oxidation product from water oxidation) over MOF-808-PBA-MV as a function of time of irradiation in water. b Gas chromatogram of O_2 production over MOF-808-PBA-MV in water. c Mass spectrum of produced O_2 under visible light over MOF-808-PBA-MV.

The role of TEA and BNAH in the reaction process:

The catalytic reaction was performed in optimized reaction conditions using BNAH and TEA as a sacrificial agent. After photo irradiation of the reaction mixture, we have detected the mass fragments from those reaction mixtures using Liquid chromatography-mass spectroscopy (LC-MS). The peak at 426 corresponds to the formation of the BNA_2 molecule into the reaction medium after photoirradiation. It is also to be noted that after photoirradiation in the presence of both BNAH and TEA, adduct [BNAH-TEA] formation was occurred corresponds to an m/z value of 317, which was detected in LC-MS as well. A smaller amount of TEA was also found in the reaction medium attributed with m/z value of 102.

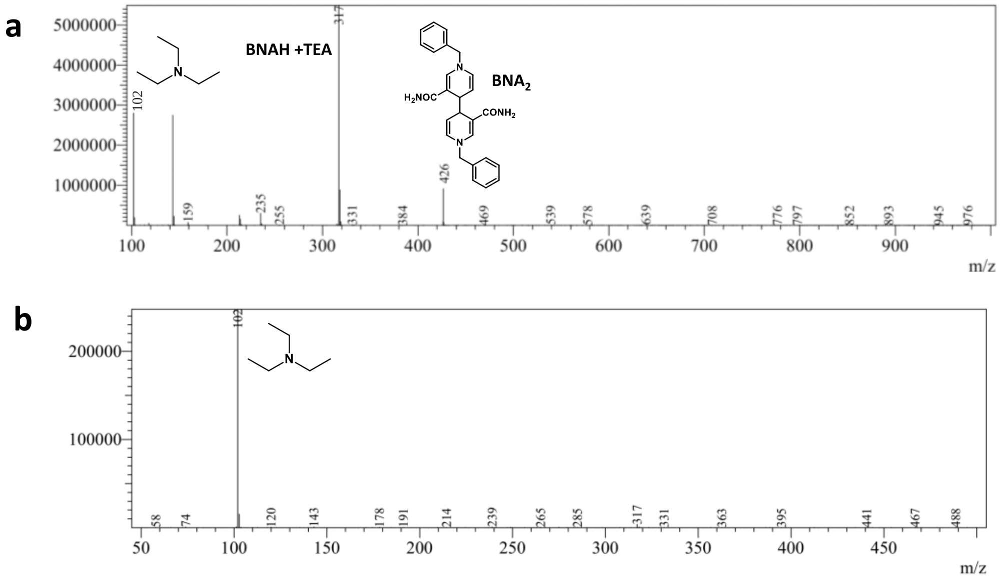

Supplementary Fig. 37 Liquid chromatography-mass spectrometry study to understand the role of sacrificial electron donor. **a** Liquid chromatography-mass spectrometry (LC-MS) of the liquid phase from the reaction system (Catalyst+ BNAH+TEA) after visible light irradiation for 16 h. **b** Liquid chromatography-mass spectrometry (LC-MS) of the liquid phase from the reaction system (Catalyst+TEA) after visible-light irradiation for 16 h.

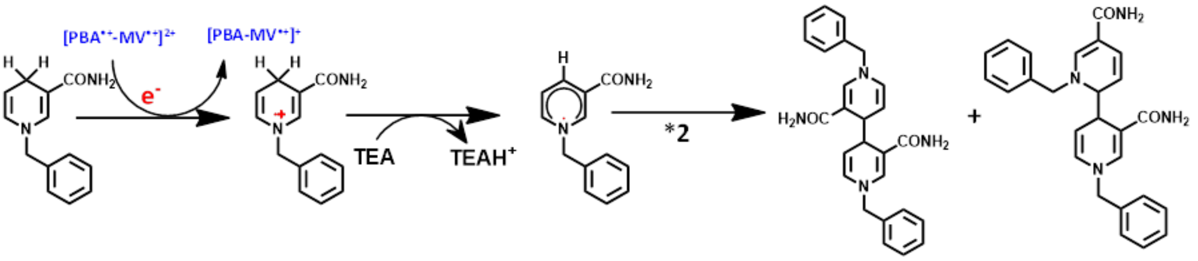

Supplementary Fig. 38 The role of BNAH. Oxidation and dimerization Processes of BNAH.

Comparison study for meso and microporous MOF-808:

Microporous MOF-808 was synthesised following the procedure mentioned above. Phase purity and porosity were established using PXRD and nitrogen adsorption measurements, respectively. The PXRD pattern of the as-synthesise microporous MOF-808 is well matched with the simulated pattern calculated from the single crystal structure of MOF-808 (Supplementary Fig. 39). N_2 adsorption of the microporous MOF showed the nearly identical porosity (surface area 2030 m^2g^{-1}) with the reported literature (Supplementary Fig. 40). Substantial experiments were carried out to understand the benefits of mesoporous MOF-808 compared to microporous MOF to construct the CT complex inside the MOF pore. Following the same reaction condition for the PBA ligand exchange process with the microporous MOF-808, three formate ligands were exchanged with PBA molecules after 20 days (Supplementary Fig. 41-43). This implies the mesopores of the defect engineered MOF makes the diffusion kinetics of PBA ligand faster compared to microporous analogue. Moreover, after adding MV in microporous MOF-808-PBA, the extent of CT complex formation was significantly less, confirmed by UV-vis spectra as well as visually confirmed by the light brown colour of the material (Supplementary Fig. 44).

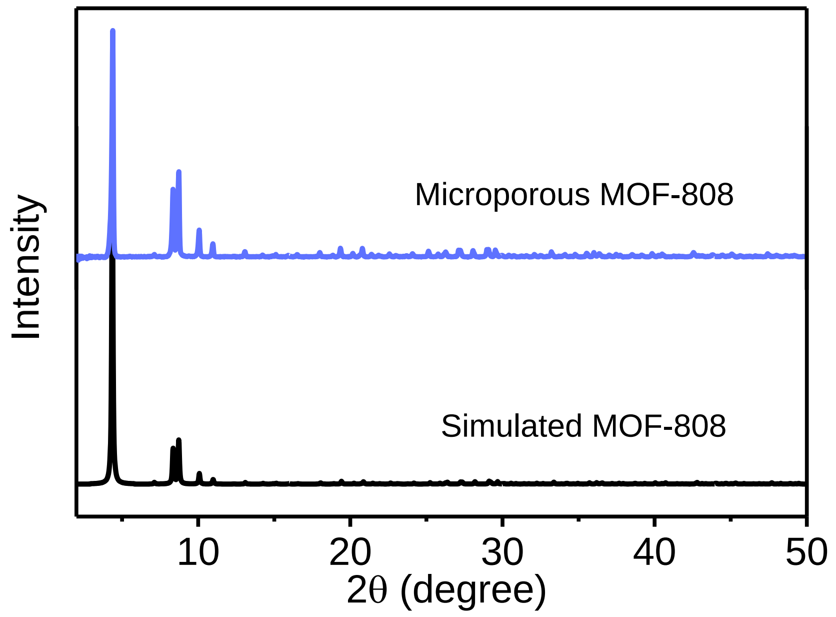

Supplementary Fig. 39 Powder XPS pattern. Comparison of PXRD pattern of as-synthesized microporous MOF-808, with the simulated pattern of MOF-808.

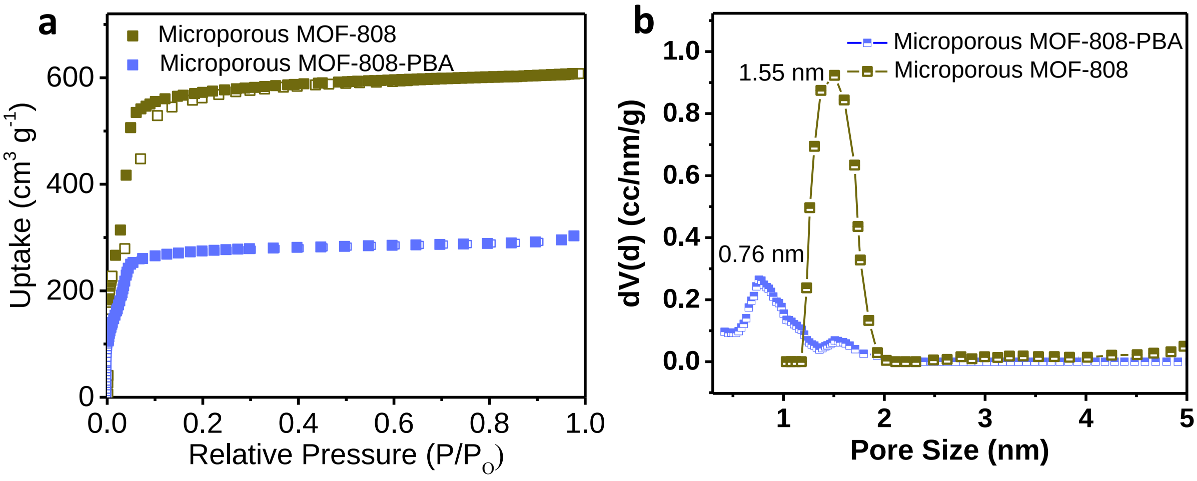

Supplementary Fig. 40 N_2 adsorption isotherm. a N_2 adsorption isotherm of microporous MOF-808, MOF-808-PBA. **b** Pore size distribution for MOF-808, MOF-808-PBA.

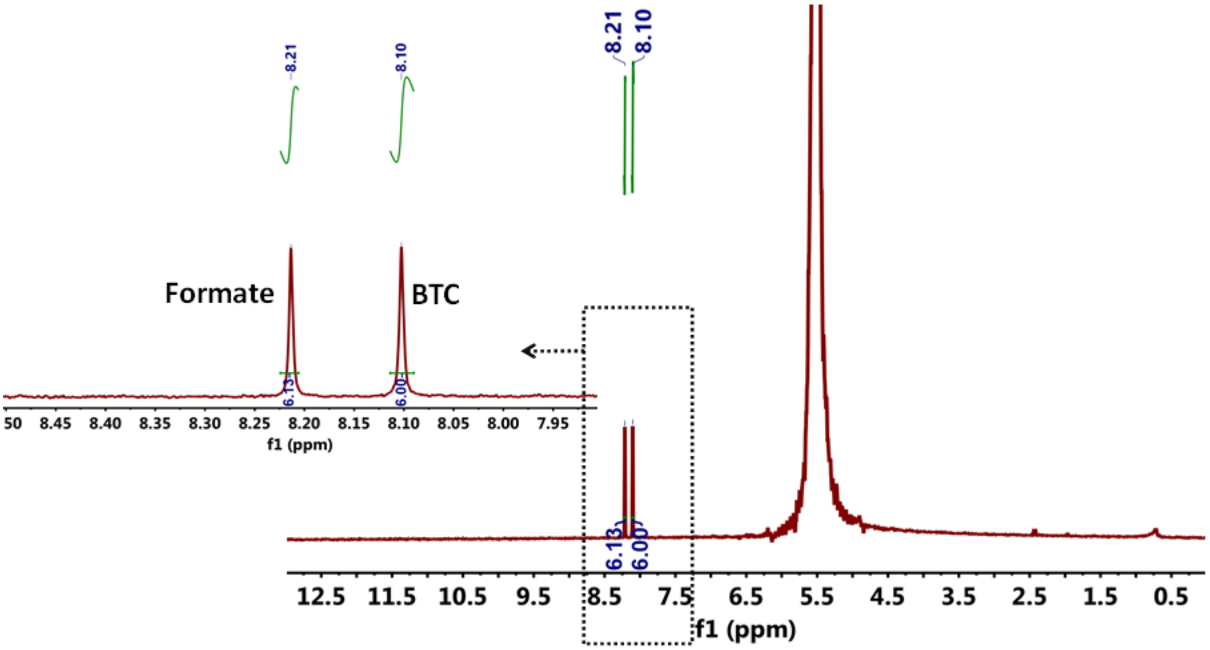

Supplementary Fig. 41 ^{1}H NMR spectra. ^{1}H NMR spectra for alkaline digested microporous MOF-808 in KOH/D_2O solution**.**

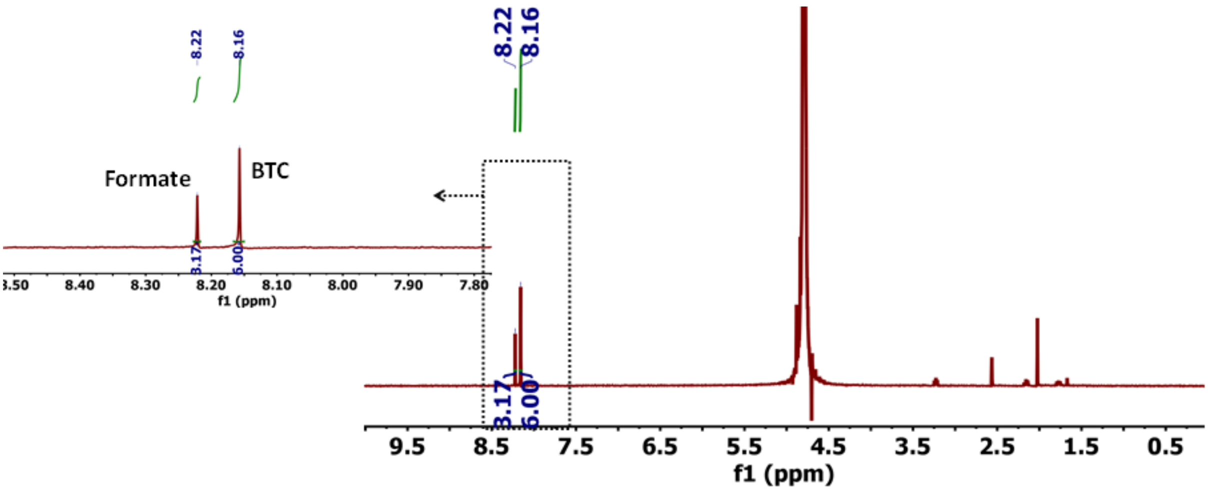

Supplementary Fig. 42 ^{1}H NMR spectra. ^{1}H NMR spectra for alkaline digested microporous MOF-808-PBA in KOH/D_2O solution**.**

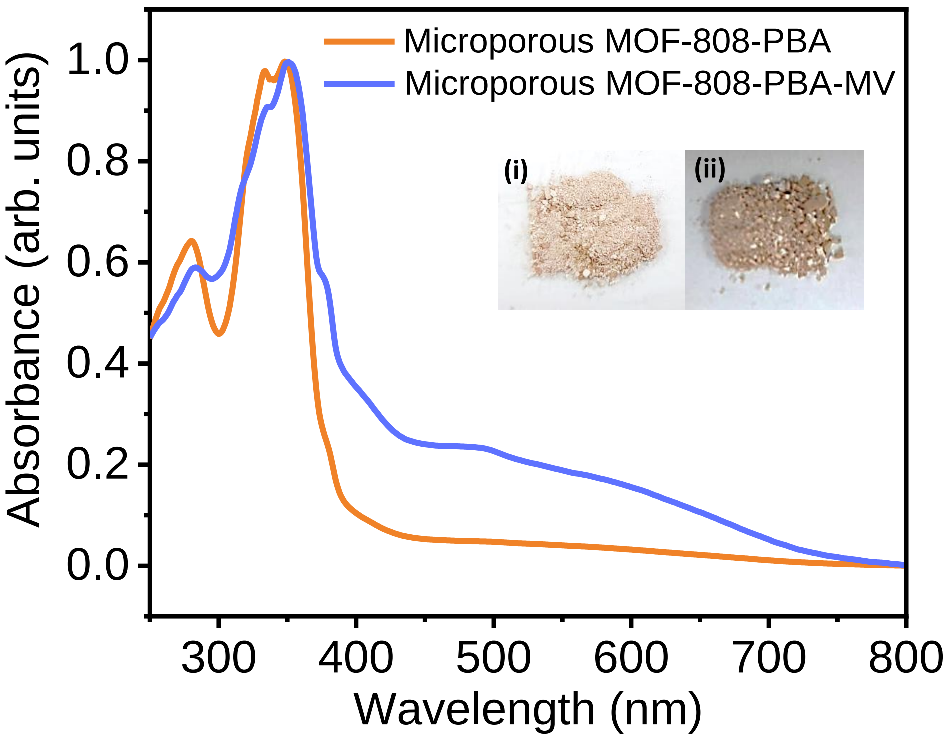

Supplementary Fig. 43 UV-vis spectra. UV-vis spectra of microporous MOF-808-PBA and MOF-808-PBA-MV. Inset showing the photograph of microporous MOF-808-PBA (i) and microporous MOF-808-PBA-MV (ii).

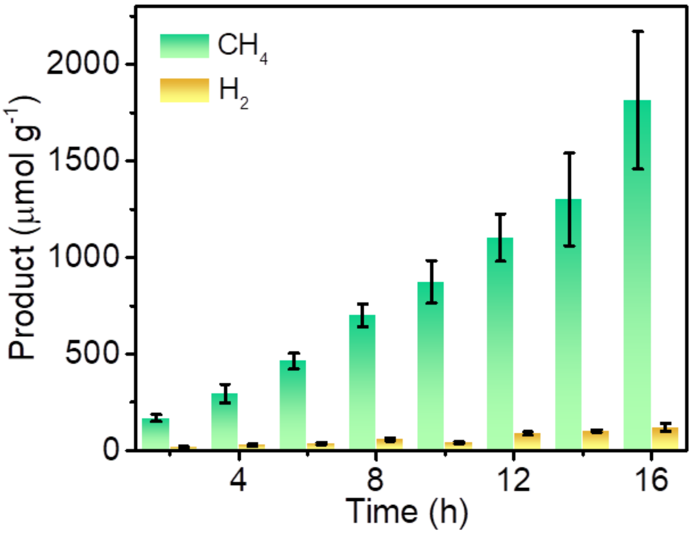

Supplementary Fig. 44 The CO_2RR activity of microporous MOF-808-PBA-MV. The amount of CH_4 and H_2 evolution by microporous MOF-808-PBA-MV as a function of time under visible light irradiation in water medium using BNAH and TEA as a sacrificial electron donor; error bars mean ± standard deviations calculated from three independent measurements.

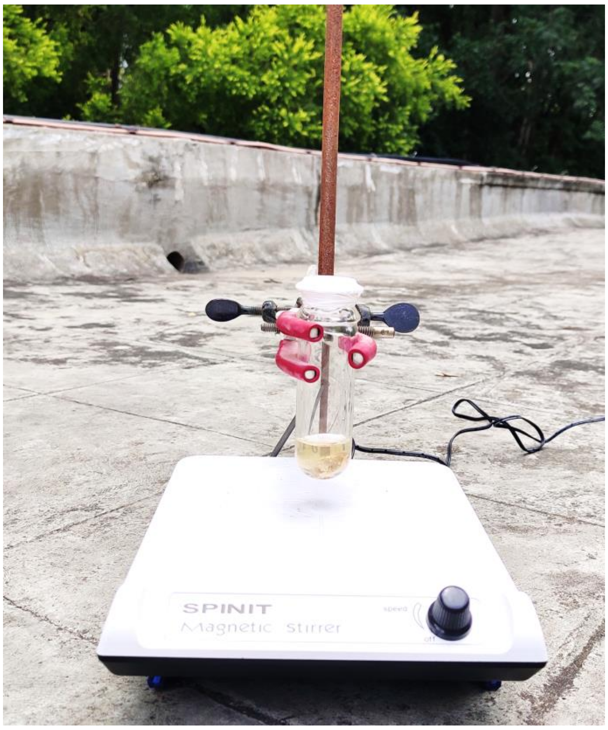

Supplementary Fig. 45 Sunlight experiment sepup for photocatalytic CO_2RR. Experimental set-up and environment for sunlight-driven CO_2RR.

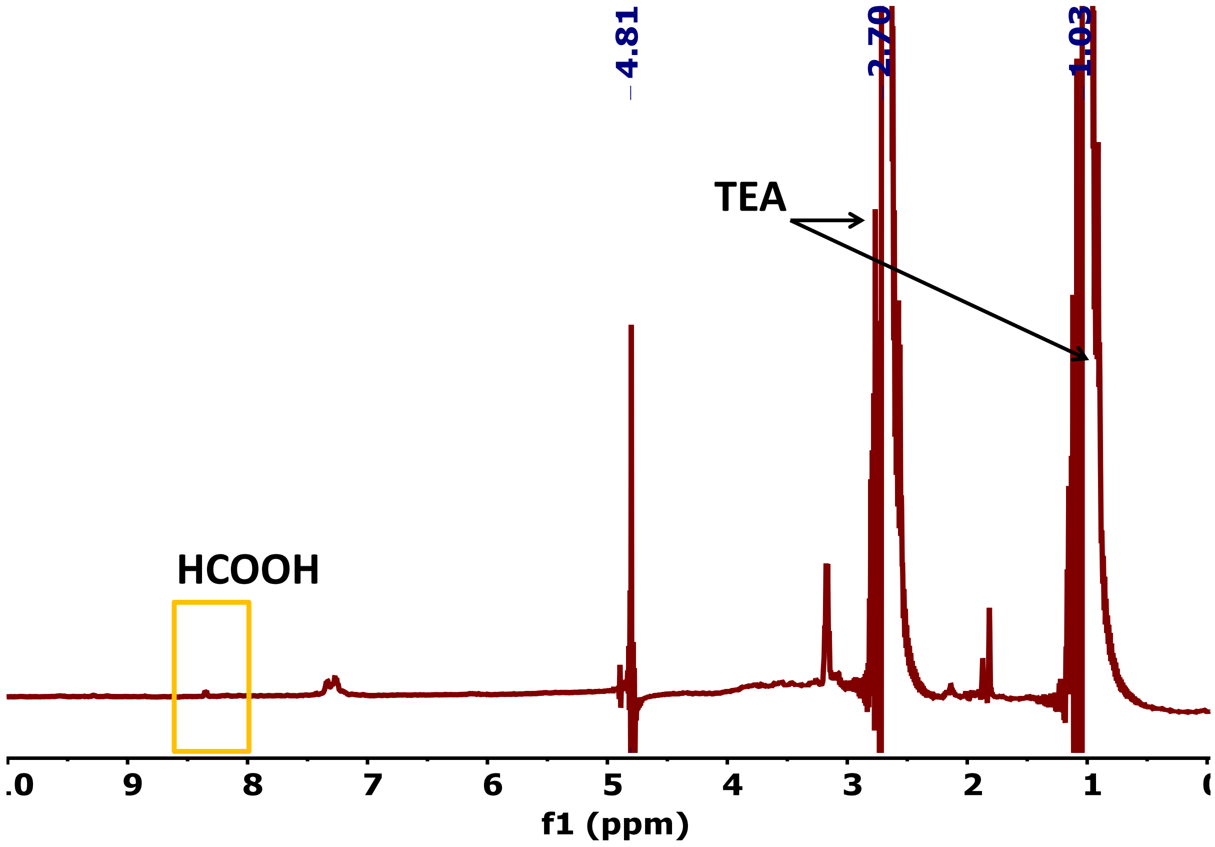

Supplementary Fig. 46 ^{1}H NMR spctrum towards liquid product analysis of CO_2RR by using MOF-808-PBA-MV as catalyst. A representative ^{1}H NMR spectrum of liquid phase generated from CO_2 reduction catalyzed by MOF-808-PBA-MV (aqueous medium).

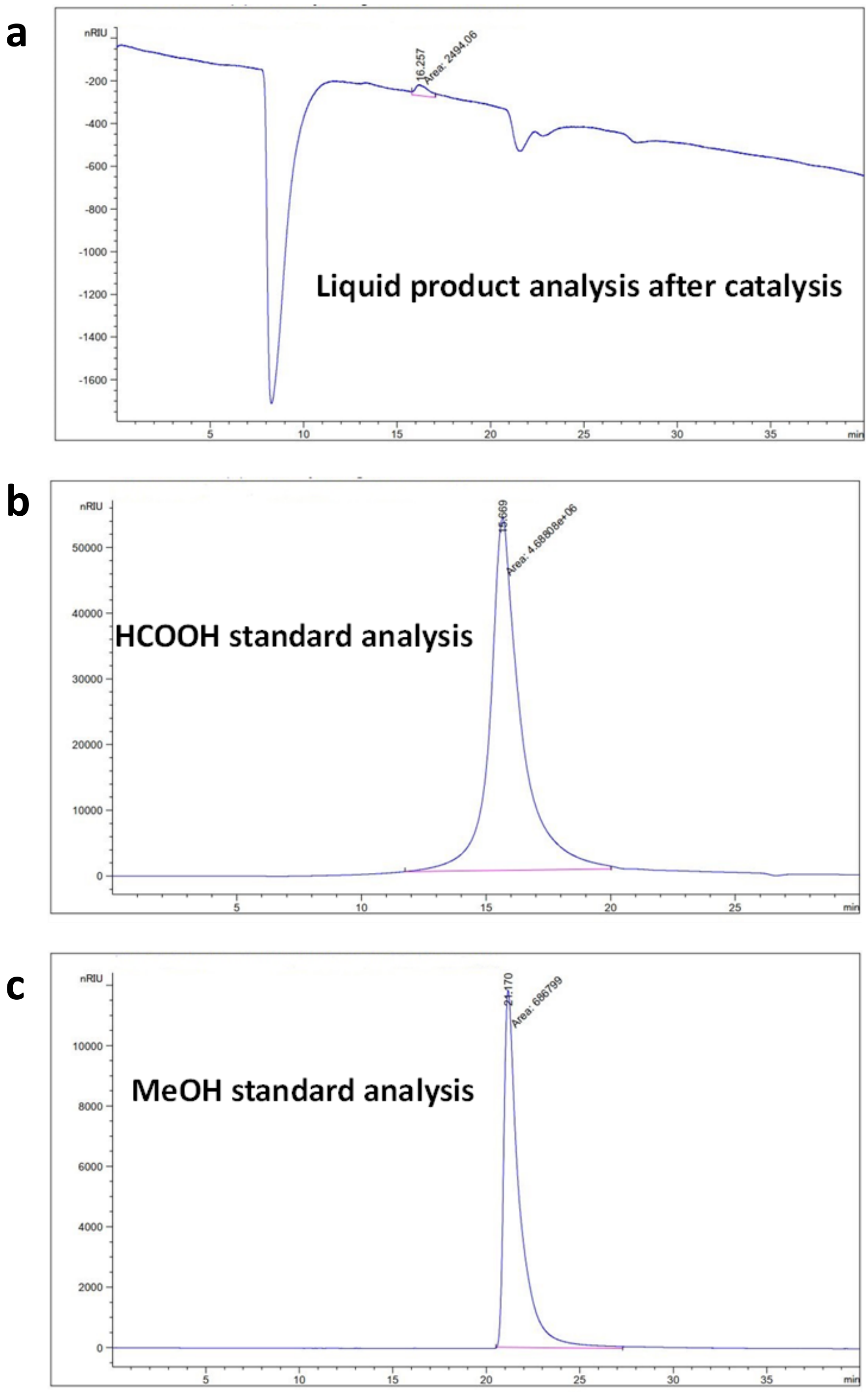

Supplementary Fig. 47 High Performance liquid chromatography (HPLC) spectra towards liquid product analysis of CO_2RR. a HPLC spectrum of the liquid phase from the reaction system of MOF-808-PBA-MV after visible-light irradiation for 16 h. **b** HPLC spectrum of standard HCOOH sample. **c.** HPLC spectrum of standard MeOH sample.

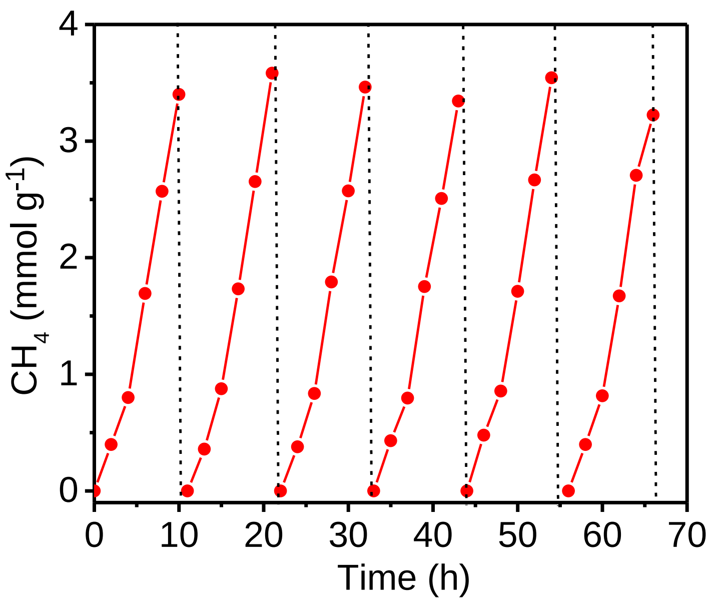

Supplementary Fig. 48 Cycling measurement for photoreduction of CO_2RR. [NOTE: we performed six consecutive catalytic cycles, showed consistent reduction activity with a total of 66 h of irradiation time. 3 mg sample was used for this measurement; when a new cycle started, the reaction medium (H_2O) along with BNAH and TEA purged with pure CO_2.]

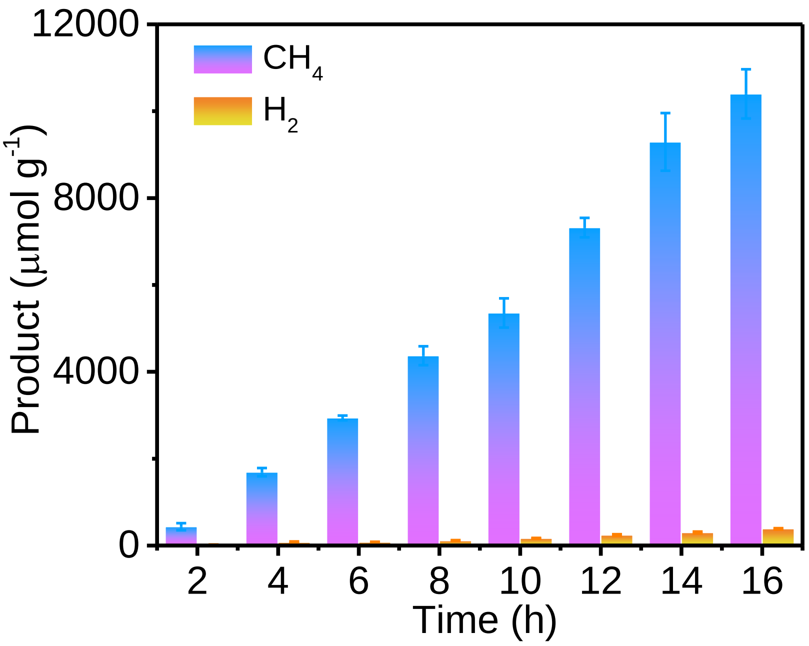

Supplementary Fig. 49 The CO_2RR activity of MOF-808-PBA-MV by using CO as purging gas. The amount of CH_4 and H_2 evolution by MOF-808-PBA-MV by reducing CO as a function of time under visible light irradiation in water medium using BNAH and TEA as a sacrificial electron donor. Error bars mean ± standard deviations calculated from three independent measurements.

Post catalytic analysis: The PXRD pattern of MOF-808-PBA-MV after catalysis further manifested that the crystallinity of MOF-808-PBA-MV remained intact. Corresponding TEM images of the recovered sample demonstrated the retention of the octahedral morphology as of pristine MOF. The post-catalysis UV-vis and FT-IR spectra analysis showed the retention of similar absorption spectra and stretching frequency compared to as-synthesized MOF-808-PBA-MV.

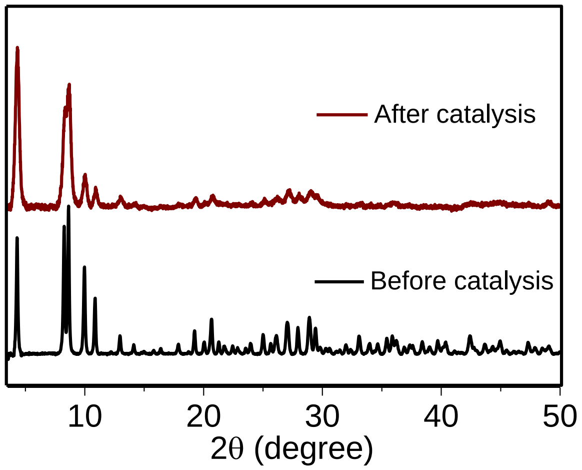

Supplementary Fig. 50 PXRD pattern after catalysis. PXRD pattern of MOF-808-PBA-MV after six consecutive catalytic cycles.

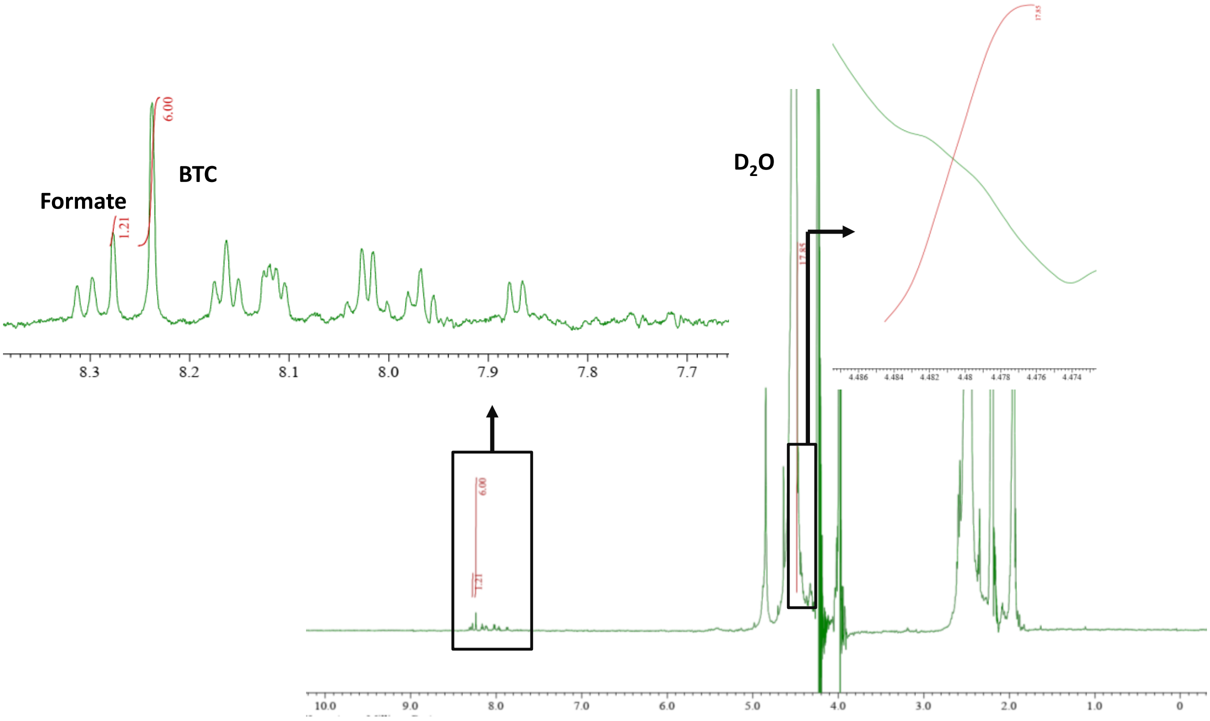

Supplementary Fig. 51 ^{1}H NMR spectrum. ^{1}H NMR spectrum of digested MOF-808-PBA-MV in KOH/D_2O/DMSO-d_6 after catalysis.

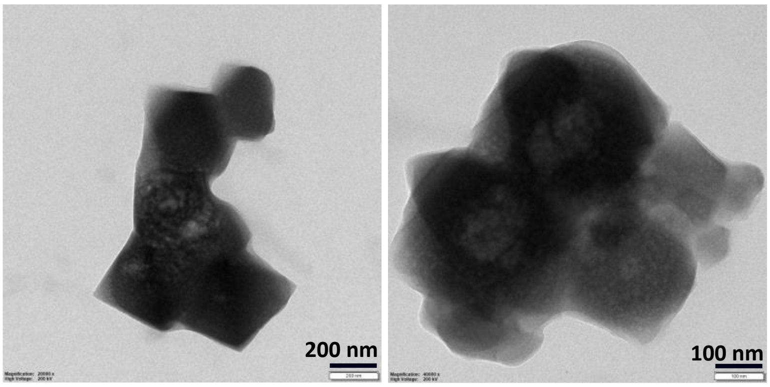

Supplementary Fig. 52 Morphological study after catalysis. TEM images of MOF-808-PBA-MV after catalysis.

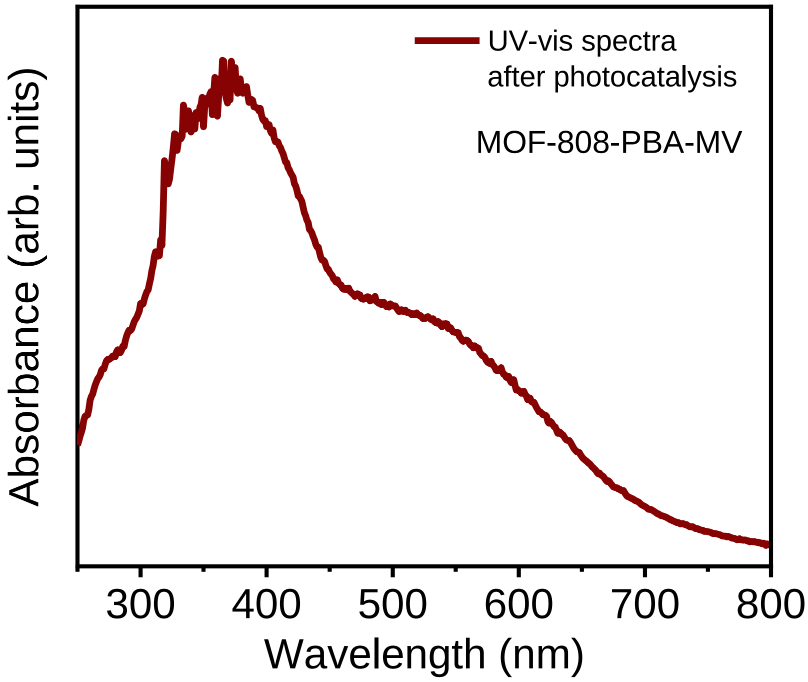

Supplementary Fig. 53 Optical property of the catalyst after CO_2RR. UV-vis spectra of MOF-808-PBA-MV after photocatalysis.

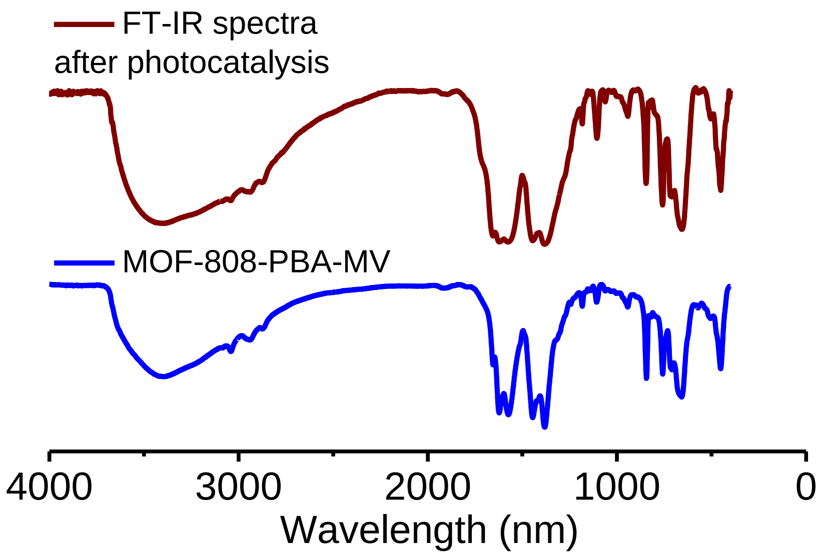

Supplementary Fig. 54 FT-IR spectra after catalysis. FT-IR spectra of MOF-808-PBA-MV after photocatalysis.

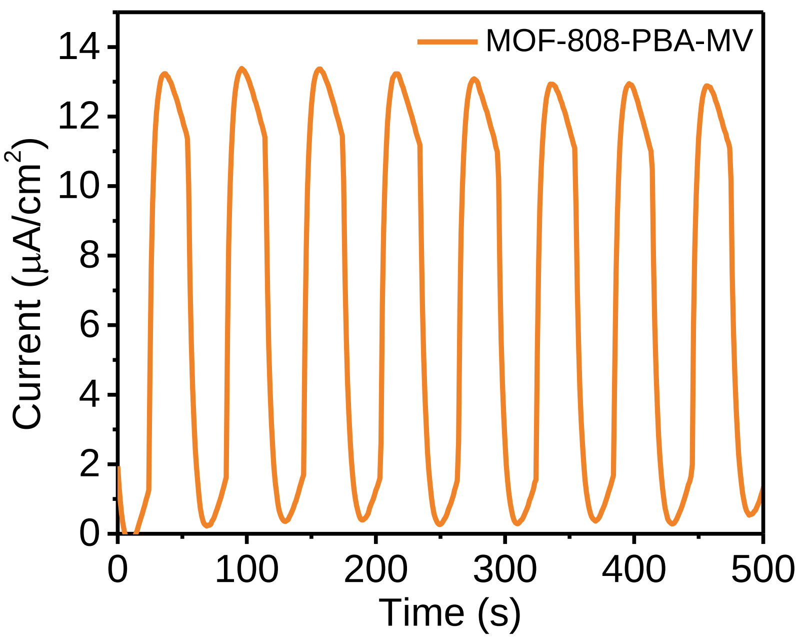

Supplementary Fig. 55 Photocurrent experiment of MOF-808-PBA-MV using sacrificial electron donor. Transient photocurrent responses of MOF-808-PBA-MV along with sacrificial electron donor in 0.2 M Na_2SO_4 aqueous solution under visible-light irradiation.

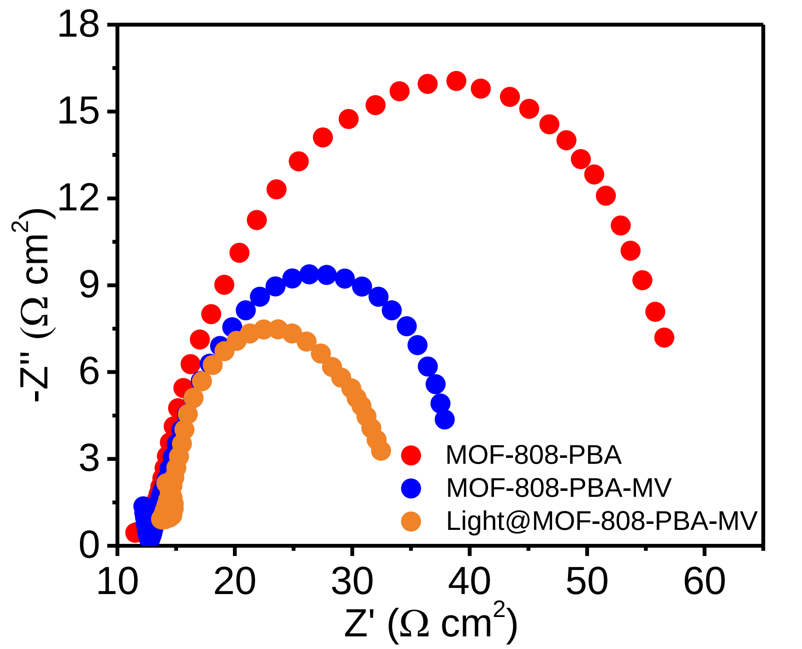

Supplementary Fig. 56 Impedence spectra to understand the charge transfer behaviour. EIS Nyquist plot of MOF-808-PBA and MOF-808-PBA-MV in the presence and absence of light.

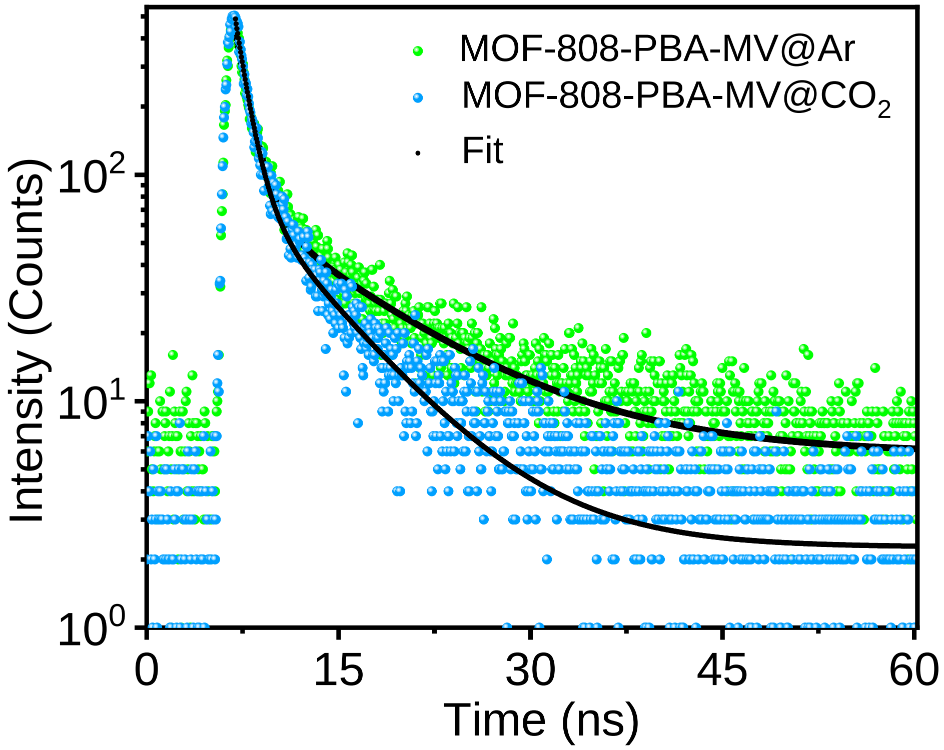

Supplementary Fig. 57 Lifetime experiment. Time-resolved photoluminescence decay of MOF-808-PBA-MV under CO_2 and Ar atmosphere.

Supplementary Table 8: Summary of Time-resolved photoluminescence (TRPL) decay of MOF-808-PBA-MV in CO_2 and Ar atmosphere.

**Compounds**	τ_1	τ_2	τ_3	τ_4	τ_{av}
**MOF-808-PBA-MV@Ar**	0.89 ns (79.40 %)	5.03 ns (20.6 %)	-	-	1.74 ns
**MOF-808-PBA-MV@CO_2**	0.67 (53.3)	0.33 (37.9 %)	4.97 (8.8%)	-	0.92 ns

Femtosecond transient absorption (TA) spectroscopy. The transient absorption spectroscopy of MOF-808-PBA-MV dispersed in acetonitrile solvent was carried out using a pump-probe set-up described earlier.[30] Briefly, a 1 kHz femtosecond amplified Ti-Sapphire laser (50 fs, 3 mJ) was used for the generation of the pump pulse (350 nm) in an optical parametric amplifier (TOPAS, Light Conversion, Lithuania). 350 nm laser was selected to excite pyrene molecule at the edge of $S_1 \leftarrow S_0$ transition and minimize the $S_2 \leftarrow S_0$ transition.[31] The white probe pulse was generated by focussing a small fraction of 800 nm laser pulse into a rotating calcium fluoride plate. To avoid sample decomposition, the sample cell was rotated during the experiment.

TA measurements were also carried out with pyrene solution in acetonitrile, and the results are shown in Supplementary Fig. 58. The absorbance of pyrene at the excitation wavelength (350 nm) was kept similar to the solution of MOF-808-PBA-MV. The TA spectrum of Py consists of a strong band at 470 nm with a shoulder at 510 nm. These two bands are characteristic of the S_1 state of pyrene.[32] The broad band observed in the region 600-750 nm is due to the pyrene excimer.[33] TA experiment of Pyrene solution was also performed in the presence of MV, and results are shown in Supplementary Fig. 58. It is evident from the figure that the TA spectra, as well as the decay kinetics of pyrene, remain unaltered in the presence of MV, which is about six times more concentrated than pyrene. This result further confirms that even with relatively higher concentration of MV, no charge transfer takes place in homogeneous solution.

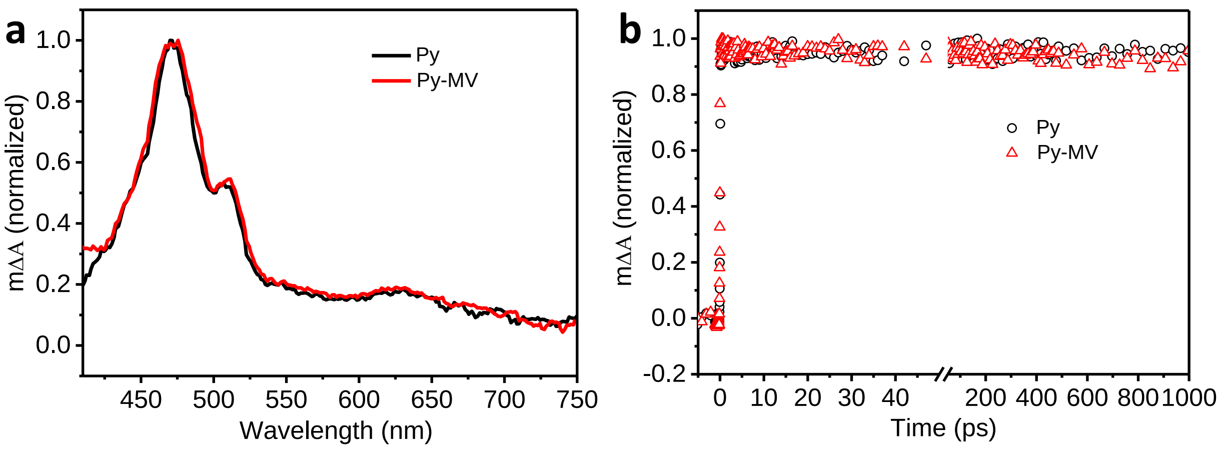

Supplementary Fig. 58 Transient spectra a and decay traces at 470 nm **b** of pyrene (50 μM) in absence and presence of MV (300 μM) in acetonitrile.

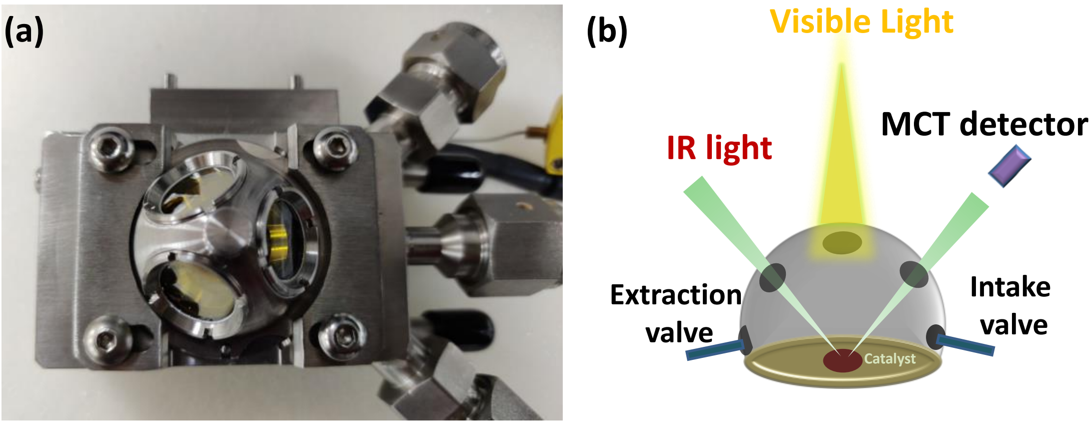

Supplementary Fig. 59 a Image of the set-up utilized for *in situ* diffuse reflectance infrared Fourier transform spectroscopy. **b** Schematic representation of the set-up utilized for *in situ* diffuse reflectance infrared Fourier transform spectroscopy.

Probable reaction intermediates obtained from *in-situ* DRIFT and DFT calculation

The most likely reaction pathway of CO_2 to CH_4 conversion be proposed as,

$$CO_2 \rightarrow CO_2^* \quad (1)$$

$$CO_2^* + e^- + H^+ \rightarrow COOH^* \quad (2)$$

$$COOH^* + e^- + H^+ \rightarrow CO^* + H_2O \quad (3)$$

$$CO^* + e^- + H^+ \rightarrow CHO^* \quad (4)$$

$$CHO^* + e^- + H^+ \rightarrow CH_2O^* \quad (5)$$

$$CH_2O^* + e^- + H^+ \rightarrow CH_3O^* \quad (6)$$

$$CH_3O^* + e^- + H^+ \rightarrow CH_3OH^* \quad (7)$$

$$CH_3OH^* + e^- + H^+ \rightarrow CH_3{}^* + H_2O \quad (8)$$

$$CH_3{}^* + e^- + H^+ \rightarrow CH_4 \uparrow + H_2O \quad (9)$$

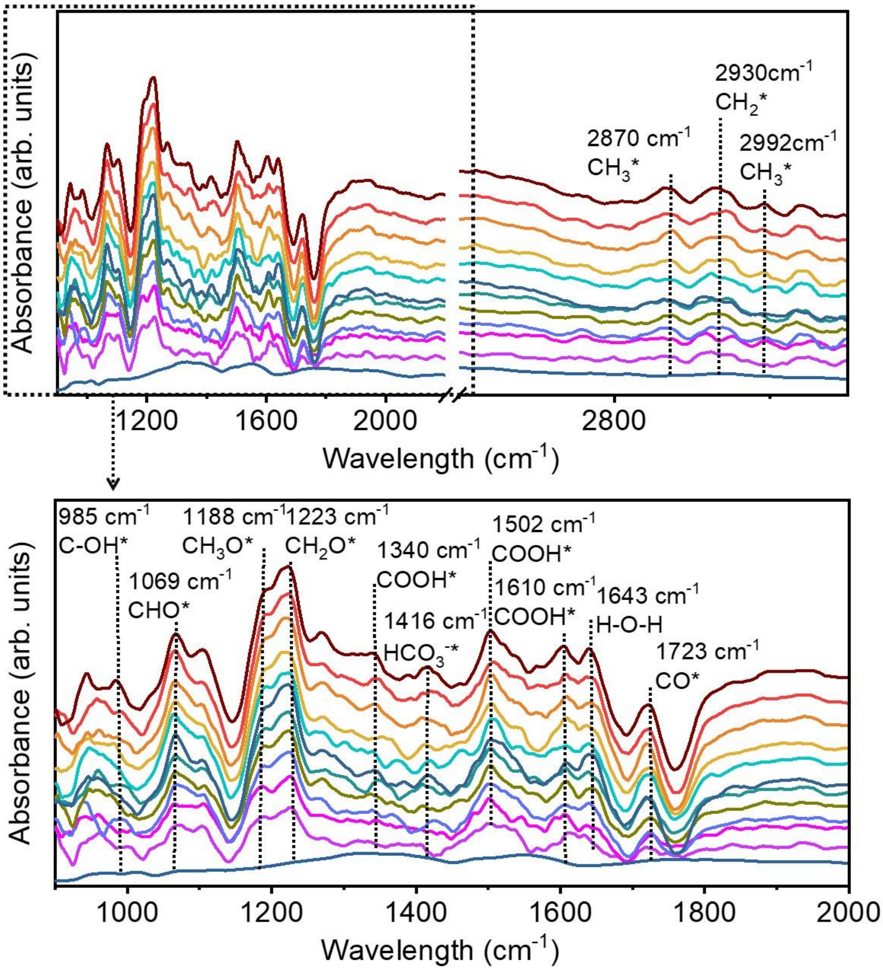

Supplementary Fig. 60 DRIFT study to understand the reaction intermediate. Time-dependent *in situ* FT-IR spectra of photocatalytic CO_2RR in a mixture of CO_2 and H_2O vapour, TEA and BNAH over MOF-808-PBA-MV in the dark and under visible light irradiation. Irradiation time 10, 20, 30, 40, 50, 60, 70, 80 min respectively.

In-situ DRIFT process purging the system with CO_2 in presence of BNAH and TEA:

In-situ DRIFT experiment was performed in the presence of BNAH and TEA (Supplementary Fig. 59**)**. To perform the experiment, we dispersed the catalyst along with BNAH and thereafter coated it over a glass surface. Before starting the experiment, TEA (10 μL) was added to the glass surface. After that, a similar procedure was followed, as mentioned previously. The peaks that appeared at 1314 and 1502 cm^{-1} after introducing CO_2 and H_2O vapour into the reactor could be assigned to the monodentate carbonate group (m-CO_3^{2}).[6,34] The peak at 1436 cm^{-1} corresponds to HCO_3^- group.[21] Peaks appearing after photo-irradiation at 1343 and 1610 cm^{-1} correspond to COOH* intermediate, which is a crucial intermediate during the photochemical conversion of CO_2 to CH_4.[4,21] Notably, a strong band appeared at 1723 cm^{-1}, which could be attributed to the bending vibration of C=O.[35] The absorption band at 985, 1069, 1188, and 1223

cm-1 are assigned to the characteristic bands of C-OH*, CHO*, CH_3O*, and CH_2O*, respectively.[21,36,37] The peak at 1643 cm-1 belongs to H_2O.[38] Besides, the band at 2870 and 2992 cm-1 corresponds to C-H stretching frequency of CH_3* group, and 2930 cm-1 corresponds to C-H stretching frequency of CH_2* group.[35] Hence a similar pathway was followed in the presence of BNAH and TEA, as mentioned above.

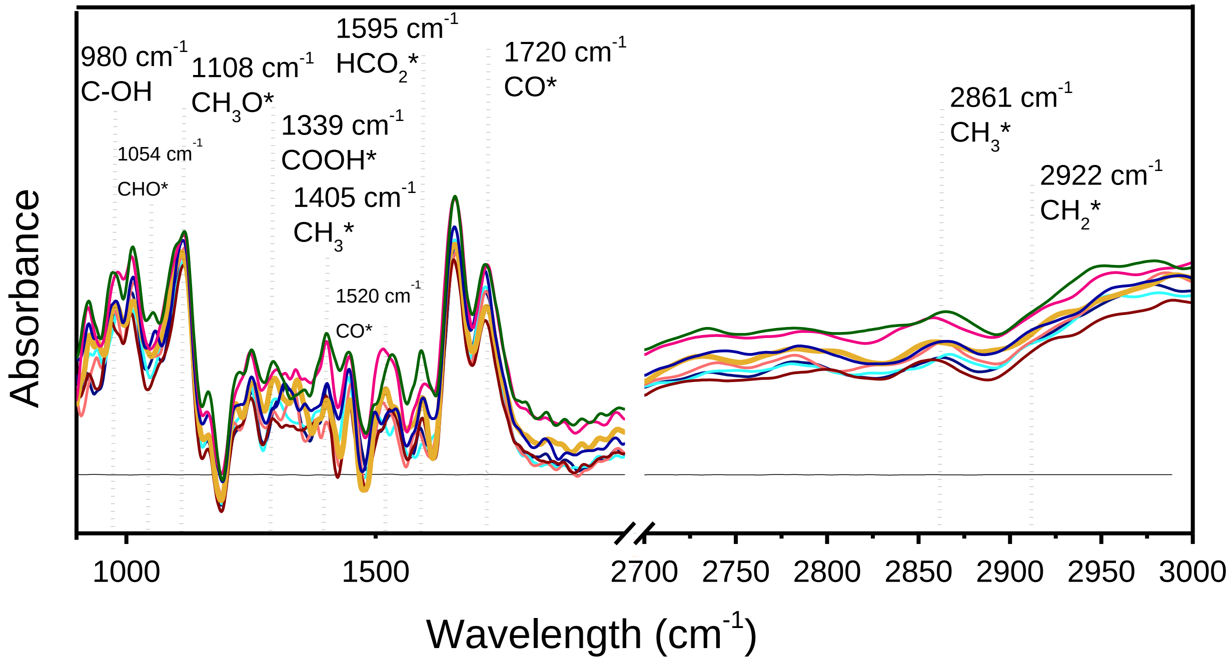

Supplementary Fig. 61 DRIFT study to understand the reaction intermediate. Time-dependent *in situ* FT-IR spectra of photocatalytic $^{13}CO_2$RR in a mixture of $^{13}CO_2$ and H_2O vapour, TEA and BNAH over MOF-808-PBA-MV in the dark and under visible light irradiation. Irradiation times 10, 20, 30, 40, 50, 60, 70, 80 min, respectively.

In-situ **DRIFT process purging the system with $^{13}CO_2$ in presence of BNAH and TEA:**

DRIFT study was also performed with $^{13}CO_2$, which clearly depicted the possible reaction intermediates in this process (Supplementary Fig. 60). Peaks appearing after photo-irradiation at 1339 cm-1 correspond to COOH* intermediate, which is a crucial intermediate during the photochemical conversion of CO_2 to CH_4.[4,21] Notably, a strong band appeared at 1720 cm-1, which could be attributed to the bending vibration of C=O.[35] Importantly, the absorption band at 980, 1095, 1595, and 1101 cm-1 belongs to C-OH*, CHO*, CH_2O* and CH_3O* species, respectively, which are pivotal intermediate for CH_4 formation.[21,36,37,39] The band that appeared at 1405 corresponds to the deformation vibrations of CH_3* and CH_4 group.[35,40] Besides, the band at 2861 cm-1 corresponds to C-H stretching frequency of CH_3* group and 2922 cm-1 correspond to C-H stretching frequency of CH_2* group.[35] All the bands corresponding to the

crucial intermediate for CH_4 formation were red-shifted compared to when we purged the system with $^{12}CO_2$, which is due to the isotopic effect.[39]

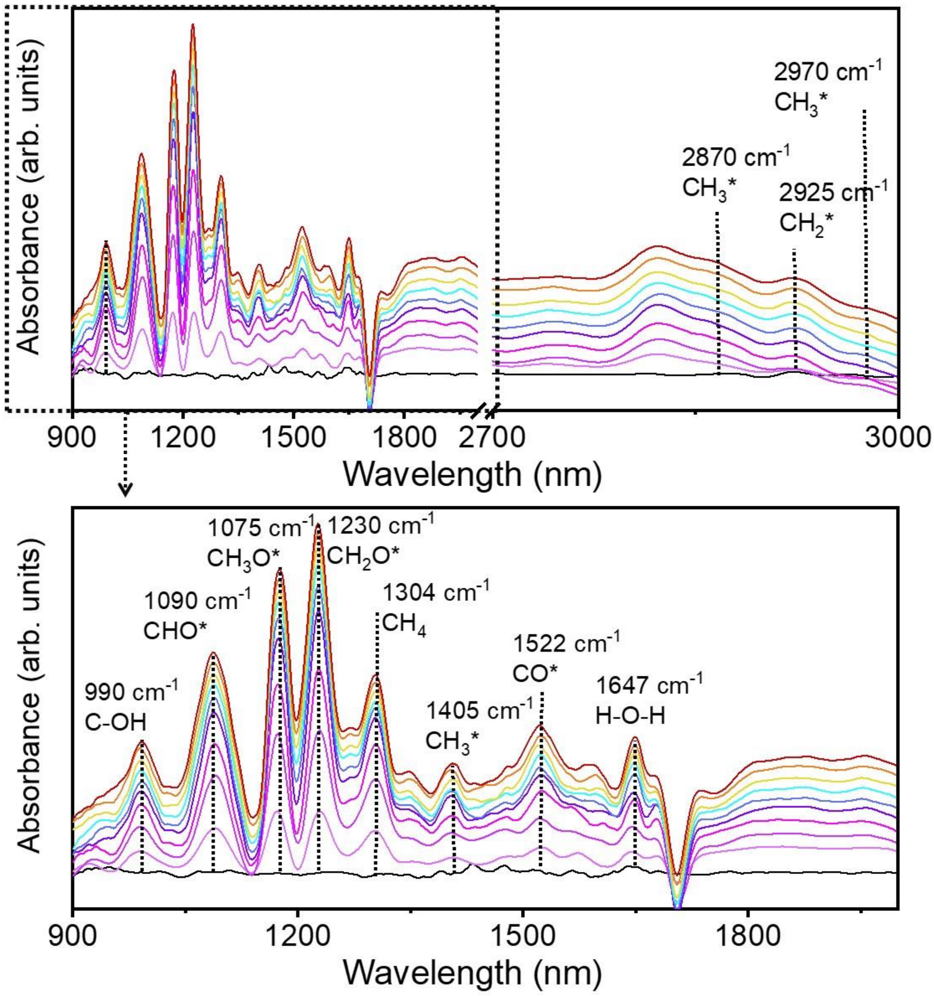

Supplementary Fig. 62 DRIFT study to understand the reaction intermediate. Time-dependent *in situ* FT-IR spectra of photocatalytic CO reduction reaction in a mixture of CO_2 and H_2O vapour, TEA and BNAH **over MOF-808-PBA-MV** in the dark and under visible light irradiation. Irradiation time 10, 20, 30, 40, 50, 60, 70, 80 min, respectively.

***In-situ* DRIFT process purging the system with CO in presence of BNAH and TEA:**

DRIFT study was also performed with CO. After photo irradiation of the MOF-808-PBA-MV for 10 min resulted in the appearance of several new peaks. The absorption band at 1522 cm^{-1} belongs to adsorbed CO .[38] The absorption band at 990, 1090, 1175, and 1230 cm^{-1} are assigned to the characteristic bands of C-OH*, CHO*, CH_3O*, CH_2O*, respectively .[21,36,37] The band that appeared at 1405 and 1304 cm^{-1} corresponds to the deformation vibrations of CH_3* and CH_4 group. [35,40] Moreover, the peak at 1647 cm^{-1} corresponds to H_2O.[38] Besides, the band at 2870 and 2970 cm^{-1} corresponds to C-H stretching frequency of CH_3* group and 2925 cm^{-1} corresponds to C-H stretching frequency of CH_2* group.[35]

Hence the most likely reaction pathway of CO to CH_4 conversion be proposed as,

$$CO \rightarrow CO^* \quad (1)$$

$$CO^* + e^- + H^+ \rightarrow CHO^* \quad (2)$$

$$CHO^* + e^- + H^+ \rightarrow CH_2O^* \quad (3)$$

$$CH_2O^* + e^- + H^+ \rightarrow CH_3O^* \quad (4)$$

$$CH_3O^* + e^- + H^+ \rightarrow CH_3OH^* \quad (5)$$

$$CH_3OH^* + e^- + H^+ \rightarrow CH_3{}^* + H_2O \quad (6)$$

$$CH_3{}^* + e^- + H^+ \rightarrow CH_4\uparrow + H_2O \quad (7)$$

Reductive quenching or oxidative quenching of $[PBA^+\text{-}MV^+]^{2+}$ by BNAH:

To understand whether the photoexcited $[PBA^+\text{-}MV^+]^{2+}$ was reductively quenched by BNAH or oxidatively quenched by the Zr cluster to initiate the catalytic cycle, we recorded the luminescence spectra of MOF-808-PBA-MV (50 mM) with different equivalent additions of BNAH. The emission of MOF-808-PBA-MV was effectively quenched by BNAH, which demonstrated that the photoexcited $[PBA^+\text{-}MV^+]^{2+}$ undergoes reductive quenching by BNAH.

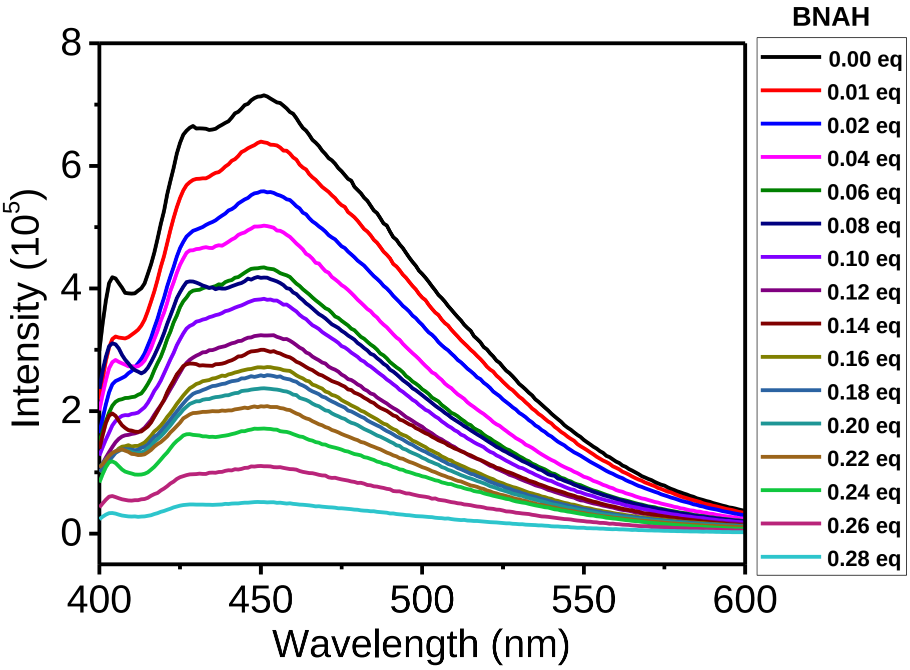

Supplementary Fig. 63 Understand the reductive/oxidative quenching precess of photosensitizer. Emission spectra of MOF-808-PBA-MV (50 μM) after the addition of different equivalent of BNAH in MeOH.

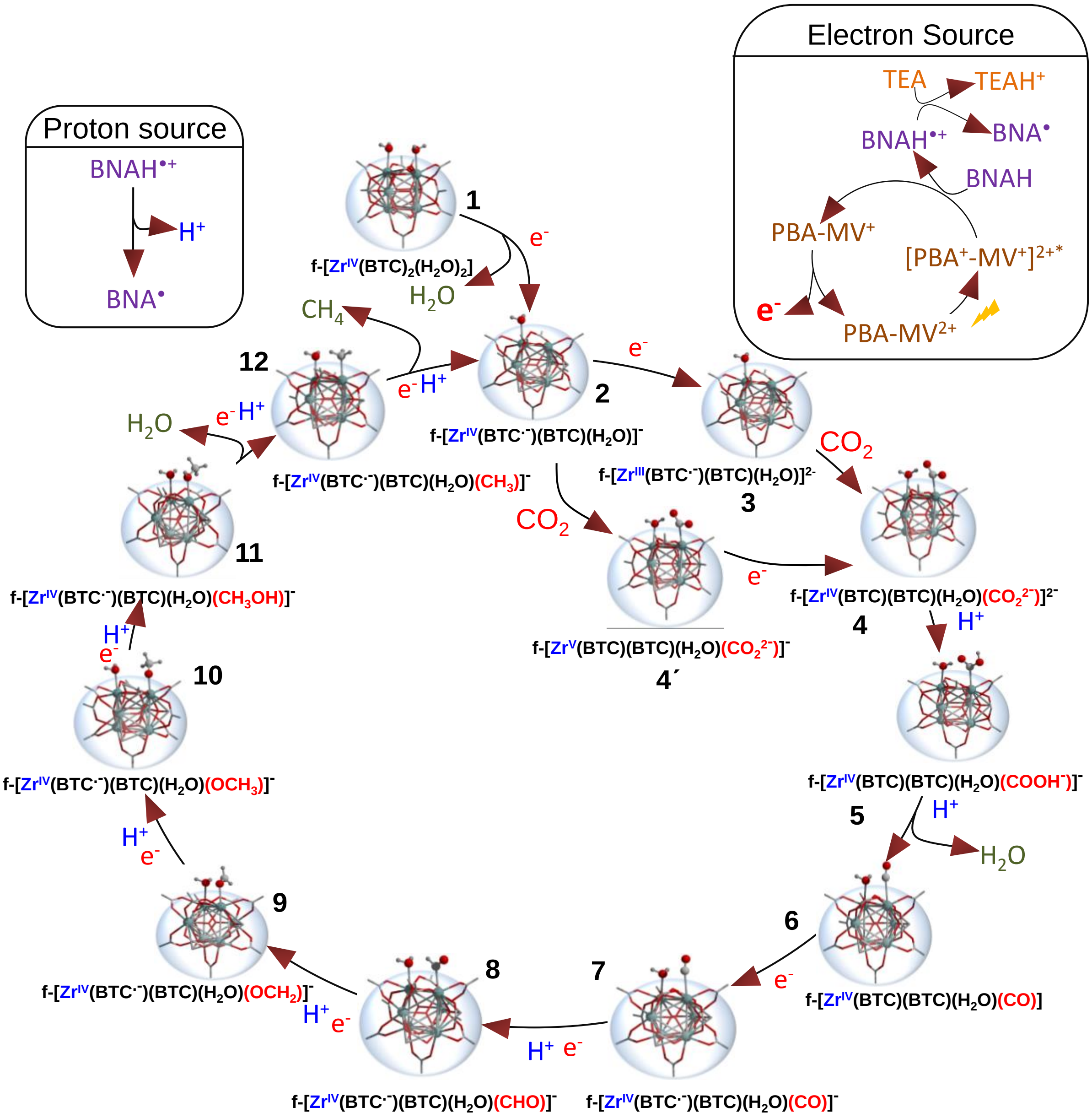

Supplementary Fig. 64 Proposed CO_2 reduction catalytic mechanism for MOF-808-PBA-MV. Proposed reaction mechanism for the CO_2-to-CH_4 photo-conversion on MOF-808-PBA-MV system in water. (f= Zr-cluster fragment).

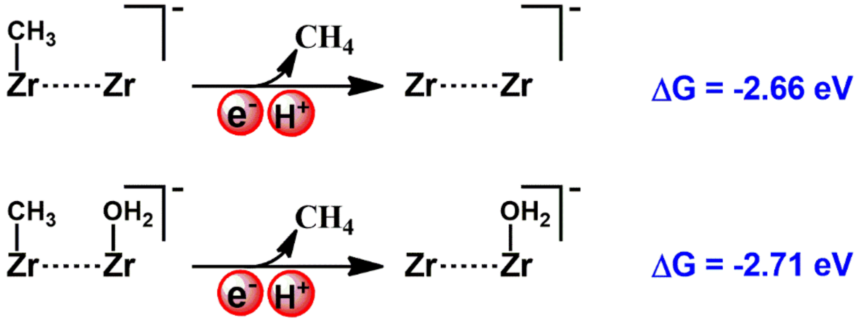

Supplementary Fig. 65 DFT study. The role of proximal H_2O investigated by DFT calculation.

Cartesian coordinates of the computed structures

Coordinates are given in standard XYZ format

Supplementary Table 9. DFT-optimized geometry of f-[$Zr^{IV}(BTC)_2(H_2O)_2$] (singlet), computed at the B3LYP-D3/ LANL2DZ (Zr)/ 6-31G* (HCO) level in water solvent using PCM.

Atom	x	y	z
O	8.29243400	1.11061100	-2.01079500
O	7.26177000	2.85733100	-3.93027100
O	8.29106000	4.79217900	-3.39665500
O	9.36239300	-0.13707900	-0.54518300
C	8.46402600	-0.04672000	-1.37646200
C	7.47725100	3.76054200	-3.13318500
C	7.52648700	-1.17747400	-1.62873000
C	6.87601600	3.81227200	-1.77397100
C	6.15213100	-0.95744100	-1.77411400
C	8.02741100	-2.48264900	-1.54641200
C	5.75682100	3.01400600	-1.53427700
C	7.40714900	4.58824600	-0.74172300
H	5.73936700	0.04575400	-1.80469500
C	5.27368200	-2.04793200	-1.76574400
H	9.08938100	-2.63980200	-1.40366600
C	7.15274600	-3.56967300	-1.62317700
H	5.32491300	2.44357900	-2.34709900
C	5.17811700	2.95920300	-0.26471300
H	8.25132100	5.24358300	-0.92127700
C	6.86963900	4.49614900	0.54721500
C	5.77371800	-3.34785700	-1.69293500
C	3.79863400	-1.80375100	-1.70386200
C	7.63061400	-4.98121300	-1.57400500
C	5.76014300	3.67521800	0.79063100
C	3.92326300	2.15576900	-0.09383400
C	7.47565800	5.36877600	1.61255000
H	5.09444000	-4.19204500	-1.67678400
O	3.11212700	-2.64585400	-1.04732600

O	3.35089300	-0.75995600	-2.26764100
O	8.96666100	-5.07261900	-1.41916300
O	6.90234500	-5.95168600	-1.66024700
H	5.32628600	3.60905100	1.78206600
O	3.43074900	1.68296200	-1.16877200
O	3.42932000	2.01606500	1.06430600
O	7.59062200	4.85584600	2.84032100
O	7.83756200	6.50608100	1.36972300
Zr	1.33622700	-2.35469000	0.30644600
Zr	1.57952300	0.54663900	-1.73688000
Zr	1.52650000	0.94288300	1.79284400
Zr	-1.49607400	-1.21820100	-1.56548400
Zr	-1.53455400	-0.93374500	1.85334900
O	3.16709000	-2.32311700	1.65620900
O	0.78660000	-1.54788400	-1.77146800
O	-0.65572600	-1.91215000	0.22708300
O	0.73958300	-1.18531800	2.17148800
O	1.85165100	-0.29926800	0.12582800
O	0.67335300	-3.85284900	1.84358500
O	0.67256900	-4.12223500	-0.91709300
Zr	-1.36448700	1.82321800	-0.09134000
O	-0.48019900	0.64552300	-1.58486400
O	0.95230500	2.08996200	-0.20198300
O	1.01448700	0.16407500	-3.88282200
O	7.52324600	-4.75001800	1.49141000
O	3.21753800	-0.26091900	2.57313600
O	-0.50175500	0.87563600	1.52563800
O	1.09454200	3.07304100	2.35450600
O	0.90451800	0.81729600	3.95387400
O	7.40243300	2.19776200	2.93003500
O	9.03047600	1.64002500	1.53143800
O	9.46212600	-3.59671600	1.52609000
O	-3.42477300	-2.31303500	-0.96028000
O	-3.05663000	-0.01111400	-2.56110900
O	-2.29115300	-0.04724100	0.03595500
O	-0.90175200	-1.06024300	-3.80546000
O	-1.14905800	-3.41698100	-2.08620600
O	-3.32390900	0.34466700	2.54357600
O	-3.24824500	-2.23139200	1.28553400
O	-1.17017800	-2.97413600	2.84241200
O	-1.08171600	-0.29673800	4.00183800
C	3.77534000	-1.30972800	2.11497900
C	-0.31177200	-3.87580600	2.64428400
C	-0.31549100	-4.28184600	-1.69774000
O	-3.14417400	2.02398300	-1.59846500
O	-3.03104000	2.20149600	1.30563600
O	-0.75564500	3.68499900	1.18586300
C	0.01894400	-0.44405900	-4.39851200
C	8.24755100	-3.61574700	1.59582700
C	0.15937600	3.86858500	2.03411000
C	-0.13351500	0.35541900	4.52022500
C	7.92772200	1.34978000	2.21197100
C	-3.90731700	-2.37232700	0.20871700
C	-3.65272300	1.06570200	-2.25477000
C	-3.74109800	1.40900400	2.00364900
C	5.26877100	-1.30282200	2.08440900
C	7.40251800	-2.40489600	1.79354500
C	7.31499500	-0.00385100	2.09013100
C	-5.37811700	-2.59999900	0.39803300
C	-5.07996000	1.14253100	-2.70747200

C	-5.18014400	1.80151700	2.14006200
C	6.00629800	-2.47008000	1.86106900
C	5.92443300	-0.07769600	2.21462700
C	8.05684600	-1.17040200	1.89394400
C	-6.18729300	-3.27326700	-0.52018600
C	-5.93613000	-2.09859900	1.57890800
C	-5.74915900	2.33871000	-2.97327900
C	-5.74334000	-0.07847600	-2.86875800
C	-5.98079100	1.44239000	3.22653700
C	-5.71826600	2.56983100	1.10256400
H	5.49086600	-3.41537900	1.74445600
H	5.34507300	0.82304800	2.37319500
H	9.13649600	-1.14880600	1.81696200
H	-5.76073000	-3.67151700	-1.43309600
C	-7.55291600	-3.45813800	-0.23967000
H	-5.30012200	-1.57305600	2.27925500
C	-7.29958800	-2.23859900	1.83619000
H	-5.24071000	3.28799500	-2.85150400
C	-7.07508400	2.30242600	-3.43998100
H	-5.21518400	-0.99732700	-2.64977000
C	-7.07800500	-0.11374300	-3.26738900
H	-5.56872800	0.85201400	4.03615100
C	-7.31735800	1.87432600	3.27826600
H	-5.08919000	2.83565300	0.26281200
C	-7.05703300	2.96061400	1.12945400
C	-8.11151000	-2.91886000	0.92712800
C	-8.39141600	-4.25012600	-1.17773100
C	-7.89278200	-1.62813500	3.05962900
C	-7.74600400	1.07794400	-3.55731400
C	-7.75530100	3.57961300	-3.81128900
C	-7.79621400	-1.41834100	-3.35010600
C	-7.85897600	2.61466700	2.21869000
C	-8.13804800	1.55295500	4.47573500
C	-7.63729300	3.72291200	-0.01438700
H	-9.16863500	-3.03086200	1.13619200
O	-9.57834100	-4.59426200	-0.66501000
O	-8.05537700	-4.58820200	-2.30971500
O	-6.97340400	-1.43289600	4.01805300
O	-9.06808500	-1.32187200	3.16839400
H	-8.79007800	1.02937700	-3.85229500
O	-8.65805200	3.53832600	-4.80097300
O	-7.53680800	4.65746200	-3.27325300
O	-6.94358300	-2.44494900	-3.47952500
O	-9.00815800	-1.53407800	-3.29052400
H	-8.89588100	2.92822300	2.23674000
O	-9.28611400	2.23791400	4.53095900
O	-7.81574400	0.75694900	5.35339500
O	-6.68609900	4.29469800	-0.76451600
O	-8.83253700	3.80338300	-0.24286700
H	0.16036300	4.83896500	2.55365500
H	-0.41791800	-4.79330600	3.24277300
H	-0.45195100	-5.30072600	-2.09088600
H	-0.02411400	-0.42763000	-5.49809500
H	1.09405800	-2.09503800	-2.51043600
H	1.05502700	-1.57243100	3.00229700
H	1.30080600	2.99081400	-0.29130700
H	7.64398100	1.09738800	-2.74330900
H	9.19910700	-6.02104000	-1.41064100
H	8.15132800	-5.48850800	1.37384900
H	9.25029900	0.96996500	0.83492100

H	7.52118100	3.86140700	2.84274500
H	8.66849600	4.65769200	-4.28792000
H	-8.65653200	2.67662400	-5.25431900
H	-7.07469100	4.60077700	-1.62722700
H	-9.76828300	1.94769800	5.32987800
H	-7.34451200	-0.81709500	4.69854400
H	-10.06902700	-5.08575100	-1.35255400
H	-7.41730700	-3.29450100	-3.28986100
H	-0.20686800	0.54291900	5.60234800
O	-1.14373700	3.73879000	-1.46964400
H	-1.94157200	3.83158300	-2.02273600
H	-1.07521400	4.54790100	-0.92988800
O	1.64601500	2.58056800	-2.96573200
H	2.51696200	3.01296200	-2.90064800
H	1.49432000	2.38993800	-3.91012300

Sum of electronic and thermal Free Energies = -6767.289180 (Hartree/Particle).

Supplementary Table 10. DFT-optimized geometry of f-$[Zr^{IV}(BTC^{\bullet -})(BTC)(H_2O)_2]^-$ (doublet), computed at the UB3LYP-D3/ LANL2DZ (Zr)/ 6-31G* (HCO) level in water solvent using PCM. The spin density plot is also shown below. (Isovalue = 0.0004)

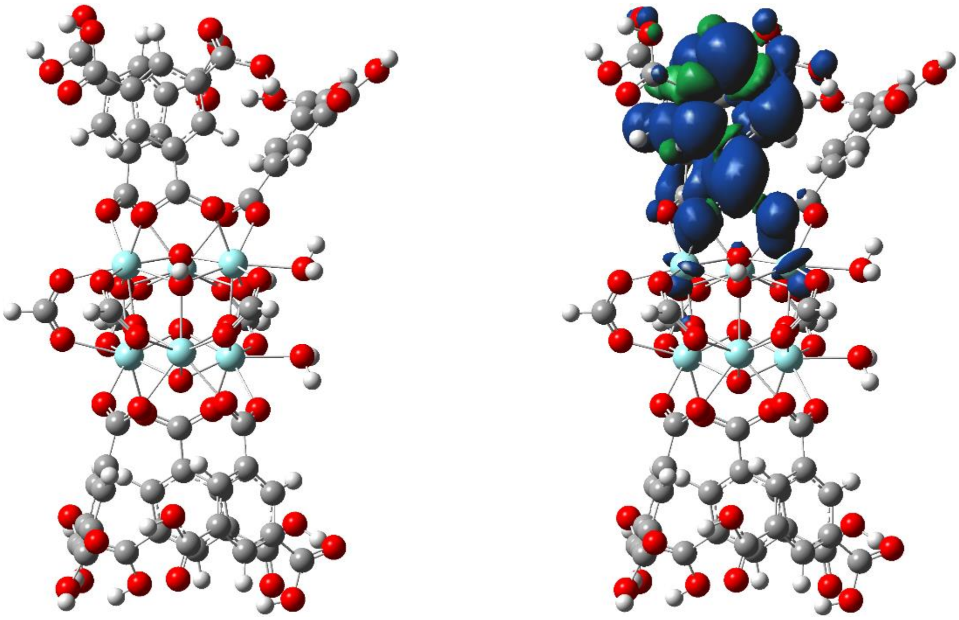

Spin Density Distribution

Atom	x	y	z
O	8.26900800	0.97071200	-1.96704400
O	7.30986000	2.56489700	-4.15233900
O	8.43581100	4.46794000	-3.70788300
O	9.39551900	-0.08966300	-0.39632200
C	8.46837600	-0.12313200	-1.21240200
C	7.54743400	3.50598800	-3.40945000
C	7.56356700	-1.27910600	-1.33527000
C	6.89934600	3.69185700	-2.08405200
C	6.21356100	-1.08476900	-1.61237700

C	8.05770700	-2.56091500	-0.96530700
C	5.76902900	2.92276500	-1.80472000
C	7.40004800	4.56316500	-1.11437600
H	5.82433700	-0.09906300	-1.84995900
C	5.27888900	-2.13711200	-1.42621900
H	9.10983700	-2.69728900	-0.75940100
C	7.14212200	-3.62860100	-0.86649800
H	5.36280600	2.27365900	-2.57033700
C	5.15158700	2.98713300	-0.55370700
H	8.25339000	5.19604100	-1.32773400
C	6.82033300	4.59680100	0.15835900
C	5.78120100	-3.41283000	-1.03587700
C	3.86275700	-1.91751100	-1.52556100
C	7.57609100	-5.01670000	-0.56821900
C	5.70280000	3.80049600	0.44511200
C	3.89441200	2.19337900	-0.34532000
C	7.39907000	5.56231400	1.15578800
H	5.09033600	-4.23587200	-0.90319300
O	3.06406500	-2.76942900	-0.94199600
O	3.39916300	-0.88719100	-2.18054200
O	8.91696800	-5.12880100	-0.39751400
O	6.83864500	-5.98643300	-0.49175100
H	5.24206500	3.82445800	1.42609400
O	3.40803500	1.67267000	-1.39813200
O	3.39624700	2.10554000	0.81593200
O	7.48943500	5.15579300	2.42388900
O	7.76453400	6.67631400	0.82309700
Zr	1.37155600	-2.37105600	0.34260000
Zr	1.65993700	0.35803900	-1.90836500
Zr	1.47983000	1.02919200	1.58556000
Zr	-1.44160000	-1.38986800	-1.65931900
Zr	-1.55480600	-0.86978700	1.71718500
O	3.17500800	-2.18715100	1.74423800
O	0.82419900	-1.72256800	-1.79422000
O	-0.64757300	-1.95362400	0.19213800
O	0.70709600	-1.07045300	2.10709200
O	1.84461400	-0.31254100	0.03931200
O	0.71242500	-3.76654700	1.97795900
O	0.66350900	-4.24233400	-0.73790900
Zr	-1.35687500	1.73082600	-0.41543300
O	-0.43921500	0.46040100	-1.79653100
O	0.93132200	2.02628900	-0.49138000
O	1.10245700	-0.22036400	-4.04554500
O	7.61695500	-4.51104600	2.36269100
O	3.15754500	-0.05489800	2.50177100
O	-0.55207800	0.93185700	1.30593900
O	0.99907000	3.20999000	1.99813000
O	0.83282200	1.05914600	3.74989900
O	7.26974300	2.53351200	2.70591500
O	8.96363200	1.85534400	1.44071400
O	9.52096300	-3.30490900	2.32136800
O	-3.40923100	-2.46612400	-1.00182900
O	-3.03257600	-0.26658000	-2.77182800
O	-2.29032900	-0.12304500	-0.16773500
O	-0.84027400	-1.39836600	-3.89257700
O	-1.10537600	-3.62462100	-2.03149900
O	-3.37531100	0.44722500	2.30243300
O	-3.26326700	-2.20095600	1.23384400
O	-1.17531800	-2.84537100	2.85327800
O	-1.13944700	-0.08056400	3.84470700

C	3.75334600	-1.13147700	2.14371800
C	-0.29555800	-3.74199400	2.74988500
C	-0.29231800	-4.45780400	-1.53930300
O	-3.08957500	1.82614900	-1.94137100
O	-3.08603300	2.19879800	0.90858400
O	-0.82985700	3.70942300	0.74723100
C	0.10665100	-0.85711000	-4.51916000
C	8.30376200	-3.34938500	2.29696500
C	0.06302300	3.96545200	1.60115200
C	-0.20970700	0.62261300	4.32828900
C	7.83538900	1.62072400	2.10092600
C	-3.90596300	-2.42511100	0.15911400
C	-3.63237600	0.80874300	-2.47404200
C	-3.76736800	1.50273600	1.72377200
C	5.24052400	-1.09642100	2.18719800
C	7.41746800	-2.15888900	2.21038200
C	7.25521300	0.25340600	2.11174600
C	-5.38184300	-2.62695600	0.35190300
C	-5.10456900	0.83927000	-2.74551600
C	-5.15224400	2.02429000	1.99188300
C	6.02061900	-2.26385400	2.19665900
C	5.86112900	0.15286200	2.17166500
C	8.03626400	-0.90629800	2.14012300
C	-6.18566200	-3.38177600	-0.50482300
C	-5.94824000	-2.02029100	1.47768800
C	-5.84300000	2.02323400	-2.77632600
C	-5.75036500	-0.38937600	-2.91694200
C	-5.88926600	1.67787800	3.12718100
C	-5.69468600	2.91578800	1.05934300
H	5.53890500	-3.23288700	2.20568900
H	5.25350200	1.04902500	2.16315600
H	9.11761800	-0.85892900	2.12557600
H	-5.75474600	-3.86247900	-1.37504100
C	-7.55368400	-3.53902500	-0.21903800
H	-5.31587900	-1.43391500	2.13144900
C	-7.31235900	-2.14149500	1.74266700
H	-5.35196500	2.98029600	-2.64317400
C	-7.23370900	1.97590500	-2.96404400
H	-5.16811900	-1.30012000	-2.87291400
C	-7.13300800	-0.44200900	-3.08815600
H	-5.47410200	0.98653200	3.85038300
C	-7.15542000	2.25048600	3.33957600
H	-5.11791000	3.16845000	0.17975400
C	-6.96531400	3.46091100	1.25125100
C	-8.11960500	-2.89936400	0.89255300
C	-8.38210400	-4.40916000	-1.09454400
C	-7.90375900	-1.44931300	2.92422900
C	-7.87775000	0.74069000	-3.10184400
C	-7.97905100	3.26952700	-3.03099100
C	-7.82881300	-1.75612100	-3.17954800
C	-7.69340400	3.13578000	2.39780900
C	-7.91290700	1.92287600	4.57603900
C	-7.57285800	4.37620200	0.24481200
H	-9.17725400	-2.99606100	1.10673900
O	-9.59313700	-4.67221900	-0.58855700
O	-8.01651600	-4.87256400	-2.17135600
O	-6.97281000	-1.16788700	3.84772600
O	-9.08480200	-1.16657200	3.03370600
H	-8.95077300	0.66725300	-3.25093600
O	-9.28527300	3.26421600	-2.69744300

O	-7.45846200	4.32109400	-3.35916000
O	-6.98854400	-2.75348000	-3.49498800
O	-9.01896500	-1.91037500	-2.96007300
H	-8.67388600	3.57207400	2.54529700
O	-8.90103100	2.78686400	4.83441400
O	-7.67800700	0.97094400	5.31546700
O	-6.68373300	4.75781700	-0.69397500
O	-8.73674600	4.73177900	0.25272200
H	0.03647500	4.97085300	2.05084000
H	-0.40360400	-4.61168100	3.41704300
H	-0.42454100	-5.50513100	-1.85520400
H	0.08338600	-0.94671600	-5.61681500
H	1.19373700	-2.33284900	-2.45061200
H	1.00621800	-1.38694500	2.97247500
H	1.27502700	2.92334400	-0.62196500
H	7.61370000	0.83697700	-2.67705300
H	9.10213400	-6.07327900	-0.23564900
H	8.27455000	-5.23156200	2.39163100
H	9.20626400	1.11942300	0.81317300
H	7.41625400	4.16163300	2.50439100
H	8.83347700	4.24774600	-4.57259700
H	-9.55633200	2.41756600	-2.30241700
H	-7.15938400	5.22073500	-1.41304800
H	-9.36431000	2.48051700	5.63860300
H	-7.33748700	-0.53015500	4.51366300
H	-10.07170100	-5.22489000	-1.23679000
H	-7.42640700	-3.61520200	-3.28330800
H	-0.30523800	0.88720100	5.39340200
O	-1.16627400	3.60833000	-1.87177800
H	-1.97253700	3.66866900	-2.41642700
H	-1.12488400	4.41770900	-1.32918000
O	1.65729800	2.24137000	-3.32813800
H	2.49307100	2.73951600	-3.27289100
H	1.56395400	1.93346500	-4.24903100

Sum of electronic and thermal Free Energies = -6767.389391 (Hartree/Particle).

Supplementary Table 11. DFT-optimized geometry of f-[Zr^{III}($BTC^{\bullet-}$)(BTC)(H_2O)$_2$]$^{2-}$ (singlet), computed at the B3LYP-D3/ LANL2DZ (Zr)/ 6-31G* (HCO) level in water solvent using PCM.

Atom	x	y	z
O	8.05426700	1.84575800	-2.36036200
O	6.73630700	4.35145100	-3.38382600
O	8.31135600	5.53097300	-2.28360100
O	9.43568200	0.72666100	-1.05621900
C	8.32571200	0.73795200	-1.61680400
C	7.25276400	4.69906600	-2.33493200
C	7.37865000	-0.34784800	-1.54313700
C	6.79580100	4.25220400	-0.99190200
C	6.05503200	-0.22231600	-1.92253900
C	7.91155100	-1.65767300	-0.98840900
C	5.65627700	3.44993300	-0.92999000
C	7.47184800	4.57994500	0.18484900

H	5.62055700	0.74787500	-2.15541100
C	5.19722700	-1.34776800	-1.98419200
H	8.95939600	-1.74260900	-1.28201200
C	7.12838600	-2.79624200	-1.61806600
H	5.12499100	3.21472600	-1.84312700
C	5.19710400	2.94924900	0.29063400
H	8.33901700	5.22890100	0.16154300
C	7.04716800	4.05176600	1.40885000
C	5.81557300	-2.63357200	-1.94475900
C	3.77511600	-1.23172200	-2.11583700
C	7.71362200	-4.11848000	-1.81329200
C	5.91255000	3.22998500	1.46314400
C	3.92956300	2.14325000	0.29125800
C	7.81275600	4.47222900	2.63646200
H	5.23483700	-3.49746400	-2.25256800
O	3.03390900	-2.26233400	-1.84016800
O	3.24315000	-0.10633900	-2.48513500
O	9.06303900	-4.14187900	-1.60735800
O	7.12072600	-5.14508400	-2.14288100
H	5.57447000	2.81519900	2.40469800
O	3.37610100	2.00971400	-0.84869200
O	3.47882700	1.68015600	1.37741200
O	7.99529500	3.57215400	3.59366200
O	8.24483300	5.61110100	2.74121100
Zr	1.35306500	-2.37096500	-0.42754600
Zr	1.55893100	0.97484200	-1.63889200
Zr	1.60223300	0.38396200	1.87661700
Zr	-1.52843900	-0.77589100	-1.86384700
Zr	-1.50104800	-1.42232600	1.49403900
O	3.07397000	-2.78290300	0.93944800
O	0.71636100	-1.02821100	-2.19776700
O	-0.67269800	-1.92703100	-0.34973200
O	0.75122800	-1.74918600	1.68421700
O	1.84583300	-0.33125900	-0.06633300
O	0.66716400	-4.25928700	0.61302200
O	0.59382600	-3.75064000	-2.08224700
Zr	-1.33371500	1.73257900	0.37043300
O	-0.52242800	1.03810500	-1.42282200
O	0.94215300	2.06134300	0.27459200
O	0.96985400	1.17742500	-3.81637500
O	7.54997500	-5.39319400	1.52110400
O	3.23368500	-0.94251600	2.24839900
O	-0.46404200	0.40192100	1.65871600
O	1.21824500	2.27052900	3.02267100
O	0.98379900	-0.30965200	3.95583200
O	7.48323900	1.15705100	2.95341000
O	9.18993200	0.85115700	1.57026700
O	9.44204700	-4.17380000	1.43317000
O	-3.48373400	-1.99311900	-1.53857600
O	-3.12655800	0.64466100	-2.47910600
O	-2.29805600	-0.07858600	0.00463900
O	-0.96525400	0.00027400	-4.02776700
O	-1.23430100	-2.73316400	-2.98418900
O	-3.28750900	-0.36087700	2.53849500
O	-3.25105600	-2.51428600	0.64442500
O	-1.12580400	-3.66630300	1.88338600
O	-0.99965900	-1.40685500	3.72794600
C	3.78453800	-1.90260800	1.55878900
C	-0.29228700	-4.48658600	1.40955100
C	-0.40021200	-3.67680400	-2.86106700

O	-3.17818300	2.34759700	-1.00050800
O	-2.99104100	1.76265800	1.83539900
O	-0.67645700	3.18262800	2.16023400
C	-0.04027400	0.73221200	-4.45490900
C	8.21469600	-4.20295900	1.35927500
C	0.27715300	3.12171900	2.98038600
C	-0.04001500	-0.91705700	4.38762700
C	7.97054400	0.49570700	2.00554100
C	-3.93658100	-2.35056100	-0.41275100
C	-3.70257600	1.60298000	-1.88013600
C	-3.69425400	0.81490600	2.31276500
C	5.21929800	-1.93473400	1.48120900
C	7.34845800	-3.05997000	1.13457600
C	7.28119100	-0.63760900	1.43168300
C	-5.41064600	-2.59044300	-0.24575000
C	-5.13885100	1.80648100	-2.26839900
C	-5.12495700	1.17329600	2.58478800
C	5.98729200	-3.12552600	1.31653000
C	5.93989700	-0.74357300	1.72524100
C	7.96393900	-1.75617500	0.66016600
C	-6.25698600	-2.98997700	-1.28263600
C	-5.93029500	-2.39934800	1.03926100
C	-5.79558000	3.03631000	-2.20071100
C	-5.82244700	0.67674300	-2.72943400
C	-5.90095800	0.56269000	3.57166700
C	-5.67842800	2.18341400	1.79117800
H	5.49959300	-4.08382100	1.47097600
H	5.41875300	0.09801100	2.16548600
H	9.02819700	-1.76436900	0.90784100
H	-5.86065100	-3.14801900	-2.27858900
C	-7.62088200	-3.21457700	-1.02240100
H	-5.26539300	-2.08318500	1.83277000
C	-7.29136200	-2.57590200	1.28911900
H	-5.27177300	3.91542000	-1.84384100
C	-7.12873700	3.13698300	-2.63684400
H	-5.30341900	-0.27231500	-2.76644500
C	-7.16350000	0.76016100	-3.09946300
H	-5.47635000	-0.21603200	4.19369900
C	-7.22765100	0.98140300	3.77320600
H	-5.06864400	2.64328600	1.02401700
C	-7.00784900	2.57159900	1.96045000
C	-8.14016300	-2.98252800	0.25838500
C	-8.49851400	-3.72599900	-2.10763600
C	-7.84480400	-2.29744900	2.64445300
C	-7.81911900	1.99276100	-3.05803700
C	-7.79529700	4.47351800	-2.64820500
C	-7.90128000	-0.47088700	-3.50377900
C	-7.78500600	1.97172500	2.95330400
C	-8.02086700	0.37138700	4.87221700
C	-7.60504500	3.60766900	1.06825600
H	-9.19486800	-3.12458800	0.46101300
O	-9.69051100	-4.14881800	-1.66977500
O	-8.18930300	-3.78762400	-3.29480100
O	-6.90520300	-2.38648300	3.59886600
O	-9.00910800	-2.00398500	2.85880500
H	-8.86799100	2.03291000	-3.33708500
O	-8.69944100	4.70875700	-3.60964900
O	-7.56574700	5.36479900	-1.84069200
O	-7.06691500	-1.43272500	-3.92464600
O	-9.11284300	-0.59144700	-3.44170200

H	-8.81450200	2.28291900	3.08424200
O	-9.15067300	1.03525300	5.14376300
O	-7.69304200	-0.63241900	5.49923700
O	-6.66444400	4.34302700	0.46090700
O	-8.80451200	3.75791800	0.90538100
H	0.31336000	3.90602600	3.75363800
H	-0.40313000	-5.53365200	1.73452100
H	-0.55462700	-4.54305800	-3.52428900
H	-0.09103700	1.02810800	-5.51527300
H	1.05649300	-1.36162300	-3.04179200
H	1.10821000	-2.35460100	2.35116500
H	1.30477400	2.94236900	0.45369000
H	7.23032600	1.74433900	-2.86549800
H	9.33883500	-5.06926600	-1.72660100
H	8.24328200	-6.05677300	1.69043000
H	9.34158600	0.55547500	0.63161600
H	7.82264000	2.61241800	3.28342300
H	8.54922900	5.75624300	-3.20337100
H	-8.70159700	4.00283300	-4.28021900
H	-7.07046100	4.86607200	-0.28125200
H	-9.61742100	0.55127100	5.85288900
H	-7.25022500	-1.96905900	4.42797400
H	-10.20524600	-4.44472200	-2.44589300
H	-7.54813700	-2.29837000	-3.94747400
H	-0.09488900	-1.03964900	5.48145600
O	-1.36127600	4.02277100	-0.21635300
H	-2.22288100	4.24241100	-0.61712100
H	-1.26337900	4.55381300	0.59626400
O	1.56285900	3.27767800	-2.32065100
H	2.43077700	3.67478900	-2.12438900
H	1.45362900	3.29748900	-3.28957700

Sum of electronic and thermal Free Energies = -6767.491669 (Hartree/Particle).

Supplementary Table 12. DFT-optimized geometry of f-[Zr^{IV}($BTC^{\cdot-}$)(BTC)(H_2O)]$^-$ (doublet), computed at the UB3LYP-D3/ LANL2DZ (Zr)/ 6-31G* (HCO) level in water solvent using PCM. The spin density plot is also shown below. (Isovalue = 0.0004)

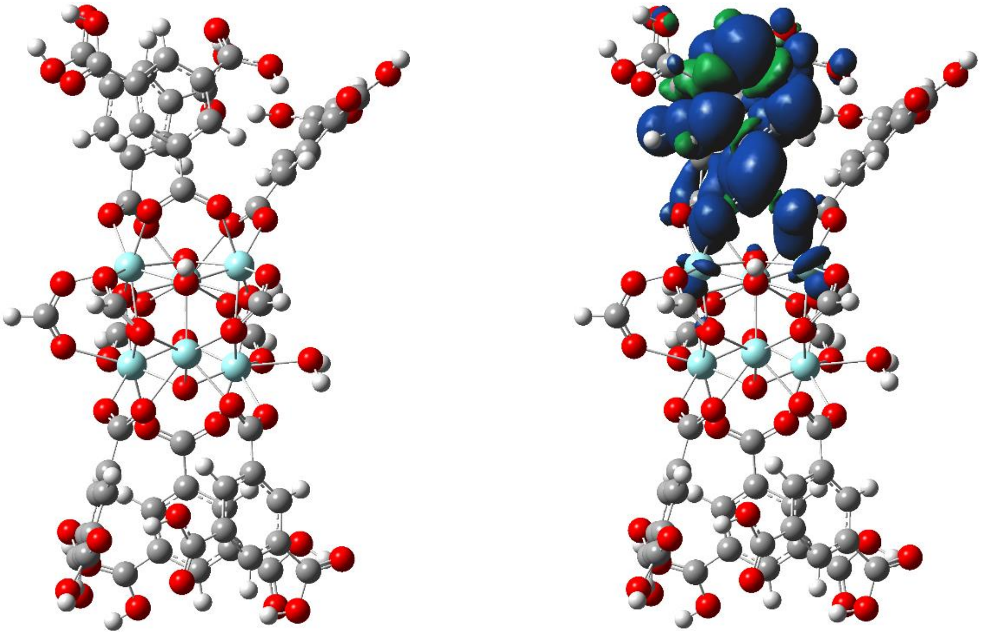

Spin Density Distribution

Atom	x	y	z
O	8.28573100	0.95357800	-2.01553900
O	7.32462300	2.46322200	-4.25562000
O	8.43713100	4.38805900	-3.87566800
O	9.41053300	-0.05157400	-0.40793800
C	8.48675400	-0.11433200	-1.22579500
C	7.55308400	3.43221200	-3.54654200
C	7.58643000	-1.27751000	-1.31369600
C	6.89820300	3.66166200	-2.23128500
C	6.23643200	-1.09740300	-1.59931500
C	8.08506500	-2.54426200	-0.90242100
C	5.76553100	2.90331900	-1.93264400
C	7.39609300	4.56275200	-1.28772100
H	5.84379500	-0.12133100	-1.86858200
C	5.30568100	-2.14786800	-1.38257400
H	9.13730400	-2.66952600	-0.69000100
C	7.17394100	-3.61239600	-0.77193700
H	5.35982600	2.23186800	-2.67897600
C	5.14402300	3.00783800	-0.68615300
H	8.25110200	5.18747900	-1.51784600
C	6.81165800	4.63744500	-0.01886300
C	5.81251900	-3.40825700	-0.95139600
C	3.88966300	-1.93870200	-1.49564900
C	7.61409900	-4.98799700	-0.42865900
C	5.69262100	3.85132000	0.28900500
C	3.88861100	2.21914300	-0.45633700
C	7.38714700	5.63424700	0.94924300
H	5.12508500	-4.22993300	-0.79437100
O	3.09022900	-2.77857200	-0.89563000
O	3.42395100	-0.92862900	-2.18021800
O	8.95479800	-5.08651900	-0.24875400
O	6.88178100	-5.95908200	-0.32544800
H	5.22889700	3.90604400	1.26735100
O	3.40454300	1.66395400	-1.49406800
O	3.38884000	2.15969400	0.70627200

O	7.47258600	5.26876500	2.23010800
O	7.75417800	6.73687800	0.58220300
Zr	1.38854400	-2.35560300	0.36954500
Zr	1.68714300	0.32195900	-1.94494900
Zr	1.47624700	1.09298200	1.51035000
Zr	-1.41669400	-1.42784700	-1.67701000
Zr	-1.54816800	-0.81731900	1.68033100
O	3.18287700	-2.10576800	1.77533100
O	0.85424300	-1.75992900	-1.78705900
O	-0.63107700	-1.94212400	0.19192300
O	0.71275400	-0.99790000	2.08572400
O	1.85777200	-0.30164400	0.00538500
O	0.73466600	-3.70583200	2.03637500
O	0.68423100	-4.25373600	-0.66570700
Zr	-1.35300100	1.72871200	-0.51909000
O	-0.41295400	0.41090400	-1.83768700
O	0.94489100	2.02249700	-0.59266100
O	1.16073500	-0.25582000	-4.03340400
O	7.62974800	-4.39421800	2.48702400
O	3.15883400	0.05003100	2.46474200
O	-0.55278500	0.97910800	1.22563900
O	0.97487700	3.27829700	1.84562000
O	0.81774400	1.18151400	3.66425500
O	7.25582800	2.65675400	2.59676900
O	8.96100100	1.94587300	1.36567100
O	9.53077000	-3.18451400	2.41792400
O	-3.39115000	-2.49560700	-0.98760700
O	-3.00254300	-0.32908200	-2.82561000
O	-2.28083200	-0.12469700	-0.22471400
O	-0.77389300	-1.45063800	-3.91164100
O	-1.06687300	-3.66847500	-1.99862100
O	-3.37280500	0.51420700	2.23396300
O	-3.24822700	-2.15969900	1.23834100
O	-1.15952400	-2.76168000	2.87205000
O	-1.14103500	0.02342000	3.79277900
C	3.75769900	-1.03657000	2.14217300
C	-0.27821800	-3.65901400	2.80235500
C	-0.26026500	-4.48908700	-1.47481200
O	-3.04721800	1.79135900	-2.06786900
O	-3.09928900	2.22214900	0.78135000
O	-0.83530600	3.73570500	0.54904500
C	0.17048300	-0.89233900	-4.52468300
C	8.31394200	-3.23342700	2.38795300
C	0.04364600	4.01926400	1.41035000
C	-0.22043900	0.75130500	4.25567700
C	7.82963400	1.72741800	2.02570100
C	-3.89007300	-2.41845700	0.16994500
C	-3.59865500	0.75587600	-2.55873900
C	-3.76926600	1.55348000	1.62871400
C	5.24455300	-0.99529600	2.18915300
C	7.42469100	-2.04885600	2.25761000
C	7.25511700	0.35836300	2.07834400
C	-5.36525900	-2.61615500	0.37278700
C	-5.07332700	0.78309700	-2.80770600
C	-5.14693100	2.08913600	1.90966000
C	6.02837700	-2.15890700	2.24082300
C	5.86123100	0.25493400	2.13494500
C	8.03987600	-0.79712500	2.14853300
C	-6.17186400	-3.39616000	-0.45824000
C	-5.92728000	-1.98024000	1.48481200

C	-5.80523400	1.97064200	-2.85227800
C	-5.72886700	-0.44558200	-2.93544000
C	-5.86261800	1.76337800	3.06483900
C	-5.70339400	2.97097800	0.97582600
H	5.54955900	-3.12863600	2.27999800
H	5.25082200	1.14832200	2.09441300
H	9.12116300	-0.74676700	2.13695500
H	-5.74475700	-3.89962400	-1.31739900
C	-7.53762900	-3.54951300	-0.15956500
H	-5.29317100	-1.37394600	2.11829000
C	-7.28882500	-2.09993400	1.76283600
H	-5.30512000	2.92752200	-2.75668300
C	-7.19950800	1.92707400	-3.00360700
H	-5.15092800	-1.35865500	-2.88254000
C	-7.11562900	-0.49345900	-3.07306800
H	-5.43649700	1.07872500	3.78794200
C	-7.11942900	2.34811200	3.29680200
H	-5.14301400	3.20916900	0.08159700
C	-6.96567700	3.52895500	1.18852800
C	-8.09855900	-2.88364900	0.93898000
C	-8.36991000	-4.44163900	-1.00864700
C	-7.87447400	-1.38138400	2.93169900
C	-7.85438300	0.69325000	-3.09395700
C	-7.93719700	3.22405100	-3.08713800
C	-7.82050900	-1.80512100	-3.12220000
C	-7.66975000	3.22651100	2.35615000
C	-7.85447800	2.04085800	4.55188700
C	-7.59014900	4.44308500	0.19102300
H	-9.15429300	-2.97909200	1.16302200
O	-9.56921800	-4.71110600	-0.47907500
O	-8.01656800	-4.91606800	-2.08484800
O	-6.93646700	-1.06771500	3.83719600
O	-9.05700100	-1.10722600	3.04658500
H	-8.93154300	0.62541000	-3.21273900
O	-9.21952000	3.24601300	-2.66725900
O	-7.43276100	4.25197600	-3.49991900
O	-6.99332100	-2.81290100	-3.43847700
O	-9.00607900	-1.94822700	-2.87221500
H	-8.64219200	3.67476500	2.51991200
O	-8.81928000	2.92520900	4.82849600
O	-7.62184200	1.08734200	5.28996300
O	-6.73612600	4.77163800	-0.79921800
O	-8.73755200	4.84412200	0.24915000
H	0.00776800	5.03765900	1.82804100
H	-0.38726100	-4.50767600	3.49567700
H	-0.38808000	-5.54379000	-1.76670200
H	0.15769100	-0.96533300	-5.62339500
H	1.23105900	-2.38140700	-2.42888600
H	1.00638000	-1.28660000	2.96274800
H	1.30042000	2.91472900	-0.72610300
H	7.63341700	0.79540000	-2.72345700
H	9.14546100	-6.02447100	-0.05804400
H	8.28850500	-5.11201300	2.54387000
H	9.21095200	1.19168400	0.76333100
H	7.39988000	4.27768400	2.34248800
H	8.84083800	4.13867000	-4.72959900
H	-9.46791900	2.41999100	-2.21734900
H	-7.22450100	5.26125300	-1.49049600
H	-9.27077300	2.63120500	5.64395600
H	-7.29872300	-0.41865500	4.49367700

H	-10.05260300	-5.27762300	-1.11159800
H	-7.43209500	-3.66889900	-3.20496000
H	-0.31906500	1.04700300	5.31215800
O	-0.96888400	3.45469900	-2.15423900
H	-1.74074300	3.47359000	-2.74968300
H	-0.94162000	4.32224400	-1.71021500

Sum of electronic and thermal Free Energies = -6690.968917 (Hartree/Particle).

Supplementary Table 13. DFT-optimized geometry of f-[Zr^{III}($BTC^{\cdot -}$)(BTC)(H_2O)]$^{2-}$ (singlet), computed at the B3LYP-D3/ LANL2DZ (Zr)/ 6-31G* (HCO) level in water solvent using PCM.

Atom	x	y	z
O	8.13800700	1.30530300	-2.58414700
O	6.84976100	3.47494300	-4.24007400
O	8.36338300	4.92248500	-3.40558700
O	9.48164300	0.52909900	-1.01802700
C	8.38877900	0.40147400	-1.59740900
C	7.31937500	4.07857900	-3.28999500
C	7.44191700	-0.64642900	-1.30140100
C	6.81680900	3.95905300	-1.89516600
C	6.13167800	-0.62690100	-1.74157600
C	7.95837700	-1.78522600	-0.43920900
C	5.67378900	3.18806500	-1.68223000
C	7.45668900	4.55748500	-0.80807900
H	5.70252400	0.25785300	-2.20782500
C	5.27927600	-1.74502000	-1.56634300
H	9.01664300	-1.92312600	-0.66713100
C	7.20198000	-3.04715400	-0.81362800
H	5.16542300	2.74562400	-2.52919200
C	5.17878600	2.98383900	-0.39184500
H	8.32495900	5.18800900	-0.95715500
C	6.99469800	4.32821600	0.49257200
C	5.89921700	-2.97981500	-1.20795900
C	3.86402900	-1.67580100	-1.77624900
C	7.80157900	-4.37084100	-0.68410400
C	5.85915500	3.53374500	0.70396800
C	3.91447300	2.19008900	-0.24021100
C	7.72064100	5.03238600	1.60936800
H	5.33288000	-3.89839200	-1.32648700
O	3.10778400	-2.61318600	-1.28996200
O	3.35138600	-0.67520900	-2.42820300
O	9.14498200	-4.32781900	-0.44639100
O	7.22499100	-5.45346700	-0.78186500
H	5.49317800	3.34709100	1.70613000
O	3.39445400	1.78550600	-1.33154200
O	3.43452100	1.98482600	0.91301700
O	7.87579900	4.38446000	2.75651600
O	8.14565900	6.16839800	1.45620900
Zr	1.37601000	-2.40389100	0.04682400
Zr	1.63863200	0.55834100	-1.90802800
Zr	1.54475000	0.84529800	1.62813500
Zr	-1.45114300	-1.18808700	-1.82662100

Zr	-1.53366900	-1.01681700	1.57985800
O	3.04479700	-2.45683700	1.53702300
O	0.80916400	-1.51481000	-2.02194200
O	-0.64531800	-1.94792500	-0.05879400
O	0.70250500	-1.27845200	1.92446800
O	1.86006400	-0.33081900	-0.07629500
O	0.62304300	-3.97234800	1.48995700
O	0.68254100	-4.12896700	-1.25555000
Zr	-1.33215600	1.79248700	-0.25364900
O	-0.44703200	0.66278400	-1.77029900
O	0.95753000	2.07779900	-0.35371700
O	1.07204100	0.32330500	-4.05592900
O	7.47851800	-4.83743800	2.85394700
O	3.16745700	-0.34716700	2.35029400
O	-0.50739900	0.80626600	1.34447300
O	1.09488000	2.97806300	2.23907600
O	0.85163100	0.67938200	3.78456800
O	7.38716300	1.88127300	2.69299200
O	9.14817000	1.25872600	1.49673100
O	9.38406700	-3.68135300	2.52740500
O	-3.40351700	-2.31652300	-1.28580500
O	-3.04022600	0.07253200	-2.79342900
O	-2.28298000	-0.07483200	-0.20393100
O	-0.83785600	-0.92019900	-4.07677700
O	-1.10997600	-3.37028300	-2.43950200
O	-3.34683700	0.26429900	2.30330800
O	-3.25230500	-2.29204100	0.96517200
O	-1.20303700	-3.09154200	2.52387800
O	-1.11826300	-0.46788800	3.77342800
C	3.73693300	-1.44624500	1.94279500
C	-0.36100400	-4.00129900	2.28865900
C	-0.28331800	-4.24991600	-2.06616600
O	-3.08554700	2.06443100	-1.74403000
O	-3.03953600	2.15770500	1.11877500
O	-0.75011600	3.63365300	1.08256800
C	0.08238700	-0.25578300	-4.61636200
C	8.15816300	-3.72026000	2.43520900
C	0.16218600	3.78308100	1.94188800
C	-0.18474800	0.18289300	4.32048900
C	7.91049200	1.01230600	1.95533100
C	-3.89656200	-2.40295900	-0.12394500
C	-3.63609300	1.10787000	-2.37260500
C	-3.72871900	1.38090300	1.85215600
C	5.17320500	-1.48981900	1.92825500
C	7.30788000	-2.65723600	1.93029900
C	7.24108700	-0.23140400	1.64773100
C	-5.37001300	-2.63451700	0.05107000
C	-5.11350900	1.16106300	-2.61473400
C	-5.10754500	1.88006300	2.18495200
C	5.94096300	-2.68272300	2.07571300
C	5.88951100	-0.27169200	1.90744700
C	7.94758300	-1.49713800	1.18835600
C	-6.17099400	-3.31218700	-0.86977700
C	-5.93559200	-2.14455200	1.23270100
C	-5.85518500	2.33894400	-2.51113400
C	-5.75917900	-0.04532700	-2.90510900
C	-5.84221800	1.40594100	3.27510300
C	-5.64889100	2.87598200	1.36469300
H	5.44336200	-3.58065100	2.43044900
H	5.35543800	0.64811200	2.11338700

H	9.00163400	-1.44233300	1.47156300
H	-5.74103500	-3.70068200	-1.78533500
C	-7.53441600	-3.51491300	-0.59067600
H	-5.30555900	-1.61393800	1.93456900
C	-7.29525600	-2.31081800	1.49556700
H	-5.36419200	3.27910400	-2.28777000
C	-7.24847100	2.30582200	-2.68228700
H	-5.17417800	-0.95335700	-2.96634600
C	-7.14405700	-0.08515800	-3.06162500
H	-5.42876600	0.63063200	3.90871300
C	-7.10285900	1.95786400	3.56068000
H	-5.07416500	3.22563000	0.51745400
C	-6.91675400	3.39971500	1.62435800
C	-8.09891400	-2.99706800	0.58314900
C	-8.35985300	-4.29948900	-1.54585300
C	-7.88510900	-1.74400400	2.74317100
C	-7.89165500	1.08941400	-2.93942300
C	-7.99942700	3.59604300	-2.61112800
C	-7.83793600	-1.38416700	-3.28769900
C	-7.63999100	2.94922400	2.73089000
C	-7.85724100	1.49323500	4.75455900
C	-7.52967400	4.42089900	0.72960000
H	-9.15294400	-3.13112200	0.79529100
O	-9.54629900	-4.66561100	-1.04625000
O	-8.01164900	-4.61222700	-2.68126800
O	-6.94475500	-1.51217600	3.67065400
O	-9.07231900	-1.51308200	2.89786900
H	-8.96638100	1.02932500	-3.08179200
O	-9.29824300	3.55046200	-2.25085600
O	-7.49128900	4.67633300	-2.85311800
O	-7.00138400	-2.33498000	-3.73012100
O	-9.02354600	-1.56760500	-3.06570700
H	-8.61732500	3.37031300	2.93320700
O	-8.82662700	2.33673200	5.12695200
O	-7.63579800	0.45307100	5.36847500
O	-6.65240800	4.88965700	-0.18046300
O	-8.68967600	4.78356700	0.79366400
H	0.15983700	4.73526600	2.49615400
H	-0.48806000	-4.93875200	2.85343000
H	-0.41119400	-5.25094300	-2.50853800
H	0.04638000	-0.16321200	-5.71346400
H	1.17740700	-2.03445400	-2.75278000
H	1.03129000	-1.70061800	2.73228600
H	1.33726600	2.96994700	-0.36294400
H	7.33173300	1.08156000	-3.07837600
H	9.43102800	-5.25387000	-0.34176100
H	8.16209500	-5.44769000	3.18554800
H	9.33403200	0.75037700	0.66100600
H	7.71447400	3.37742700	2.67700400
H	8.63422100	4.92347000	-4.34366400
H	-9.55481100	2.66915100	-1.92854600
H	-7.13718900	5.42444600	-0.84104700
H	-9.28981500	1.94212800	5.89180100
H	-7.31262900	-0.95002700	4.39968600
H	-10.02706900	-5.14896300	-1.74631800
H	-7.43370700	-3.21805200	-3.61373900
H	-0.27472500	0.33505500	5.40818600
O	-1.07949400	3.75999900	-1.58591800
H	-1.88905300	3.87285000	-2.11746600
H	-1.00877700	4.54143900	-1.00713400

Sum of electronic and thermal Free Energies = -6691.070660 (Hartree/Particle).

Supplementary Table 14. DFT-optimized geometry of f-[Zr^{V}(BTC)(BTC)(H_2O)(CO_2^{2-})]$^{-}$ (doublet), computed at the UB3LYP-D3/ LANL2DZ (Zr)/ 6-31G* (HCO) level in water solvent using PCM. The spin density plot is also shown below. (Isovalue = 0.0004)

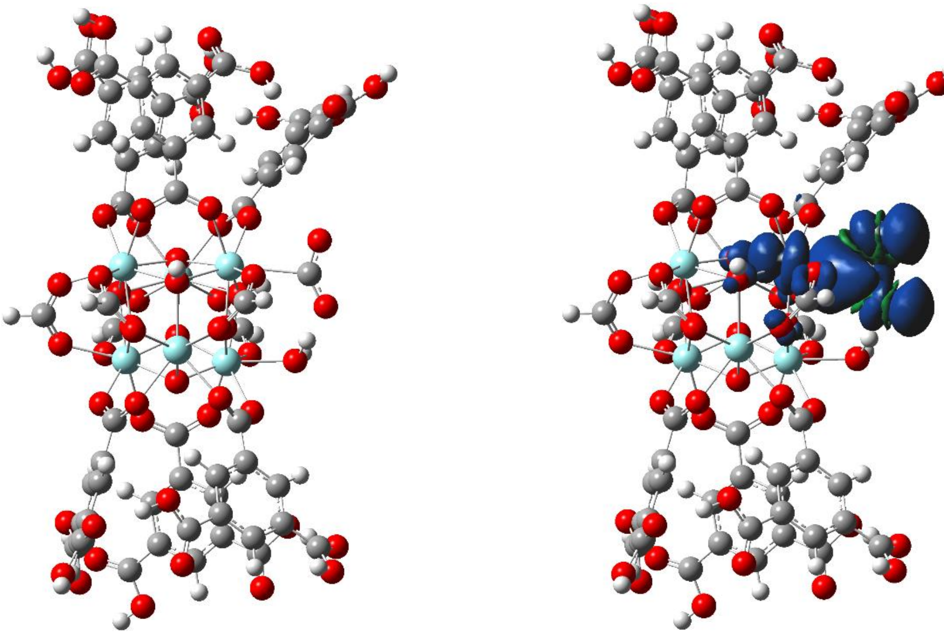

Spin Density Distribution

Atom	x	y	z
O	8.25070600	1.22218700	-1.85856400
O	7.19809100	3.08614400	-3.67160500
O	8.18925300	5.00712000	-3.02685400
O	9.34294700	-0.12660700	-0.50275700
C	8.43726400	0.02025900	-1.31823900
C	7.40095500	3.94247800	-2.82124100
C	7.50916200	-1.09620200	-1.65410400
C	6.81553100	3.89087500	-1.45566700
C	6.13292800	-0.87687500	-1.78049100
C	8.02036700	-2.40024900	-1.67244000
C	5.70046300	3.07366000	-1.26414800
C	7.36498000	4.58013400	-0.37279200
H	5.70950400	0.12157700	-1.73433900
C	5.26344300	-1.97188600	-1.85468100
H	9.08384700	-2.55954300	-1.54371800
C	7.15394000	-3.48508700	-1.83140100
H	5.23970200	2.58278400	-2.11259600
C	5.15164500	2.90659700	0.00902800
H	8.20610500	5.24900200	-0.51257200
C	6.84980400	4.38315700	0.91373400
C	5.77290800	-3.26930800	-1.88257400
C	3.78719300	-1.73849500	-1.77786700
C	7.64067600	-4.89306700	-1.89169900

C	5.74839400	3.53839200	1.10869600
C	3.90999000	2.07494900	0.13168000
C	7.47128200	5.17224100	2.03342100
H	5.10017400	-4.11749600	-1.93157200
O	3.10157600	-2.62124600	-1.17781000
O	3.34149600	-0.66177400	-2.27580100
O	8.97550800	-4.99075200	-1.72839000
O	6.91972300	-5.85736900	-2.06512200
H	5.33240300	3.38871900	2.09864000
O	3.42875700	1.65694500	-0.96529100
O	3.41440400	1.86123400	1.27993400
O	7.58520400	4.57692700	3.22386700
O	7.84644200	6.31936800	1.86761000
Zr	1.31148300	-2.42663900	0.19269900
Zr	1.59219900	0.61869900	-1.65910600
Zr	1.51048600	0.75623300	1.91077300
Zr	-1.50173700	-1.12431700	-1.59960700
Zr	-1.55412000	-1.09307700	1.81800800
O	3.14612000	-2.50383900	1.55229000
O	0.76977400	-1.47176000	-1.81797300
O	-0.68022900	-1.96919300	0.14138300
O	0.71491500	-1.38980600	2.13543700
O	1.82590400	-0.36452500	0.16108600
O	0.60206100	-4.02637800	1.61115800
O	0.62093200	-4.08755000	-1.15700800
Zr	-1.36885400	1.80382100	0.08231400
O	-0.47820400	0.73080700	-1.48824300
O	0.95868700	2.06350300	-0.00402600
O	1.02722600	0.31647100	-3.81529800
O	7.52066400	-4.89112800	1.16062800
O	3.20192900	-0.51654600	2.61901700
O	-0.51633900	0.73085000	1.63654100
O	1.10672300	2.83906300	2.62328300
O	0.87540700	0.48870300	4.06322500
O	7.37695100	1.92065900	3.14486600
O	9.00386900	1.47818000	1.70507100
O	9.45408700	-3.73782500	1.30228300
O	-3.44542900	-2.26454100	-1.08379600
O	-3.05728900	0.14432500	-2.50997000
O	-2.29805600	-0.07943200	0.07552000
O	-0.92730600	-0.85158100	-3.82833900
O	-1.18451500	-3.29158300	-2.28944000
O	-3.36094600	0.16258700	2.58556800
O	-3.27759100	-2.33091200	1.16475200
O	-1.22285300	-3.19454000	2.68647200
O	-1.10259200	-0.64240300	4.02453800
C	3.75659100	-1.52570500	2.07930300
C	-0.37942000	-4.09175900	2.41409500
C	-0.36165900	-4.18649700	-1.95432800
O	-3.17491000	2.08384900	-1.37031100
O	-3.04663100	2.09828800	1.47968400
O	-0.76542500	3.54667900	1.55184600
C	0.02680700	-0.23560100	-4.37383600
C	8.23917100	-3.76677200	1.36459300
C	0.16143100	3.65528200	2.39763800
C	-0.16098400	-0.02052200	4.59037800
C	7.90254600	1.13243500	2.36156600
C	-3.93095000	-2.39976500	0.07667100
C	-3.66548600	1.18629600	-2.10958600
C	-3.76634000	1.26192500	2.11435500

C	5.25115400	-1.51202800	2.03801800
C	7.38872600	-2.57981500	1.65824100
C	7.29316100	-0.20896300	2.13561700
C	-5.40311200	-2.63889400	0.24495900
C	-5.09045300	1.28797800	-2.57115400
C	-5.20704000	1.64831000	2.26127800
C	5.99269600	-2.65581000	1.72470400
C	5.90310200	-0.29750900	2.25702200
C	8.03887900	-1.35423500	1.85073700
C	-6.20630000	-3.25587300	-0.71678400
C	-5.96832800	-2.21116800	1.45097000
C	-5.75737100	2.49747200	-2.77336800
C	-5.75002200	0.07919300	-2.81689400
C	-6.01692400	1.21946700	3.31518100
C	-5.73654600	2.48200500	1.27091300
H	5.48071400	-3.59195100	1.53898200
H	5.32061000	0.58632100	2.48403900
H	9.11834200	-1.32280600	1.77406800
H	-5.77383300	-3.59732300	-1.64965300
C	-7.57345400	-3.45840300	-0.45662600
H	-5.33674500	-1.72837700	2.18541800
C	-7.33304900	-2.36880400	1.69161800
H	-5.25036800	3.43732600	-2.58905900
C	-7.07738100	2.49030100	-3.25858700
H	-5.22217300	-0.85093300	-2.64999800
C	-7.07951400	0.06835000	-3.23510800
H	-5.61087900	0.57796000	4.08811300
C	-7.35457600	1.64567800	3.38338700
H	-5.09974800	2.80112000	0.45555800
C	-7.07682000	2.86792500	1.31133300
C	-8.13930600	-2.99253400	0.73793300
C	-8.40491100	-4.19115300	-1.44744300
C	-7.93327100	-1.83682200	2.94767700
C	-7.74568300	1.27522500	-3.45903500
C	-7.75160600	3.78795800	-3.56252600
C	-7.79411600	-1.22822900	-3.41342700
C	-7.88811200	2.45110900	2.36821000
C	-8.18497800	1.24853200	4.55069300
C	-7.64998700	3.70007400	0.21383700
H	-9.19740200	-3.11820700	0.93391300
O	-9.59540600	-4.56654900	-0.96530500
O	-8.06095300	-4.45845300	-2.59584300
O	-7.01911100	-1.70224900	3.92152800
O	-9.10932200	-1.53812700	3.06939700
H	-8.78555700	1.24409700	-3.77099200
O	-8.65065400	3.80459200	-4.55683600
O	-7.53273300	4.83455400	-2.96587400
O	-6.93898200	-2.24157100	-3.61374700
O	-9.00607200	-1.35127400	-3.36663400
H	-8.92587900	2.76097700	2.39664000
O	-9.33786600	1.92271600	4.63553600
O	-7.86695000	0.40308600	5.38263300
O	-6.69645900	4.33851400	-0.47671500
O	-8.84256100	3.77715900	-0.03049400
H	0.16785300	4.57160400	3.00807000
H	-0.49793800	-5.05019300	2.94267800
H	-0.50160700	-5.17623000	-2.41588300
H	0.00542400	-0.17242300	-5.47289600
H	1.06994800	-1.96779900	-2.59472800
H	1.01686900	-1.83735600	2.94034500

H	1.35046900	2.94964700	0.02783300
H	7.59333400	1.26103200	-2.58232000
H	9.21282900	-5.93559300	-1.79673800
H	8.15394200	-5.61120400	0.97641400
H	9.22459300	0.86591600	0.95727300
H	7.50506700	3.58514200	3.16208800
H	8.56052700	4.93588800	-3.92798800
H	-8.64712800	2.97075600	-5.05940600
H	-7.07634900	4.69204400	-1.32523800
H	-9.82581600	1.58167100	5.41046000
H	-7.39367200	-1.12955500	4.63677600
H	-10.08075000	-5.01484900	-1.68535600
H	-7.41273000	-3.10249200	-3.48641000
H	-0.23907100	0.08860500	5.68330600
O	-1.15553800	3.82166300	-1.03652000
H	-0.75733900	4.46804500	-0.42726500
H	-0.54564600	3.75994100	-1.83494000
C	1.53683100	2.98459800	-2.99101900
O	0.51339900	3.68451300	-3.04028300
O	2.63118600	2.95712800	-3.53814600

Sum of electronic and thermal Free Energies = -6879.548477 (Hartree/Particle).

Supplementary Table 15. DFT-optimized geometry of **f-[Zr^{IV}(BTC)(BTC)(H_2O)(CO_2^{2-})]$^{2-}$** (singlet), computed at the B3LYP-D3/ LANL2DZ (Zr)/ 6-31G* (HCO) level in water solvent using PCM.

Atom	x	y	z
O	8.24069500	1.17449300	-1.86735100
O	7.20941600	2.99473300	-3.75217500
O	8.20688400	4.93031100	-3.16511300
O	9.34618900	-0.10353500	-0.45599500
C	8.43741600	-0.00207400	-1.27574400
C	7.41344900	3.87473900	-2.92664000
C	7.52280100	-1.14076100	-1.56655000
C	6.82728100	3.86812700	-1.56109100
C	6.14631500	-0.94308000	-1.72946500
C	8.04945300	-2.43786900	-1.51867500
C	5.72875900	3.03657400	-1.33632300
C	7.36208400	4.61086800	-0.50636800
H	5.70747700	0.05041200	-1.73490300
C	5.29036700	-2.05007500	-1.77512300
H	9.11241500	-2.57829500	-1.36651700
C	7.19769800	-3.53847200	-1.64297500
H	5.28046000	2.49601700	-2.16109200
C	5.17672500	2.91132400	-0.06038200
H	8.19360000	5.28615400	-0.67050400
C	6.84456700	4.45827800	0.78504200
C	5.81531700	-3.34041700	-1.73064300
C	3.80518900	-1.82911600	-1.76963400
C	7.70132500	-4.94077600	-1.63123700
C	5.75450600	3.60509500	1.01070800
C	3.95159300	2.04753200	0.08591700
C	7.45433600	5.29404600	1.87569200

H	5.15270300	-4.19774900	-1.75473500
O	3.12083300	-2.66456600	-1.09962200
O	3.37185300	-0.82483500	-2.40163400
O	9.03741800	-5.01486600	-1.46126600
O	6.99397700	-5.92294900	-1.75709500
H	5.33612600	3.49122300	2.00447600
O	3.56717200	1.46968100	-0.96294800
O	3.38823200	1.98168500	1.22688500
O	7.57218400	4.74164400	3.08720100
O	7.81991000	6.43855700	1.67239500
Zr	1.32304100	-2.35702400	0.26368400
Zr	1.62755500	0.56192200	-1.85630100
Zr	1.50018700	0.85167900	1.78353700
Zr	-1.51254300	-1.24719600	-1.56276300
Zr	-1.55281300	-0.98651900	1.83522900
O	3.16008400	-2.40761800	1.65305600
O	0.75307500	-1.58442500	-1.78764600
O	-0.68741600	-1.98334900	0.22365400
O	0.71162600	-1.25703600	2.16875300
O	1.83662300	-0.35673700	0.13278300
O	0.66078700	-3.91999700	1.80311600
O	0.69146200	-4.16574600	-0.96053000
Zr	-1.34760500	1.76563000	-0.12825500
O	-0.52878300	0.58875300	-1.60101700
O	0.97971800	1.99237100	-0.14167700
O	0.89897100	-0.11401900	-4.01143500
O	7.52957800	-4.80657100	1.42404400
O	3.19925200	-0.36801500	2.62327500
O	-0.53937700	0.82019800	1.54136700
O	1.04612900	2.99519800	2.41546600
O	0.88812300	0.71305500	3.98406600
O	7.38876100	2.08002100	3.11080800
O	9.01207000	1.58365100	1.68391600
O	9.46400000	-3.65063800	1.52041200
O	-3.49599400	-2.36763400	-0.95306400
O	-3.14434700	-0.06561100	-2.53584000
O	-2.32066100	-0.10504400	0.04347400
O	-1.07373000	-1.22691200	-3.79153700
O	-1.19241400	-3.49983500	-2.05417600
O	-3.34598400	0.30288900	2.54718400
O	-3.29000100	-2.29118900	1.29280900
O	-1.18303400	-3.05562500	2.82529900
O	-1.10487200	-0.39332800	4.01869900
C	3.75828500	-1.40100100	2.13767800
C	-0.32133500	-3.95091900	2.60384800
C	-0.32855300	-4.34412000	-1.69487300
O	-3.20845100	1.95948500	-1.53722800
O	-3.03465200	2.16175200	1.30465100
O	-0.75772100	3.64152400	1.19015300
C	-0.11256300	-0.72171400	-4.44692400
C	8.24832500	-3.67344300	1.57501800
C	0.12935000	3.79933600	2.07388800
C	-0.14918200	0.24618000	4.54209900
C	7.91098400	1.26369400	2.35453600
C	-3.96093100	-2.41293800	0.21986000
C	-3.72289700	1.01148700	-2.19490100
C	-3.74108300	1.37857900	2.00823800
C	5.25635400	-1.38722000	2.11064200
C	7.39793600	-2.47272400	1.80333900
C	7.29997600	-0.08381200	2.17602400

C	-5.43901000	-2.60161500	0.43071300
C	-5.16257900	1.10090500	-2.62402300
C	-5.17614600	1.79385600	2.16723400
C	6.00087200	-2.54341900	1.85750400
C	5.90806300	-0.16562300	2.28460200
C	8.04775000	-1.24054600	1.94823700
C	-6.27442900	-3.27265600	-0.46484200
C	-5.97269400	-2.07146800	1.61040700
C	-5.82280300	2.30105000	-2.89322600
C	-5.84225800	-0.11275800	-2.76908100
C	-5.96078900	1.45898500	3.27223200
C	-5.72341600	2.56272300	1.13531600
H	5.48894400	-3.48581600	1.70684200
H	5.32279900	0.72707100	2.46639300
H	9.12796400	-1.21233400	1.88122700
H	-5.86753900	-3.69407100	-1.37635100
C	-7.64083100	-3.42455400	-0.16628000
H	-5.31642400	-1.54765100	2.29356000
C	-7.33553400	-2.18199700	1.88793600
H	-5.30147900	3.24517600	-2.78652800
C	-7.15535400	2.27732900	-3.34367600
H	-5.31928800	-1.03503000	-2.55072300
C	-7.18193400	-0.13634200	-3.15360500
H	-5.54131500	0.86806200	4.07751300
C	-7.28954700	1.91221800	3.34846300
H	-5.10577700	2.80920700	0.28091500
C	-7.05467100	2.97808400	1.18770900
C	-8.17362500	-2.85703000	0.99898700
C	-8.50604000	-4.21309800	-1.08164600
C	-7.90055800	-1.55311200	3.11492300
C	-7.84183100	1.05987200	-3.44318800
C	-7.82344100	3.55768900	-3.72357700
C	-7.91122400	-1.43427300	-3.22721900
C	-7.84072200	2.65405900	2.29515900
C	-8.09095800	1.60785700	4.56202400
C	-7.64442100	3.74317300	0.05142100
H	-9.22977400	-2.94509600	1.22366500
O	-9.70334500	-4.50611600	-0.55948300
O	-8.18372100	-4.59164900	-2.20500700
O	-6.96977100	-1.38661600	4.06771000
O	-9.06593500	-1.21270100	3.23351900
H	-8.88968500	1.02046200	-3.72600200
O	-8.74253900	3.51356400	-4.69922100
O	-7.58331400	4.64214100	-3.20797300
O	-7.07160000	-2.46420800	-3.40986800
O	-9.12071000	-1.54586800	-3.11956800
H	-8.87170600	2.98510200	2.33185900
O	-9.22415000	2.31570900	4.64270400
O	-7.76794000	0.80404400	5.43293200
O	-6.69973800	4.31266000	-0.70850600
O	-8.84210700	3.82839600	-0.16486800
H	0.11603000	4.76274300	2.61069500
H	-0.42828500	-4.87990100	3.18887600
H	-0.46580600	-5.37133300	-2.07249500
H	-0.17455600	-0.84198200	-5.54308300
H	1.05368600	-2.15279700	-2.51166100
H	1.02372400	-1.65753300	2.99317600
H	1.29118000	2.90670900	-0.21041800
H	7.58073400	1.17556200	-2.58914500
H	9.28385300	-5.95959400	-1.47781900

H	8.16304900	-5.53611000	1.28295200
H	9.22810000	0.94382600	0.95761400
H	7.50108000	3.74787800	3.05980800
H	8.57640000	4.82891600	-4.06391800
H	-8.75694900	2.64526500	-5.13933300
H	-7.09574800	4.61049500	-1.57076900
H	-9.69473000	2.03208500	5.45077400
H	-7.32018600	-0.76287700	4.75253500
H	-10.20907900	-4.99907100	-1.23479100
H	-7.54505900	-3.31170400	-3.21248900
H	-0.22054300	0.41391700	5.62967500
O	-0.99170200	3.63829700	-1.48291600
H	-0.75716900	4.36341500	-0.87777900
H	-0.24316400	3.62103800	-2.20694900
C	1.45689000	2.48594800	-2.92435400
O	2.53523000	2.00501700	-3.42659100
O	0.93693400	3.61179500	-3.18380200

Sum of electronic and thermal Free Energies = -6879.689938 (Hartree/Particle).

Supplementary Table 16. DFT-optimized geometry of TS1 (singlet), computed at the B3LYP-D3/ LANL2DZ (Zr)/ 6-31G* (HCO) level in water solvent using PCM.

Atom	x	y	z
O	8.24303700	1.21470600	-1.84747200
O	7.20533100	3.07657000	-3.68378900
O	8.19703100	4.99990700	-3.04854900
O	9.34599300	-0.09997400	-0.46796400
C	8.43805200	0.02357900	-1.28544500
C	7.40685200	3.93644300	-2.83669700
C	7.52172600	-1.10633100	-1.60466700
C	6.82037900	3.89397100	-1.47185000
C	6.14483600	-0.90300800	-1.75586600
C	8.04698700	-2.40483600	-1.59355200
C	5.72059200	3.05822300	-1.26977000
C	7.35552900	4.60807300	-0.39784200
H	5.70785400	0.09095100	-1.73324000
C	5.28744400	-2.00758600	-1.82621400
H	9.11033600	-2.55046400	-1.44907800
C	7.19354500	-3.50093000	-1.74402900
H	5.27196200	2.54230500	-2.10974800
C	5.16850600	2.89964100	0.00251300
H	8.18722500	5.28724000	-0.54409900
C	6.83797100	4.42157600	0.88914200
C	5.81109500	-3.29931200	-1.81988300
C	3.80334600	-1.78637800	-1.79981500
C	7.69540200	-4.90381600	-1.77174400
C	5.74781200	3.56306300	1.09209600
C	3.94146700	2.03703200	0.12864600
C	7.44700200	5.22964200	2.00102600
H	5.14737600	-4.15496700	-1.86367900
O	3.12205000	-2.64459800	-1.15760400
O	3.36482000	-0.75595200	-2.38711000
O	9.03158800	-4.98416600	-1.60596700
O	6.98631400	-5.88101800	-1.92320100

H	5.32951800	3.42276800	2.08249000
O	3.53670400	1.51104400	-0.94218400
O	3.39609500	1.91776600	1.27243400
O	7.56317500	4.64772200	3.19869700
O	7.81308200	6.37863700	1.82612500
Zr	1.32003000	-2.38617300	0.21405900
Zr	1.62139200	0.59268100	-1.79896600
Zr	1.50145900	0.78942900	1.83061500
Zr	-1.51130600	-1.21868200	-1.58544800
Zr	-1.55329800	-1.05050500	1.81762200
O	3.15888400	-2.46914300	1.59658000
O	0.75891800	-1.55088000	-1.81110800
O	-0.68841100	-2.00675000	0.17990400
O	0.71208500	-1.33290000	2.14479800
O	1.83380900	-0.37485000	0.13566600
O	0.65185300	-3.98507900	1.70769700
O	0.68817700	-4.15518000	-1.06395200
Zr	-1.34003800	1.76165200	-0.06007200
O	-0.51780100	0.61357200	-1.55701300
O	0.97450200	1.97815700	-0.05729700
O	0.94105700	0.01790000	-3.95834500
O	7.53333900	-4.85244900	1.28327800
O	3.19877000	-0.45886000	2.62442700
O	-0.53702800	0.76223300	1.58287800
O	1.06257800	2.90822500	2.52055900
O	0.88459200	0.58388000	4.02254600
O	7.38175400	1.98667800	3.15563600
O	9.00600100	1.52981500	1.71688200
O	9.46580300	-3.69602300	1.40847500
O	-3.48504500	-2.35774400	-1.01437100
O	-3.12992400	0.00116300	-2.52342000
O	-2.31588200	-0.11779700	0.05343700
O	-1.03791300	-1.09557000	-3.80538400
O	-1.17986400	-3.44751600	-2.16008000
O	-3.34853100	0.22454300	2.56247400
O	-3.29242100	-2.33749600	1.23398100
O	-1.19073000	-3.14563200	2.75254100
O	-1.10542800	-0.52865100	4.02040700
C	3.75817700	-1.47612100	2.10705900
C	-0.33165300	-4.03669700	2.50639700
C	-0.32276600	-4.30602200	-1.81728500
O	-3.19997200	1.99458700	-1.46509100
O	-3.02443100	2.11811600	1.37790100
O	-0.74822900	3.59342000	1.32738400
C	-0.06982600	-0.56690700	-4.42950500
C	8.25034200	-3.72289000	1.46545700
C	0.14789000	3.72647100	2.20473100
C	-0.15270800	0.09741700	4.56452100
C	7.90537800	1.19079700	2.37880500
C	-3.95730000	-2.43218200	0.15511400
C	-3.71115700	1.06736000	-2.15360800
C	-3.73810400	1.31604000	2.05333200
C	5.25573600	-1.45860300	2.07420800
C	7.39819800	-2.53082600	1.72977900
C	7.29682000	-0.15293200	2.16657800
C	-5.43637700	-2.62500900	0.35197400
C	-5.14798400	1.16898200	-2.58817800
C	-5.17329300	1.72931200	2.21426400
C	6.00152500	-2.60609000	1.78720900
C	5.90548500	-0.24055400	2.27795700

C	8.04605200	-1.30164500	1.90560700
C	-6.26745000	-3.27044700	-0.56618100
C	-5.97602800	-2.12593300	1.54248700
C	-5.80582400	2.37653800	-2.82813400
C	-5.82697100	-0.03985000	-2.77158700
C	-5.96551600	1.36303300	3.30386400
C	-5.71310000	2.52783100	1.20100100
H	5.49106800	-3.54531600	1.61343900
H	5.31923000	0.64586200	2.48517500
H	9.12597500	-1.26963600	1.83582800
H	-5.85610000	-3.66745800	-1.48662200
C	-7.63554200	-3.42884500	-0.27910600
H	-5.32308300	-1.62132100	2.24304800
C	-7.34042000	-2.24222300	1.80945600
H	-5.28436200	3.31689600	-2.69303700
C	-7.13584200	2.36618200	-3.28634600
H	-5.30562100	-0.96836700	-2.57717400
C	-7.16441100	-0.05193100	-3.16423300
H	-5.55168700	0.74886600	4.09455500
C	-7.29449200	1.81468400	3.38422100
H	-5.08968600	2.79806200	0.35798200
C	-7.04477600	2.94166400	1.25632500
C	-8.17415300	-2.89248100	0.89818600
C	-8.49659400	-4.19091000	-1.22052000
C	-7.91140500	-1.64517900	3.04959900
C	-7.82232400	1.15235700	-3.42328700
C	-7.80055300	3.65726000	-3.63451900
C	-7.89318800	-1.34716800	-3.27965200
C	-7.83829400	2.58636300	2.34873000
C	-8.10411100	1.47693600	4.58343300
C	-7.62807200	3.73830700	0.13857000
H	-9.23162300	-2.98542900	1.11457300
O	-9.69464100	-4.50199200	-0.71088000
O	-8.17005400	-4.53474600	-2.35377700
O	-6.98534300	-1.50444400	4.01105500
O	-9.07728500	-1.30767700	3.17121900
H	-8.86867600	1.12141200	-3.71264800
O	-8.71728300	3.64047200	-4.61321600
O	-7.55935300	4.72736700	-3.09026400
O	-7.05228200	-2.37125300	-3.48739600
O	-9.10322900	-1.46177500	-3.18229500
H	-8.86948600	2.91648400	2.38775600
O	-9.23718800	2.18335000	4.67655400
O	-7.78773100	0.64811600	5.43307700
O	-6.67975900	4.33139900	-0.59839100
O	-8.82448900	3.82757300	-0.08304900
H	0.14728600	4.67751500	2.76301500
H	-0.44133300	-4.98189100	3.06393600
H	-0.45743700	-5.31952000	-2.23048400
H	-0.11984000	-0.63803000	-5.53001600
H	1.06312000	-2.09331400	-2.55339400
H	1.02083100	-1.75841600	2.95802800
H	1.29026300	2.88994000	-0.13843500
H	7.58329100	1.23517600	-2.56931500
H	9.27698100	-5.92835000	-1.64903300
H	8.16787800	-5.57613000	1.11890400
H	9.22392400	0.90921300	0.97481200
H	7.49239600	3.65487600	3.14693800
H	8.56707600	4.92213800	-3.94951100
H	-8.73219300	2.78411000	-5.07613000

H	-7.07203700	4.65297800	-1.45388200
H	-9.71352400	1.87748200	5.47306200
H	-7.33891400	-0.89936600	4.71086300
H	-10.19770300	-4.97514600	-1.40218500
H	-7.52658000	-3.22437400	-3.31813000
H	-0.22581600	0.23209200	5.65644900
O	-0.99725800	3.69270800	-1.33556000
H	-0.77784600	4.39061100	-0.69368800
H	-0.26003400	3.75069600	-2.07611000
C	1.41006700	3.08476300	-3.05788300
O	2.45798700	2.54861600	-3.55446200
O	0.78300400	4.18367000	-3.17654800

Sum of electronic and thermal Free Energies = -6879.643300 (Hartree/Particle).

Supplementary Table 17. DFT-optimized geometry of TS2 (doublet), computed at the UB3LYP-D3/ LANL2DZ (Zr)/ 6-31G* (HCO) level in water solvent using PCM.

Atom	x	y	z
O	8.27714100	1.24539200	-1.75529800
O	7.28531800	3.21487900	-3.49180300
O	8.28658100	5.09243100	-2.74280200
O	9.35219300	-0.18242800	-0.46955200
C	8.45195800	0.01429400	-1.28036000
C	7.48449700	4.02805800	-2.59946600
C	7.51560600	-1.07537000	-1.67802600
C	6.87675900	3.91990800	-1.24681400
C	6.14153300	-0.83848900	-1.79616300
C	8.01560900	-2.38127900	-1.76118200
C	5.75328900	3.10335100	-1.11004500
C	7.41171300	4.55709900	-0.12551300
H	5.72677000	0.15976800	-1.69959800
C	5.26248500	-1.92053400	-1.92956100
H	9.07725800	-2.55614300	-1.63748000
C	7.14044100	-3.44896000	-1.97783600
H	5.31163300	2.64839500	-1.98803700
C	5.17977400	2.88573400	0.14428800
H	8.25901100	5.22589600	-0.22159100
C	6.87418100	4.30567000	1.14221400
C	5.76146700	-3.21898700	-2.02323100
C	3.78752500	-1.67967900	-1.84705500
C	7.61512000	-4.85646600	-2.10740900
C	5.76404600	3.46166200	1.28108200
C	3.92949200	2.06262300	0.21398400
C	7.48266600	5.03714000	2.30741500
H	5.08203900	-4.05778100	-2.11875600
O	3.08710800	-2.59481900	-1.31599900
O	3.35506700	-0.56694300	-2.27045100
O	8.94750400	-4.97513700	-1.93755500
O	6.88713500	-5.80343500	-2.33726100
H	5.33131600	3.27018600	2.25653700
O	3.43636700	1.72670900	-0.91025000
O	3.43672700	1.77176100	1.34381200
O	7.58232000	4.38156500	3.46700700
O	7.85972700	6.19078600	2.20397400

Zr	1.29568600	-2.45744500	0.05861800
Zr	1.59629200	0.68820000	-1.60012200
Zr	1.50711200	0.65088600	1.94162300
Zr	-1.50192500	-1.01938600	-1.65956400
Zr	-1.56524800	-1.17145100	1.74630600
O	3.13143900	-2.58483000	1.40975300
O	0.77323700	-1.37395000	-1.90113100
O	-0.68723500	-1.96299300	0.02470100
O	0.70480600	-1.50115000	2.04710400
O	1.83287000	-0.39220300	0.13297300
O	0.56171600	-4.10957900	1.39099800
O	0.57234000	-4.02067700	-1.38308000
Zr	-1.36809500	1.82741300	0.17081700
O	-0.47035300	0.80379600	-1.42727300
O	0.98097800	2.04765800	0.11249200
O	1.04864600	0.58104500	-3.76154100
O	7.49901800	-4.98125100	0.94300100
O	3.19112300	-0.66325300	2.59213400
O	-0.51409000	0.65180200	1.65747500
O	1.11884400	2.69713400	2.76342600
O	0.87253200	0.28982100	4.06817600
O	7.37065300	1.73342300	3.24343700
O	9.00817600	1.34325600	1.79991100
O	9.43578800	-3.84585700	1.16100400
O	-3.45951800	-2.17649500	-1.22197800
O	-3.04096800	0.31157200	-2.51863500
O	-2.30418500	-0.06097600	0.06378700
O	-0.89793000	-0.59520500	-3.87147800
O	-1.20765200	-3.13433600	-2.49162500
O	-3.36136300	-0.01507600	2.60437100
O	-3.27568900	-2.37733800	1.01625900
O	-1.25280100	-3.31345400	2.50603600
O	-1.09467000	-0.85495100	3.97693000
C	3.74321700	-1.64270800	1.99717800
C	-0.42038000	-4.20526100	2.19096600
C	-0.40089100	-4.05883700	-2.19742900
O	-3.14568400	2.18296000	-1.26830100
O	-3.05108100	1.98998200	1.62754900
O	-0.74223400	3.46461700	1.70850200
C	0.04491400	0.07557000	-4.36458800
C	8.22019400	-3.87210600	1.21046400
C	0.18098800	3.52873600	2.56499600
C	-0.15742000	-0.25675300	4.57238300
C	7.89917300	0.97645400	2.43204000
C	-3.93571600	-2.38962400	-0.06950500
C	-3.64543200	1.32849000	-2.05458100
C	-3.76890200	1.11406700	2.20370000
C	5.23765200	-1.63601900	1.96665600
C	7.37257400	-2.69693600	1.55518900
C	7.28511100	-0.35062800	2.14308400
C	-5.40275400	-2.66137800	0.09064600
C	-5.07383600	1.45925900	-2.49535400
C	-5.21177700	1.48065100	2.37512700
C	5.97559500	-2.76780300	1.60475400
C	5.89370300	-0.43675200	2.24836800
C	8.02733300	-1.48592300	1.81296100
C	-6.20418500	-3.21517100	-0.91023500
C	-5.96547100	-2.33155700	1.32791000
C	-5.74310100	2.67889000	-2.61265200
C	-5.73574300	0.26763200	-2.81020400

C	-6.02121900	0.97339100	3.39419900
C	-5.74504600	2.37771600	1.44434700
H	5.46022500	-3.69082700	1.36933900
H	5.31399500	0.43899100	2.51092600
H	9.10763700	-1.45748100	1.74851300
H	-5.77294400	-3.47991100	-1.86817900
C	-7.56713400	-3.45335100	-0.65891600
H	-5.33524700	-1.89641500	2.09260500
C	-7.32664900	-2.52384500	1.56334500
H	-5.23542800	3.60560900	-2.37161600
C	-7.06687500	2.70121500	-3.08689800
H	-5.20687300	-0.67102300	-2.70596000
C	-7.06902700	0.28246300	-3.21587600
H	-5.61310900	0.28151800	4.12127700
C	-7.36207700	1.38439500	3.48799400
H	-5.10917000	2.75878500	0.65558300
C	-7.08816800	2.75008300	1.50684900
C	-8.13105100	-3.08549900	0.57027600
C	-8.39662400	-4.11776000	-1.69835700
C	-7.92611200	-2.08997300	2.85679800
C	-7.73651100	1.50087900	-3.35880400
C	-7.74546700	4.01473200	-3.30044200
C	-7.78739100	-1.00011100	-3.46723200
C	-7.89912300	2.25465500	2.52950700
C	-8.19156500	0.90181100	4.62353100
C	-7.66300600	3.65248300	0.46709500
H	-9.18648000	-3.23795300	0.76161600
O	-9.57494900	-4.55260400	-1.23690300
O	-8.06106600	-4.28441300	-2.86809800
O	-7.00591200	-2.00127900	3.83054200
O	-9.10668400	-1.82439100	3.00739600
H	-8.77913800	1.48908400	-3.66273000
O	-8.63904300	4.09925700	-4.29606000
O	-7.53471600	5.01615100	-2.62822700
O	-6.93577900	-2.00290600	-3.72545900
O	-8.99977100	-1.12146700	-3.42710900
H	-8.93916900	2.55446000	2.57622000
O	-9.35930600	1.54546700	4.73906100
O	-7.86104600	0.01557200	5.40653400
O	-6.70990800	4.33573400	-0.17968500
O	-8.85572300	3.74436800	0.22885200
H	0.18830900	4.41833400	3.21311700
H	-0.54804800	-5.18738800	2.67115200
H	-0.54818500	-5.01814700	-2.71678000
H	0.01708900	0.24387300	-5.45197200
H	1.07190000	-1.82113400	-2.70803300
H	1.00153700	-1.98821700	2.83074100
H	1.37776100	2.93138700	0.15723300
H	7.62779700	1.32762900	-2.48224300
H	9.17746000	-5.91692800	-2.05544500
H	8.13161500	-5.69320100	0.72745700
H	9.23268900	0.76038500	1.03024300
H	7.50195500	3.39460800	3.35248000
H	8.67002700	5.06044400	-3.64107400
H	-8.62695400	3.30553500	-4.85977900
H	-7.08903700	4.74853800	-1.00135200
H	-9.84411900	1.14910900	5.48923500
H	-7.38374100	-1.48063700	4.58228300
H	-10.06095900	-4.94949400	-1.98612400
H	-7.41237300	-2.86882200	-3.65088800

H	-0.22990700	-0.20702500	5.66979100
O	-1.01891100	3.85161000	-0.91157000
H	-0.60965400	4.49111600	-0.30431600
H	-0.52651700	3.93369300	-1.79489800
C	1.48114800	3.84267400	-3.09254100
O	0.30020100	4.24839100	-3.12341200
O	2.51139900	4.03480100	-3.71903300

Sum of electronic and thermal Free Energies = -6879.539603 (Hartree/Particle).

Supplementary Table 18. DFT-optimized geometry of f-[Zr^{IV}(BTC)(BTC)(H_2O)($COOH^-$)]$^-$ (singlet), computed at the B3LYP-D3/ LANL2DZ (Zr)/ 6-31G* (HCO) level in water solvent using PCM.

Atom	x	y	z
O	8.17749500	1.22005800	-1.89237600
O	7.11280800	3.10526400	-3.71084400
O	8.10743700	5.02735600	-3.07517100
O	9.29975300	-0.08797500	-0.52077300
C	8.38615800	0.03039600	-1.33207300
C	7.32416900	3.95946100	-2.86087200
C	7.47536600	-1.10677100	-1.64398300
C	6.75899300	3.90330600	-1.48715200
C	6.09399300	-0.91475700	-1.75863400
C	8.01189300	-2.40068000	-1.65524600
C	5.66481500	3.06249800	-1.27608400
C	7.31236400	4.60561600	-0.41463000
H	5.64969800	0.07522500	-1.71605400
C	5.24597300	-2.02797000	-1.81311100
H	9.07959400	-2.53822300	-1.53739800
C	7.16515400	-3.50377500	-1.79317900
H	5.19768700	2.56158100	-2.11548900
C	5.14154300	2.88609500	0.00644500
H	8.14038500	5.28763700	-0.56832600
C	6.81830700	4.40572600	0.87987000
C	5.77943500	-3.31553100	-1.83072200
C	3.76636900	-1.81653400	-1.73340100
C	7.67947200	-4.90219100	-1.84217200
C	5.73546700	3.54157400	1.09318400
C	3.93148600	2.00977900	0.14919100
C	7.44410500	5.20620300	1.98836000
H	5.12307300	-4.17736700	-1.86337800
O	3.09985500	-2.67527300	-1.08084000
O	3.30167400	-0.77494500	-2.28783400
O	9.01897500	-4.97006400	-1.70624800
O	6.97512400	-5.88406400	-1.98408500
H	5.33504500	3.38891300	2.08898900
O	3.52622100	1.46532200	-0.91638700
O	3.38906000	1.88966800	1.29398900
O	7.57073500	4.62060400	3.18284600
O	7.81286700	6.35411100	1.81260000
Zr	1.28804000	-2.43206000	0.27529800
Zr	1.59184500	0.53789900	-1.67064100
Zr	1.49733900	0.78172300	1.90032400

Zr	-1.50979300	-1.19369700	-1.57067500
Zr	-1.57731000	-1.05069600	1.84471000
O	3.11508200	-2.49746100	1.65970200
O	0.75130100	-1.56404400	-1.76651100
O	-0.70359500	-1.98972800	0.20146400
O	0.68034100	-1.35006000	2.19170000
O	1.82362800	-0.40002000	0.20579200
O	0.58546300	-4.00163700	1.74122300
O	0.62015600	-4.15997300	-1.02419100
Zr	-1.35799700	1.79416900	0.01113000
O	-0.47588300	0.66656000	-1.52048300
O	0.98633100	2.01316500	-0.04045900
O	1.03497200	0.15242400	-3.84523700
O	7.48339500	-4.86323800	1.20916700
O	3.17159800	-0.48377000	2.67814700
O	-0.53081300	0.75958800	1.60745300
O	1.06246800	2.90117200	2.55095600
O	0.84339900	0.58767100	4.05753900
O	7.35661500	1.96097500	3.14631200
O	8.96339600	1.51457300	1.68640100
O	9.41723100	-3.70647300	1.31237800
O	-3.46407600	-2.30426200	-1.03283700
O	-3.06136500	0.07755500	-2.51610700
O	-2.30137500	-0.09133400	0.06553200
O	-0.93442000	-0.99141400	-3.79390400
O	-1.19400200	-3.39549000	-2.17196800
O	-3.37741600	0.21745100	2.56886500
O	-3.30997500	-2.30565800	1.21811500
O	-1.25385100	-3.13931700	2.76794900
O	-1.14274100	-0.52854700	4.03938900
C	3.72525200	-1.50270800	2.15477000
C	-0.40639500	-4.04319600	2.53243000
C	-0.36743400	-4.27912000	-1.81316800
O	-3.18669700	2.03446900	-1.40319600
O	-3.03333800	2.12445600	1.42206800
O	-0.76215700	3.58421600	1.38069300
C	0.03142200	-0.41817600	-4.37032700
C	8.20327500	-3.73585000	1.39162200
C	0.14378600	3.71976400	2.24912500
C	-0.19889200	0.10112000	4.59283900
C	7.87154100	1.16967300	2.35887200
C	-3.95695800	-2.39892100	0.12899000
C	-3.67014100	1.12524900	-2.13398700
C	-3.76444700	1.31374700	2.07264100
C	5.22026400	-1.48259800	2.09217400
C	7.35513200	-2.54746500	1.68568100
C	7.26074500	-0.17348100	2.14719800
C	-5.43387100	-2.61411200	0.29462300
C	-5.09253900	1.22516200	-2.60519400
C	-5.20065600	1.72170000	2.21189400
C	5.96070600	-2.62650600	1.77643300
C	5.87274600	-0.26520700	2.29214700
C	8.00491100	-1.31842900	1.85673100
C	-6.24071700	-3.24727700	-0.65347100
C	-5.99956900	-2.14475300	1.48481100
C	-5.75085300	2.43418600	-2.83637000
C	-5.75829600	0.01583400	-2.82895900
C	-6.01427300	1.32875300	3.27682700
C	-5.72182000	2.53627600	1.20152900
H	5.44825400	-3.56546800	1.60725000

H	5.29144400	0.61822300	2.52377100
H	9.08277700	-1.28427200	1.76068300
H	-5.80844800	-3.62123900	-1.57392800
C	-7.61220700	-3.42285500	-0.39572700
H	-5.36486400	-1.65006400	2.20860600
C	-7.36751100	-2.27690700	1.72249000
H	-5.23827200	3.37435100	-2.67014300
C	-7.06915900	2.42550900	-3.32624900
H	-5.23648700	-0.91369500	-2.64087900
C	-7.08659100	0.00470600	-3.25131000
H	-5.61507400	0.70106500	4.06457100
C	-7.34669300	1.77254100	3.33617200
H	-5.08236000	2.82577000	0.37718800
C	-7.05747000	2.93906500	1.23404900
C	-8.17776400	-2.91513600	0.78191300
C	-8.44854400	-4.17190700	-1.37010500
C	-7.96531500	-1.70417800	2.96168400
C	-7.74480400	1.21064700	-3.50221100
C	-7.73252600	3.72045200	-3.66361600
C	-7.80827000	-1.29092100	-3.40438900
C	-7.87203900	2.55907200	2.30225000
C	-8.18070200	1.41292800	4.51281200
C	-7.62366500	3.74816900	0.11615000
H	-9.23855900	-3.02032300	0.97530200
O	-9.65296700	-4.50351300	-0.89012200
O	-8.09695700	-4.48835200	-2.50364100
O	-7.05471100	-1.56554200	3.93814800
O	-9.13616400	-1.38027000	3.06891000
H	-8.78419400	1.17911800	-3.81570100
O	-8.63162500	3.71845300	-4.65813600
O	-7.50393400	4.78115800	-3.09610400
O	-6.95886000	-2.31211000	-3.58976300
O	-9.02062500	-1.40739200	-3.35096000
H	-8.90598800	2.88197100	2.32414000
O	-9.32155000	2.10848300	4.58711900
O	-7.87573000	0.57792500	5.36019700
O	-6.66486500	4.35498400	-0.59538000
O	-8.81608300	3.83383100	-0.12639800
H	0.14736300	4.66967700	2.80646100
H	-0.53187400	-4.98601800	3.08777600
H	-0.51063400	-5.28208200	-2.24552400
H	0.00804100	-0.41986000	-5.47197100
H	1.05184100	-2.09056200	-2.52214500
H	0.97488200	-1.77921100	3.00897200
H	1.35671700	2.90853400	-0.06504800
H	7.51220700	1.23436900	-2.60900000
H	9.27369000	-5.91136600	-1.75795100
H	8.11425600	-5.58587800	1.02695900
H	9.17570500	0.90110200	0.93717900
H	7.49123600	3.62823400	3.13255000
H	8.46576000	4.95827600	-3.98158100
H	-8.63610600	2.87095200	-5.13727200
H	-7.04372300	4.68634800	-1.45336800
H	-9.81379900	1.79040200	5.36907900
H	-7.42214800	-0.97044600	4.63893200
H	-10.13908600	-4.96681900	-1.60003200
H	-7.43671800	-3.16739900	-3.44314300
H	-0.28222000	0.24082100	5.68219000
O	-0.96973800	3.67363800	-1.31351800
H	-0.81627800	4.42344100	-0.71109700

H	-0.14095900	3.62511000	-1.90002100
C	1.88769900	2.63767100	-2.81250400
O	1.19791900	3.67183700	-2.75044800
O	2.97832300	2.70178300	-3.62405400
H	3.00385100	3.60122400	-4.02271700

Sum of electronic and thermal Free Energies = -6880.149762 (Hartree/Particle).

Supplementary Table 19. DFT-optimized geometry of f-[Zr^{IV}(BTC)(BTC)(H_2O)(CO)] (singlet), computed at the B3LYP-D3/ LANL2DZ (Zr)/ 6-31G* (HCO) level in water solvent using PCM.

Atom	x	y	z
O	8.40125900	0.90548600	-1.81338700
O	7.43015400	2.29879200	-4.01209400
O	8.38360400	4.32620000	-3.75095600
O	9.40866400	-0.18504500	-0.18695600
C	8.54749100	-0.18245100	-1.06160100
C	7.58407100	3.32496300	-3.36204800
C	7.61960100	-1.33485900	-1.23996500
C	6.91693600	3.56857600	-2.05582300
C	6.25699200	-1.12851500	-1.48264200
C	8.10383900	-2.62408500	-0.98468800
C	5.77438600	2.82132100	-1.76683400
C	7.41507000	4.46790700	-1.11095600
H	5.85588300	-0.13328100	-1.64211800
C	5.36960000	-2.20819400	-1.40421700
H	9.15453800	-2.76640300	-0.76384700
C	7.22486200	-3.71055200	-0.98993900
H	5.36534700	2.15744400	-2.51849100
C	5.14317100	2.93724100	-0.52590600
H	8.27620800	5.08582000	-1.33716500
C	6.82336100	4.54788600	0.15477800
C	5.85441300	-3.49398800	-1.16427700
C	3.89531000	-1.95656500	-1.43856500
C	7.68222100	-5.11025700	-0.75371000
C	5.69388200	3.77405600	0.45457000
C	3.87238100	2.17344200	-0.30925000
C	7.39679800	5.54658900	1.12306400
H	5.16827100	-4.32966400	-1.09428900
O	3.17306800	-2.75243700	-0.76035800
O	3.47906200	-0.95296200	-2.09121500
O	9.00042300	-5.18901900	-0.48065800
O	6.95230600	-6.08218000	-0.79594800
H	5.22037600	3.83772300	1.42787700
O	3.39811000	1.61417800	-1.35427200
O	3.34643400	2.13447300	0.84016600
O	7.45724000	5.19589800	2.41018000
O	7.78120800	6.64041200	0.75006000
Zr	1.33699900	-2.34589400	0.46161900
Zr	1.65548100	0.35285500	-1.81422800
Zr	1.41388300	1.08740800	1.63652800
Zr	-1.40747600	-1.41177600	-1.65209800
Zr	-1.61369000	-0.83291500	1.73401700

O	3.10817900	-2.11862800	1.88673100
O	0.88863100	-1.73942000	-1.70880400
O	-0.64711700	-1.93272400	0.23520700
O	0.64719200	-1.01066900	2.17341100
O	1.84130700	-0.30374100	0.11565900
O	0.65427300	-3.71074900	2.08836800
O	0.71376500	-4.21501500	-0.63474800
Zr	-1.39518400	1.74390600	-0.44176200
O	-0.40505400	0.44976100	-1.78189000
O	0.92139700	2.03731600	-0.46930500
O	1.31138700	-0.21417000	-3.93765500
O	7.48113100	-4.50341700	2.23643200
O	3.09675400	0.02432200	2.60797900
O	-0.60134500	0.96038100	1.29510800
O	0.90244500	3.25497200	1.97243700
O	0.71992000	1.15326300	3.76570100
O	7.24013700	2.57185000	2.82521100
O	8.95698100	1.85142300	1.62066300
O	9.40662900	-3.32824800	2.26124500
O	-3.36331100	-2.49817500	-1.02461600
O	-2.95559500	-0.31461100	-2.81805500
O	-2.28999400	-0.13037800	-0.20093300
O	-0.63946300	-1.38714800	-3.89294300
O	-1.02134600	-3.63517600	-1.99122900
O	-3.43758700	0.46017000	2.24294500
O	-3.25981400	-2.20264200	1.20563200
O	-1.24420900	-2.76881800	2.91090400
O	-1.24517900	0.00281000	3.84131600
C	3.68317400	-1.05920600	2.27729300
C	-0.36779000	-3.67109000	2.84396300
C	-0.21515500	-4.45393600	-1.46415800
O	-3.03774000	1.78978000	-2.02355800
O	-3.15910200	2.19334000	0.82642900
O	-0.90178100	3.71415100	0.67091800
C	0.33248200	-0.84445600	-4.47012800
C	8.19012500	-3.35534100	2.26369800
C	-0.03397800	3.99473600	1.54383900
C	-0.33375400	0.72368500	4.33201800
C	7.81240800	1.64177900	2.26125500
C	-3.88395200	-2.45347800	0.12643900
C	-3.57267500	0.75806900	-2.53954600
C	-3.83661200	1.51043100	1.65681100
C	5.17606900	-1.03696200	2.32250800
C	7.32785000	-2.14090100	2.28099500
C	7.21202100	0.27727300	2.28030600
C	-5.35536500	-2.68436800	0.30567200
C	-5.04295400	0.77167700	-2.79918100
C	-5.21434900	2.03406700	1.94358900
C	5.93055800	-2.21396700	2.27828000
C	5.81686400	0.20288900	2.33724700
C	7.96907000	-0.89580500	2.27205600
C	-6.13553200	-3.46776800	-0.54696700
C	-5.94237000	-2.07795800	1.42146000
C	-5.78711600	1.95273200	-2.81254000
C	-5.68445500	-0.46076500	-2.95901400
C	-5.93353000	1.66901900	3.08526600
C	-5.76993000	2.94057000	1.03300900
H	5.42706300	-3.17226400	2.24466900
H	5.22534100	1.10967100	2.35217300
H	9.05115400	-0.87203200	2.25553300

H	-5.68875800	-3.94802100	-1.40935500
C	-7.50029900	-3.65534800	-0.26450900
H	-5.32903700	-1.46816100	2.07154600
C	-7.30313800	-2.23241300	1.68406300
H	-5.29662200	2.91213100	-2.69435400
C	-7.18058500	1.89764500	-2.96015600
H	-5.09721100	-1.36899300	-2.93115900
C	-7.07112600	-0.51946100	-3.09412100
H	-5.50827400	0.96338100	3.78839600
C	-7.19231700	2.24280500	3.32982000
H	-5.20759000	3.20831000	0.14841000
C	-7.03598000	3.48534100	1.25686900
C	-8.08626200	-3.02174600	0.83995500
C	-8.30534300	-4.54782500	-1.13945100
C	-7.91685600	-1.54594900	2.85838000
C	-7.82259100	0.65956100	-3.07993300
C	-7.93221300	3.18873100	-3.00908000
C	-7.76341700	-1.83671200	-3.17445100
C	-7.74165000	3.14722400	2.41373600
C	-7.93303900	1.89393600	4.57114000
C	-7.66554400	4.42121800	0.28248900
H	-9.14157800	-3.14499700	1.05213500
O	-9.49661300	-4.86593800	-0.61972900
O	-7.93658500	-4.98141200	-2.22754500
O	-6.99080600	-1.19716400	3.76306700
O	-9.10981100	-1.32470000	2.97699900
H	-8.89972400	0.58453300	-3.19469500
O	-9.20011700	3.19339000	-2.54620400
O	-7.45125600	4.22329100	-3.43109000
O	-6.92643100	-2.82881200	-3.51279700
O	-8.94748900	-1.99646600	-2.92809700
H	-8.71626500	3.58708900	2.58718500
O	-8.88734900	2.77825200	4.88086800
O	-7.71323700	0.90836700	5.26956900
O	-6.82092900	4.76075200	-0.71175400
O	-8.80943000	4.82796200	0.36143600
H	-0.08347900	5.00469200	1.97768400
H	-0.48853700	-4.52635200	3.52527500
H	-0.32345200	-5.50552000	-1.76999100
H	0.36966800	-0.92001800	-5.56728800
H	1.23984900	-2.34488300	-2.38000700
H	0.92578300	-1.31095600	3.05203900
H	1.28825900	2.92986900	-0.56582400
H	7.79211600	0.81501400	-2.57511300
H	9.22173400	-6.13275200	-0.36098000
H	8.12220700	-5.23976000	2.21780300
H	9.22363400	1.09849900	1.03388900
H	7.37693600	4.21035300	2.53764400
H	8.81070300	4.06277400	-4.58967700
H	-9.42342700	2.36123700	-2.09421600
H	-7.31366400	5.26713600	-1.38741100
H	-9.34527200	2.45796900	5.68282700
H	-7.37595600	-0.57096100	4.42849300
H	-9.96384300	-5.42803500	-1.26822700
H	-7.35802100	-3.69459400	-3.30171700
H	-0.45471900	1.01276300	5.38696700
O	-0.99497700	3.48147600	-2.03709900
H	-1.73193500	3.50933100	-2.67492600
H	-0.95879100	4.35716600	-1.61001400
C	1.34443800	2.52093000	-3.44162000

O	1.20865300	3.31403700	-4.23806200

Sum of electronic and thermal Free Energies = -6804.177291 (Hartree/Particle).

Supplementary Table 20. DFT-optimized geometry of f-$[Zr^{IV}(BTC^{\cdot -})(BTC)(H_2O)(CO)]^-$ (doublet), computed at the UB3LYP-D3/ LANL2DZ (Zr)/ 6-31G* (HCO) level in water solvent using PCM. The spin density plot is also shown below. (Isovalue = 0.0004)

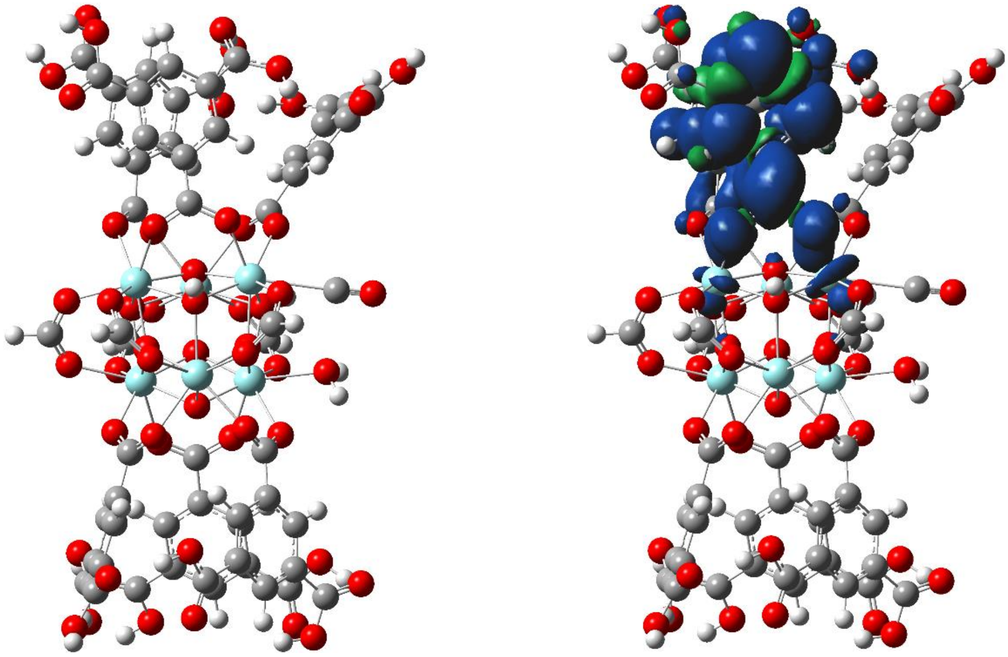

Spin Density Distribution

Atom	x	y	z
O	8.26135800	0.96938200	-1.89251400
O	7.32300500	2.61535600	-4.05241700
O	8.44546300	4.51032700	-3.56624000
O	9.37232700	-0.10566800	-0.32142800
C	8.45648800	-0.13192600	-1.14910000
C	7.55660500	3.54190200	-3.29050900
C	7.55595100	-1.29049700	-1.29723900
C	6.90186200	3.70217100	-1.96489100
C	6.20705100	-1.09361000	-1.57548000
C	8.05302600	-2.57649900	-0.95254600
C	5.76961300	2.92862000	-1.70686400
C	7.39851100	4.55308400	-0.97519300
H	5.81603500	-0.10406500	-1.79297600
C	5.27441700	-2.15386200	-1.41560000
H	9.10437300	-2.71334800	-0.74265200
C	7.14069100	-3.64978300	-0.88056600
H	5.36467500	2.29656900	-2.48723300
C	5.14753600	2.96740700	-0.45692300
H	8.25362100	5.18912400	-1.17134700
C	6.81225800	4.56241900	0.29516800
C	5.77975100	-3.43515100	-1.05188200
C	3.86036100	-1.93623500	-1.52513600

C	7.57825200	-5.04205300	-0.60822000
C	5.69297100	3.76139900	0.56051000
C	3.89358300	2.16622200	-0.27002500
C	7.38669900	5.50746300	1.31433200
H	5.09133800	-4.26344000	-0.93976300
O	3.05015900	-2.80011500	-0.97274600
O	3.40568000	-0.88863500	-2.16129500
O	8.91831400	-5.15236100	-0.43105500
O	6.84374300	-6.01553400	-0.55533100
H	5.22682400	3.76640800	1.53917500
O	3.41783800	1.65873700	-1.33507000
O	3.38473100	2.05359400	0.88439600
O	7.45905000	5.08064700	2.57718400
O	7.76444000	6.62373400	1.00387700
Zr	1.34712900	-2.41379900	0.29983400
Zr	1.68792000	0.36326700	-1.87339200
Zr	1.45120100	0.96727100	1.62133600
Zr	-1.42878600	-1.35551500	-1.71742800
Zr	-1.58817300	-0.92066500	1.67243600
O	3.14316600	-2.25918200	1.71986800
O	0.83329100	-1.70635500	-1.83129700
O	-0.66402800	-1.96954400	0.13015600
O	0.66746300	-1.13919200	2.08557300
O	1.83303600	-0.35245000	0.04990900
O	0.66262500	-3.83088800	1.90192300
O	0.63221900	-4.24881700	-0.83771100
Zr	-1.35807000	1.73190200	-0.39679800
O	-0.41076300	0.48870400	-1.78615100
O	0.95029700	2.01026100	-0.42772300
O	1.19418600	-0.08663200	-4.01125500
O	7.58915200	-4.58194500	2.33261700
O	3.11452900	-0.14339900	2.52179600
O	-0.57682600	0.88865300	1.31420400
O	0.96315500	3.14899100	2.06222900
O	0.76967900	0.96317100	3.77437800
O	7.22008100	2.45303300	2.82036100
O	8.92122100	1.80478600	1.54981600
O	9.48904700	-3.36855100	2.32616100
O	-3.41073000	-2.43901000	-1.11547400
O	-3.00299900	-0.20824000	-2.82973000
O	-2.29560500	-0.12568900	-0.20905000
O	-0.75241600	-1.26865000	-3.96649200
O	-1.10160500	-3.57255000	-2.14966700
O	-3.39333200	0.39404400	2.26483600
O	-3.28075800	-2.24220600	1.12783200
O	-1.23395300	-2.92114400	2.76838800
O	-1.19850800	-0.18561100	3.81925600
C	3.71537400	-1.21149700	2.14654900
C	-0.35598200	-3.81844400	2.66064800
C	-0.30779100	-4.42972300	-1.66557100
O	-3.04807700	1.86483200	-1.94892500
O	-3.11277100	2.17936400	0.91147300
O	-0.83514900	3.67643400	0.77467800
C	0.20748700	-0.69828000	-4.54161400
C	8.27229200	-3.41722300	2.29447200
C	0.03505300	3.91185200	1.65987900
C	-0.27876100	0.51107300	4.33009200
C	7.79064900	1.55430400	2.19987600
C	-3.91500100	-2.43873900	0.04258000
C	-3.59986700	0.85900700	-2.49657300

C	-3.78529200	1.46904400	1.72178700
C	5.20271100	-1.17207900	2.20475000
C	7.38263100	-2.22775300	2.22703900
C	7.21351400	0.18515100	2.17842800
C	-5.38871800	-2.66235200	0.22674200
C	-5.07637300	0.89773500	-2.73290700
C	-5.15826100	1.99318700	2.04297000
C	5.98650600	-2.33628700	2.19747800
C	5.81946500	0.07955500	2.22190300
C	7.99792800	-0.97198500	2.18965900
C	-6.18365200	-3.40921500	-0.64456000
C	-5.96073500	-2.09087300	1.36796000
C	-5.81077300	2.08411300	-2.69208900
C	-5.73135500	-0.32158700	-2.93345900
C	-5.87289100	1.58950000	3.17446700
C	-5.71350700	2.93987200	1.17437000
H	5.50806400	-3.30692000	2.18011000
H	5.20944700	0.97406100	2.22645400
H	9.07925000	-0.92106000	2.18596000
H	-5.74903500	-3.86186600	-1.52786900
C	-7.54734100	-3.59651500	-0.35563100
H	-5.33586800	-1.50822400	2.03182800
C	-7.31988100	-2.24533000	1.63966500
H	-5.31075400	3.03404300	-2.54158800
C	-7.20665000	2.04648500	-2.82639800
H	-5.15117300	-1.23461700	-2.94666300
C	-7.11976800	-0.36365900	-3.05746900
H	-5.44960900	0.85148900	3.84496200
C	-7.12472000	2.16454500	3.45212900
H	-5.15489000	3.23727700	0.29699500
C	-6.97310300	3.48647800	1.42906500
C	-8.11777100	-2.99861100	0.77659900
C	-8.36738500	-4.45001500	-1.25468800
C	-7.91363600	-1.59785400	2.84563500
C	-7.86087100	0.81985900	-2.98880700
C	-7.94987300	3.34285300	-2.81650200
C	-7.82408900	-1.67036600	-3.18592300
C	-7.67321300	3.10923400	2.57706300
C	-7.85931700	1.77441900	4.68447600
C	-7.60195300	4.46305700	0.49543500
H	-9.17172700	-3.12238500	0.99519600
O	-9.55776600	-4.77630800	-0.73719500
O	-8.01192800	-4.84756900	-2.36094700
O	-6.97351100	-1.29506000	3.75257400
O	-9.10261400	-1.36779000	2.98668800
H	-8.93956600	0.75864900	-3.09637700
O	-9.21252300	3.33630000	-2.33887600
O	-7.46811000	4.39097200	-3.20335900
O	-6.99961100	-2.65447600	-3.57520600
O	-9.00724500	-1.83097000	-2.93484000
H	-8.64271900	3.54987200	2.77525100
O	-8.80615700	2.65256600	5.03384700
O	-7.64159900	0.76270600	5.34536100
O	-6.76196100	4.83313600	-0.49199400
O	-8.74246100	4.87477900	0.59695000
H	-0.00642300	4.90590100	2.13222500
H	-0.47517500	-4.70023300	3.30945500
H	-0.44442300	-5.46513700	-2.01616300
H	0.21568800	-0.73165400	-5.64252300
H	1.20755500	-2.29918300	-2.50108500

H	0.95278300	-1.47370500	2.94895700
H	1.31622800	2.90684400	-0.46856600
H	7.61207000	0.84296300	-2.60947900
H	9.10642400	-6.09909400	-0.28640000
H	8.24894300	-5.30081900	2.35099700
H	9.17016500	1.08163900	0.91057100
H	7.37810700	4.08625500	2.64228900
H	8.84682800	4.30733500	-4.43344800
H	-9.43439500	2.48844600	-1.91619200
H	-7.25796800	5.36104100	-1.14858100
H	-9.26032100	2.30462300	5.82633200
H	-7.34207200	-0.68920600	4.44533200
H	-10.03458000	-5.31004000	-1.40244600
H	-7.43577400	-3.52456300	-3.39371200
H	-0.38978400	0.75398800	5.39866600
O	-0.95177000	3.51510400	-1.94007600
H	-1.70306900	3.57139400	-2.55910900
H	-0.90995800	4.36918600	-1.47171600
C	1.50823400	2.67223500	-3.28059200
O	1.48156500	3.61700600	-3.90439000

Sum of electronic and thermal Free Energies = -6804.280591 (Hartree/Particle).

Supplementary Table 21. DFT-optimized geometry of f-$[Zr^{IV}(BTC^{\cdot -})(BTC)(H_2O)(CHO)]^-$ (singlet), computed at the B3LYP-D3/ LANL2DZ (Zr)/ 6-31G* (HCO) level in water solvent using PCM.

Atom	x	y	z
O	8.24483900	1.16096400	-1.96421100
O	7.18599300	2.94513000	-3.85416700
O	8.19026000	4.88713200	-3.29991100
O	9.34495900	-0.10021100	-0.53283900
C	8.43796800	-0.00670000	-1.35494000
C	7.39684500	3.83688900	-3.04311500
C	7.51654400	-1.14579400	-1.62484100
C	6.81666100	3.85155100	-1.67447800
C	6.13917500	-0.94255300	-1.76705400
C	8.03613600	-2.44515100	-1.56734900
C	5.71194800	3.03289800	-1.43530500
C	7.35955500	4.60536100	-0.63188500
H	5.70885600	0.05413900	-1.77927200
C	5.27647400	-2.04526200	-1.77983100
H	9.10036900	-2.59002000	-1.42868300
C	7.17651500	-3.54267500	-1.66348500
H	5.25979100	2.48601600	-2.25337100
C	5.16253700	2.93179500	-0.15566600
H	8.19420500	5.27352500	-0.80873400
C	6.84600600	4.47443500	0.66355300
C	5.79411500	-3.33865700	-1.72877900
C	3.79704100	-1.81501200	-1.72751200
C	7.67332400	-4.94788800	-1.64037900
C	5.75131800	3.63254400	0.90535900
C	3.92999600	2.09003200	0.01151000
C	7.46368500	5.32397300	1.73976500

H	5.12652700	-4.19238400	-1.72806800
O	3.11824700	-2.65370700	-1.05860200
O	3.34842700	-0.78788700	-2.31547300
O	9.01097100	-5.02532200	-1.48928800
O	6.95845200	-5.92704100	-1.74244100
H	5.33628100	3.53485900	1.90216400
O	3.49070700	1.55983500	-1.05051800
O	3.40826400	1.98024400	1.16500800
O	7.58540700	4.78866900	2.95798500
O	7.83059100	6.46415500	1.51693600
Zr	1.32282900	-2.35674900	0.28668500
Zr	1.61056600	0.57011100	-1.76659100
Zr	1.51665600	0.87651800	1.82924800
Zr	-1.48808900	-1.21845900	-1.57010100
Zr	-1.55025300	-0.98492700	1.85700300
O	3.15729600	-2.38591500	1.66677600
O	0.76668800	-1.56836200	-1.77501000
O	-0.67616400	-1.94784900	0.22498900
O	0.71681600	-1.25009700	2.18673200
O	1.82953600	-0.32303500	0.16394400
O	0.65064900	-3.90850000	1.80522900
O	0.67114300	-4.14172800	-0.95231200
Zr	-1.34379600	1.77501300	-0.05221800
O	-0.48573400	0.64159800	-1.56701800
O	0.94973500	2.06644800	-0.12712500
O	1.00368600	0.11082800	-3.91060200
O	7.53167400	-4.78088800	1.41682900
O	3.20531700	-0.34149600	2.62293300
O	-0.52392400	0.82808200	1.58097000
O	1.09218900	3.00017600	2.45450700
O	0.88315300	0.71450900	4.00794500
O	7.38575200	2.12892300	3.01582400
O	9.00309500	1.61466500	1.58917200
O	9.46364800	-3.61747500	1.47202500
O	-3.44679900	-2.32189900	-0.98027600
O	-3.07359900	-0.01077500	-2.54757100
O	-2.29776100	-0.07494900	0.05021800
O	-0.93738400	-1.07526500	-3.79720000
O	-1.16761200	-3.43525400	-2.09715100
O	-3.34655700	0.31257500	2.54629400
O	-3.27382300	-2.26798300	1.26837800
O	-1.19608900	-3.04639500	2.81821100
O	-1.10840100	-0.39490000	4.02819400
C	3.76297300	-1.37809200	2.13849800
C	-0.33557800	-3.94395500	2.60120700
C	-0.32596600	-4.29810300	-1.72120500
O	-3.15897200	2.01446100	-1.56130700
O	-3.04285000	2.17627400	1.31345500
O	-0.75747300	3.64380500	1.30455000
C	0.01341600	-0.50590900	-4.40401800
C	8.24917500	-3.64438500	1.54396200
C	0.15623700	3.80314700	2.15744100
C	-0.15788600	0.24620300	4.55943100
C	7.90664600	1.30198800	2.27028000
C	-3.92986900	-2.38705600	0.18638600
C	-3.66517700	1.06415300	-2.22808800
C	-3.75312800	1.38294000	2.00865400
C	5.25836500	-1.36062500	2.09520400
C	7.39784500	-2.44331100	1.76946500
C	7.29813700	-0.05016900	2.11736400

C	-5.40556500	-2.59794400	0.37344500
C	-5.09455400	1.14681000	-2.68075900
C	-5.19163000	1.78363900	2.14752700
C	6.00217600	-2.51793500	1.84362400
C	5.90832200	-0.13489800	2.24741000
C	8.04570900	-1.20750800	1.89161200
C	-6.21986300	-3.26624500	-0.54346300
C	-5.96083700	-2.09055800	1.55319100
C	-5.75989300	2.34526400	-2.94502100
C	-5.76216000	-0.07129500	-2.84633900
C	-5.99174700	1.43065000	3.23598100
C	-5.72768100	2.55651200	1.11242200
H	5.49140000	-3.46362500	1.71062300
H	5.32409900	0.75852100	2.42833600
H	9.12478400	-1.17760600	1.80902100
H	-5.79550700	-3.67020900	-1.45488000
C	-7.58760300	-3.43926800	-0.26450300
H	-5.32073000	-1.56937400	2.25327400
C	-7.32562200	-2.22027400	1.81019500
H	-5.24810200	3.29251300	-2.82115300
C	-7.08595500	2.31509900	-3.41251500
H	-5.23620400	-0.99216400	-2.62982800
C	-7.09674300	-0.10109200	-3.24650500
H	-5.58107600	0.83689400	4.04377600
C	-7.32531500	1.87172800	3.29240300
H	-5.09872700	2.81661300	0.27064100
C	-7.06318700	2.95873000	1.14472400
C	-8.14280000	-2.89427400	0.90114700
C	-8.43099200	-4.22587300	-1.20219400
C	-7.91459600	-1.60958100	3.03542300
C	-7.76130000	1.09335900	-3.53275400
C	-7.76089400	3.59489500	-3.78321000
C	-7.81819100	-1.40306300	-3.33637800
C	-7.86473700	2.61775100	2.23585700
C	-8.14483900	1.55206800	4.49055800
C	-7.64028200	3.72892700	0.00492000
H	-9.20079300	-2.99808400	1.11000200
O	-9.62422300	-4.55460000	-0.69328100
O	-8.09409000	-4.57306400	-2.33127000
O	-6.99659400	-1.43388900	3.99891800
O	-9.08632700	-1.28873000	3.14272100
H	-8.80541800	1.04908000	-3.82825400
O	-8.66594800	3.55694800	-4.77141000
O	-7.53705700	4.67264800	-3.24695500
O	-6.96974300	-2.42965100	-3.49245300
O	-9.02951200	-1.51819300	-3.26102400
H	-8.89910100	2.93940700	2.25776700
O	-9.28431200	2.25066900	4.55620300
O	-7.82934800	0.74508600	5.36091900
O	-6.68715300	4.30550300	-0.73913400
O	-8.83506700	3.81237600	-0.22583700
H	0.15617100	4.75826700	2.70683100
H	-0.44656800	-4.87340900	3.18206400
H	-0.46543500	-5.31640000	-2.11779900
H	-0.01664800	-0.55767700	-5.50452500
H	1.06329100	-2.12871600	-2.50740800
H	1.02613100	-1.65726300	3.00963900
H	1.27943100	2.97749300	-0.16359800
H	7.58544000	1.15420000	-2.68682500
H	9.25421300	-5.97102200	-1.49626200

H	8.16449900	-5.51177600	1.27936500
H	9.22023100	0.96484400	0.87263100
H	7.51003100	3.79480300	2.94526100
H	8.55602500	4.77354500	-4.19887900
H	-8.66775800	2.69516300	-5.22453300
H	-7.07375000	4.61451400	-1.60173100
H	-9.76678000	1.95925700	5.35448400
H	-7.36327700	-0.81972700	4.68364500
H	-10.11688000	-5.04354300	-1.38115900
H	-7.44430000	-3.27977600	-3.30756400
H	-0.23701800	0.41424400	5.64536700
O	-1.32008400	3.81732400	-1.24078900
H	-2.15578600	3.91340500	-1.73415700
H	-1.25865300	4.55737000	-0.60838300
C	1.58399700	2.65353100	-2.85929900
H	0.84497300	3.49685700	-2.70121600
O	2.47632200	2.86132400	-3.68387900

Sum of electronic and thermal Free Energies = -6804.876486 (Hartree/Particle).

Supplementary Table 22. DFT-optimized geometry of f-$[Zr^{IV}(BTC^{\cdot-})(BTC)(H_2O)(OCH_2)]^-$ (doublet), computed at the UB3LYP-D3/ LANL2DZ (Zr)/ 6-31G* (HCO) level in water solvent using PCM. The spin density plot is also shown below. (Isovalue = 0.0004)

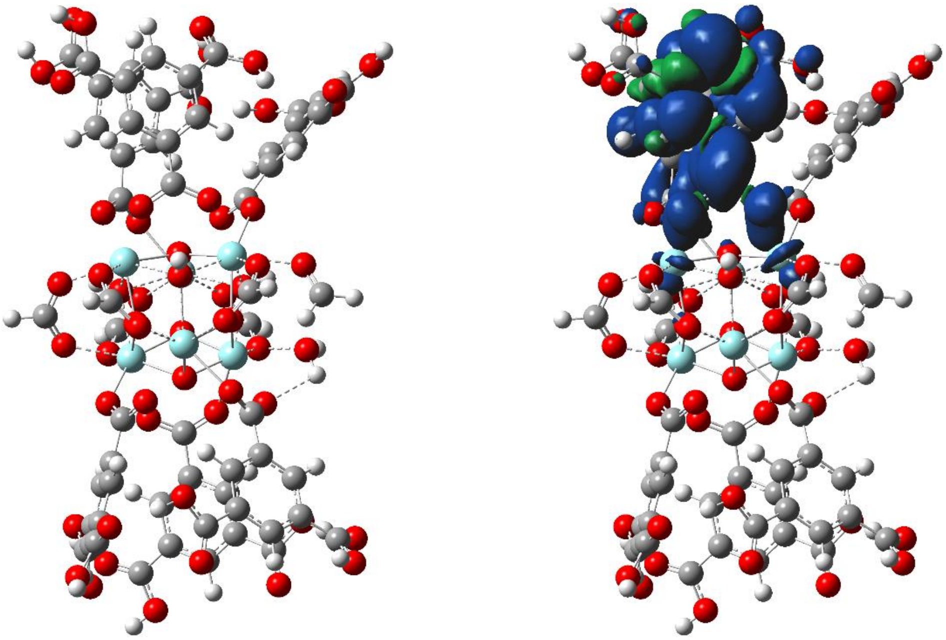

Spin Density Distribution

Atom	x	y	z
O	8.16554800	1.31870400	-1.89616000
O	7.16140800	3.35586100	-3.68208300
O	8.30414800	5.13454500	-2.89591100
O	9.36098500	-0.02286700	-0.61793500
C	8.39955200	0.09688200	-1.38548200
C	7.42792300	4.12398600	-2.76986700

C	7.49578000	-1.02181900	-1.70037800
C	6.83318900	4.02484400	-1.41055100
C	6.13422200	-0.79602700	-1.88083700
C	8.00914400	-2.34666600	-1.60988900
C	5.71950800	3.19910300	-1.25321300
C	7.36999400	4.68135800	-0.30099600
H	5.72902700	0.21152900	-1.90634100
C	5.21434700	-1.87593900	-1.86595700
H	9.07003100	-2.50798800	-1.48173300
C	7.10419000	-3.42374900	-1.68403600
H	5.28152200	2.72049900	-2.12013200
C	5.15714400	2.99052900	0.00769000
H	8.21161000	5.35507300	-0.41087100
C	6.84257400	4.44292400	0.97278700
C	5.73517400	-3.19709500	-1.74842600
C	3.79393800	-1.65020400	-1.87088400
C	7.55941600	-4.83723000	-1.68696500
C	5.74223000	3.58920800	1.13120900
C	3.92105300	2.14532300	0.09625200
C	7.45797800	5.18617900	2.12621300
H	5.05371400	-4.03858800	-1.75096800
O	3.01402900	-2.59095500	-1.41325100
O	3.31477600	-0.52654300	-2.32565800
O	8.90653800	-4.96109100	-1.59385200
O	6.83203900	-5.81301800	-1.77855800
H	5.32203000	3.40269900	2.11284200
O	3.42072900	1.80040900	-1.01738000
O	3.44911100	1.84941300	1.23660700
O	7.59392100	4.52794700	3.27952000
O	7.81223700	6.34726700	2.01759100
Zr	1.36002600	-2.42515800	-0.01838800
Zr	1.62686200	0.68955700	-1.72419000
Zr	1.55838700	0.68990500	1.84597200
Zr	-1.49063000	-1.03104600	-1.70184000
Zr	-1.49993400	-1.17043800	1.70930900
O	3.20146100	-2.53937400	1.33716600
O	0.75530800	-1.37021300	-1.96371000
O	-0.65793100	-1.94527800	-0.01861600
O	0.76634600	-1.46753800	1.98298500
O	1.84892400	-0.34710500	0.05545400
O	0.65544300	-4.08593700	1.35701400
O	0.56656900	-4.03498500	-1.39896700
Zr	-1.33642000	1.78717300	0.11119500
O	-0.47390800	0.80935200	-1.52858000
O	0.96773400	2.06284400	0.00935500
O	0.97946000	0.48829700	-3.89888800
O	7.66491100	-4.92914200	1.27703100
O	3.24316400	-0.59429000	2.48572700
O	-0.48179600	0.66734400	1.61193000
O	1.13023800	2.75957900	2.64650600
O	0.93936600	0.34247200	3.99376700
O	7.37024800	1.90114700	3.03711800
O	9.00779700	1.49885400	1.59303400
O	9.57185300	-3.73495700	1.41423600
O	-3.46233300	-2.17012600	-1.21482100
O	-3.06653600	0.32035400	-2.53973900
O	-2.28606000	-0.05588300	0.02729400
O	-0.97188900	-0.68570300	-3.92946600
O	-1.22433100	-3.15828200	-2.49703700
O	-3.30615200	-0.00727800	2.59046900

O	-3.23768700	-2.37017800	1.02151800
O	-1.16867900	-3.30111100	2.46586200
O	-1.01005500	-0.83852100	3.94011300
C	3.81211300	-1.57996000	1.90095100
C	-0.32060800	-4.18409400	2.15918500
C	-0.41679000	-4.08178000	-2.19556000
O	-3.12074400	2.22596100	-1.33941400
O	-3.01141400	1.99033900	1.58927100
O	-0.72804000	3.50467400	1.57233700
C	-0.01847200	-0.05853300	-4.46467600
C	8.35421100	-3.77583900	1.42056000
C	0.19694400	3.58776700	2.42763100
C	-0.07428700	-0.21656400	4.51497200
C	7.90559200	1.13150400	2.23646300
C	-3.91645400	-2.37720800	-0.05318000
C	-3.64786000	1.35262900	-2.09648700
C	-3.71712200	1.11923800	2.18687400
C	5.29988400	-1.55384600	1.87944800
C	7.47059400	-2.59399700	1.60149000
C	7.31880200	-0.21082500	1.99294000
C	-5.38360800	-2.63729400	0.13440900
C	-5.08511900	1.49000600	-2.50589200
C	-5.15709600	1.49257000	2.38145000
C	6.07320500	-2.69819300	1.62265400
C	5.92798900	-0.32561800	2.08894300
C	8.09365300	-1.35133400	1.75688200
C	-6.20592900	-3.18807500	-0.85107400
C	-5.92311700	-2.30104400	1.38035300
C	-5.75201200	2.71150200	-2.61530900
C	-5.76008100	0.29931400	-2.79638300
C	-5.95024200	1.00043900	3.42013800
C	-5.70310700	2.38541200	1.45412100
H	5.58696400	-3.65107200	1.45929400
H	5.32641200	0.55228400	2.28737700
H	9.17383000	-1.29935000	1.70923900
H	-5.79276600	-3.45689400	-1.81587200
C	-7.56564500	-3.41847800	-0.57573700
H	-5.27692400	-1.86708700	2.13244700
C	-7.28140500	-2.48501300	1.63946400
H	-5.23630900	3.63783900	-2.39020600
C	-7.08628600	2.73634300	-3.05901100
H	-5.23356200	-0.64092900	-2.69522300
C	-7.10162000	0.31701300	-3.17299000
H	-5.53228600	0.31298200	4.14571600
C	-7.28701200	1.41997400	3.53552000
H	-5.07950000	2.75652200	0.65108400
C	-7.04182600	2.76884100	1.53950300
C	-8.10601500	-3.04489600	0.66213300
C	-8.41694300	-4.08066400	-1.59844400
C	-7.85826600	-2.04440400	2.94068400
C	-7.76673500	1.53754400	-3.30977600
C	-7.76557500	4.05142000	-3.25939200
C	-7.83059400	-0.96471000	-3.39799300
C	-7.83690300	2.28639900	2.58082000
C	-8.09722100	0.95066900	4.69023600
C	-7.62762300	3.67216200	0.50686500
H	-9.15873600	-3.19107800	0.87233600
O	-9.58709000	-4.51291200	-1.11410600
O	-8.10549000	-4.24793600	-2.77490400
O	-6.92399300	-1.95973600	3.90130300

O	-9.03474700	-1.77002400	3.10774600
H	-8.81577100	1.52891600	-3.59098000
O	-8.67806000	4.13827200	-4.23734300
O	-7.53874400	5.05164600	-2.59056600
O	-6.98832800	-1.97434500	-3.65914400
O	-9.04275600	-1.07909700	-3.33625000
H	-8.87373900	2.59395200	2.64540800
O	-9.26084000	1.59917200	4.82130300
O	-7.75533900	0.07057000	5.47532400
O	-6.68044300	4.34864100	-0.15647300
O	-8.82326700	3.77249000	0.28760100
H	0.20534000	4.49459500	3.05246300
H	-0.43487400	-5.16049800	2.65574700
H	-0.58047900	-5.04968200	-2.69561300
H	-0.04501700	0.02506400	-5.56280500
H	1.09157200	-1.84060100	-2.74190200
H	1.08378400	-1.94640100	2.76311800
H	1.34500200	2.95533600	0.04804400
H	7.48378000	1.32251400	-2.59312000
H	9.10561100	-5.91574400	-1.63238100
H	8.32006600	-5.64003700	1.14362100
H	9.22095300	0.90718000	0.81894100
H	7.51963900	3.53790200	3.15825500
H	8.66647800	5.10167800	-3.80250400
H	-8.67977100	3.34365600	-4.80000600
H	-7.07097300	4.76751500	-0.96945700
H	-9.73275800	1.21124400	5.58398800
H	-7.28851900	-1.43351800	4.65576600
H	-10.08866500	-4.90783800	-1.85405300
H	-7.46699100	-2.83778300	-3.56814500
H	-0.13491300	-0.15614600	5.61325100
O	-1.11283000	3.89797400	-1.08077500
H	-1.98957300	4.08761700	-1.46630500
H	-0.91841000	4.61537500	-0.44912800
C	0.40413000	3.32564600	-3.12425200
O	1.43246700	2.89520300	-2.61445700
H	-0.46399600	2.67593400	-3.28172100
H	0.34990500	4.37059500	-3.45210100

Sum of electronic and thermal Free Energies = -6805.468589 (Hartree/Particle).

Supplementary Table 23. DFT-optimized geometry of f-[Zr^{IV}($BTC^{\cdot-}$)(BTC)(H_2O)(OCH_3)]$^-$ (singlet), computed at the B3LYP-D3/ LANL2DZ (Zr)/ 6-31G* (HCO) level in water solvent using PCM.

Atom	x	y	z
O	8.27225300	1.17607000	-1.95824300
O	7.22768800	2.95383700	-3.85059800
O	8.22009200	4.89975900	-3.28987500
O	9.36330000	-0.08852800	-0.52388500
C	8.45713700	0.00853700	-1.34660700
C	7.42878400	3.84670400	-3.03802200
C	7.53047600	-1.12627700	-1.61672100
C	6.83781100	3.86053700	-1.67417100

C	6.15321400	-0.91964800	-1.76073600
C	8.04760100	-2.42653100	-1.56007400
C	5.74002800	3.03097000	-1.43983800
C	7.36258300	4.62627400	-0.63105100
H	5.72432600	0.07767700	-1.76890400
C	5.28863300	-2.02063600	-1.77895800
H	9.11126000	-2.57343200	-1.41924400
C	7.18623100	-3.52223000	-1.65968600
H	5.30766700	2.46929100	-2.25849300
C	5.17605000	2.93250400	-0.16676400
H	8.19165400	5.30243600	-0.80365600
C	6.83800100	4.49589800	0.65979500
C	5.80470000	-3.31500300	-1.72940500
C	3.80721500	-1.79483200	-1.72870500
C	7.68016300	-4.92829800	-1.63617200
C	5.74869100	3.64484900	0.89536400
C	3.94438100	2.08233800	-0.00743500
C	7.43713700	5.35568000	1.73817300
H	5.13552200	-4.16743900	-1.73226800
O	3.13786600	-2.63508900	-1.05191500
O	3.35052500	-0.77808300	-2.32750300
O	9.01743400	-5.00864600	-1.48166000
O	6.96390100	-5.90624000	-1.74065400
H	5.32505000	3.54909800	1.88878700
O	3.51844700	1.53859200	-1.06271900
O	3.41621800	1.98783700	1.14719400
O	7.56214700	4.82201000	2.95693400
O	7.78700800	6.50153600	1.51710100
Zr	1.35144400	-2.34455700	0.31140300
Zr	1.58759300	0.56683100	-1.82318800
Zr	1.53428900	0.88921200	1.79986800
Zr	-1.48512000	-1.26197200	-1.54954800
Zr	-1.52768200	-0.97847300	1.87284400
O	3.18611200	-2.36706800	1.68599700
O	0.77682400	-1.58099600	-1.74396100
O	-0.65835300	-1.95940800	0.25310300
O	0.74207500	-1.23010600	2.19855000
O	1.85041900	-0.32444300	0.15378000
O	0.68974800	-3.89290600	1.84995000
O	0.71885500	-4.15140700	-0.90660900
Zr	-1.33610600	1.75011200	-0.08107500
O	-0.50443000	0.60948000	-1.61302400
O	0.94356400	2.03416800	-0.15830300
O	1.02013000	-0.13409400	-3.95719100
O	7.55652300	-4.75549000	1.42826000
O	3.22885400	-0.31408500	2.62462200
O	-0.51256100	0.83086000	1.56476700
O	1.10627700	3.01810400	2.41006500
O	0.90680200	0.75348700	3.99095400
O	7.40823900	2.15917600	3.01154900
O	9.02710800	1.64018200	1.58780900
O	9.48891200	-3.59277800	1.48121700
O	-3.44502200	-2.35916000	-0.93386800
O	-3.09262600	-0.07629100	-2.53291600
O	-2.28761100	-0.09553500	0.05396800
O	-0.98301400	-1.19661900	-3.77477700
O	-1.14909200	-3.48985400	-2.02840300
O	-3.32459800	0.32602600	2.55149600
O	-3.25641900	-2.27286400	1.31214200
O	-1.15966600	-3.02845100	2.85736000

O	-1.08407900	-0.35689200	4.03328800
C	3.78871700	-1.35296200	2.14956600
C	-0.29455600	-3.92401800	2.64744000
C	-0.28906200	-4.33444900	-1.65449900
O	-3.15836100	1.97087900	-1.58665600
O	-3.02994500	2.17164100	1.28759800
O	-0.74922100	3.64469600	1.25909300
C	-0.00723500	-0.70672400	-4.41557400
C	8.27446600	-3.61906800	1.55478600
C	0.17638300	3.82042300	2.09579500
C	-0.13073600	0.29152000	4.55212100
C	7.93056800	1.32967400	2.27004600
C	-3.92062800	-2.40314300	0.23658800
C	-3.67267800	1.00989800	-2.23206100
C	-3.73361000	1.38927400	2.00140400
C	5.28422400	-1.33473500	2.10569400
C	7.42378100	-2.41781000	1.78119600
C	7.32348000	-0.02372300	2.12278600
C	-5.39733000	-2.59948700	0.43581900
C	-5.10416000	1.09497900	-2.68039300
C	-5.16978300	1.79690500	2.14817700
C	6.02823400	-2.49244500	1.85695800
C	5.93365200	-0.10832600	2.25276700
C	8.07143000	-1.18164200	1.90084900
C	-6.22303700	-3.27248200	-0.46755200
C	-5.94128700	-2.07247200	1.61235500
C	-5.76541300	2.29327500	-2.95542700
C	-5.77874100	-0.12137400	-2.82843300
C	-5.96024800	1.46623900	3.25036300
C	-5.71283700	2.55564200	1.10632000
H	5.51741900	-3.43845500	1.72659800
H	5.34854500	0.78546100	2.42912700
H	9.15045500	-1.15180000	1.81826500
H	-5.80800300	-3.69117000	-1.37666600
C	-7.59051500	-3.43056100	-0.17822000
H	-5.29256600	-1.54754700	2.30167900
C	-7.30569800	-2.18718800	1.87891500
H	-5.24902500	3.23957200	-2.84374000
C	-7.09375700	2.26396000	-3.41689400
H	-5.25616300	-1.04184800	-2.60271700
C	-7.11496300	-0.14990300	-3.22306400
H	-5.54399900	0.88399200	4.06359900
C	-7.29109800	1.91481800	3.31315900
H	-5.09123800	2.79868000	0.25406900
C	-7.04539100	2.96673000	1.14625400
C	-8.13398700	-2.86552500	0.98336400
C	-8.44633300	-4.22363300	-1.09901900
C	-7.88346300	-1.55707500	3.09958300
C	-7.77518100	1.04409400	-3.52110100
C	-7.76545000	3.54258600	-3.79726000
C	-7.84210300	-1.44980700	-3.29248400
C	-7.83744700	2.64761000	2.25092200
C	-8.10012000	1.61590600	4.52360800
C	-7.62869100	3.72390700	0.00086600
H	-9.19149400	-2.95749700	1.19993500
O	-9.64351300	-4.52511200	-0.58218700
O	-8.11618700	-4.59863300	-2.22117100
O	-6.96003800	-1.37814700	4.05719500
O	-9.05199200	-1.22492200	3.20827400
H	-8.82064400	1.00134300	-3.81203200

O	-8.67253700	3.49894500	-4.78340900
O	-7.53778700	4.62425400	-3.27048000
O	-6.99792200	-2.48206700	-3.43503000
O	-9.05367400	-1.55925200	-3.21234100
H	-8.86968500	2.97561600	2.27863200
O	-9.23492400	2.32186900	4.59177400
O	-7.78049800	0.81813100	5.40098500
O	-6.67857200	4.27955600	-0.76276100
O	-8.82518300	3.81508400	-0.21785900
H	0.19087800	4.79217900	2.61589500
H	-0.40118600	-4.84789800	3.23859300
H	-0.42142900	-5.36116500	-2.03211700
H	-0.05877900	-0.80227700	-5.51289800
H	1.08719900	-2.14151200	-2.47027000
H	1.05859400	-1.62420600	3.02488500
H	1.27493900	2.93411000	-0.30395400
H	7.61453100	1.17239300	-2.68243600
H	9.25854300	-5.95488000	-1.48930100
H	8.18916400	-5.48626400	1.28950700
H	9.24488700	0.98611200	0.87531000
H	7.50364500	3.82721300	2.94237800
H	8.59272900	4.78674100	-4.18607100
H	-8.67804800	2.63306300	-5.22861900
H	-7.07055000	4.57939500	-1.62610800
H	-9.71101100	2.04317000	5.39837200
H	-7.31970500	-0.75381200	4.73657000
H	-10.14356900	-5.02114400	-1.25951900
H	-7.47420600	-3.32707200	-3.23340800
H	-0.20634700	0.47234100	5.63656800
O	-1.33111300	3.80057000	-1.25774100
H	-2.16327500	3.89143000	-1.75740100
H	-1.29567800	4.51846400	-0.59759200
C	0.67258100	2.80597200	-3.79016600
H	-0.35624600	2.68601300	-3.42134900
H	0.85315200	3.87844300	-3.95405100
H	0.74674700	2.28863100	-4.75789500
O	1.60341100	2.29344600	-2.86772000

Sum of electronic and thermal Free Energies = -6806.124823 (Hartree/Particle).

Supplementary Table 24. DFT-optimized geometry of **f**-[Zr^{IV}($BTC^{\cdot -}$)(BTC)(H_2O)(CH_3OH)]$^-$ (doublet), computed at the UB3LYP-D3/ LANL2DZ (Zr)/ 6-31G* (HCO) level in water solvent using PCM. The spin density plot is also shown below. (Isovalue = 0.0004)

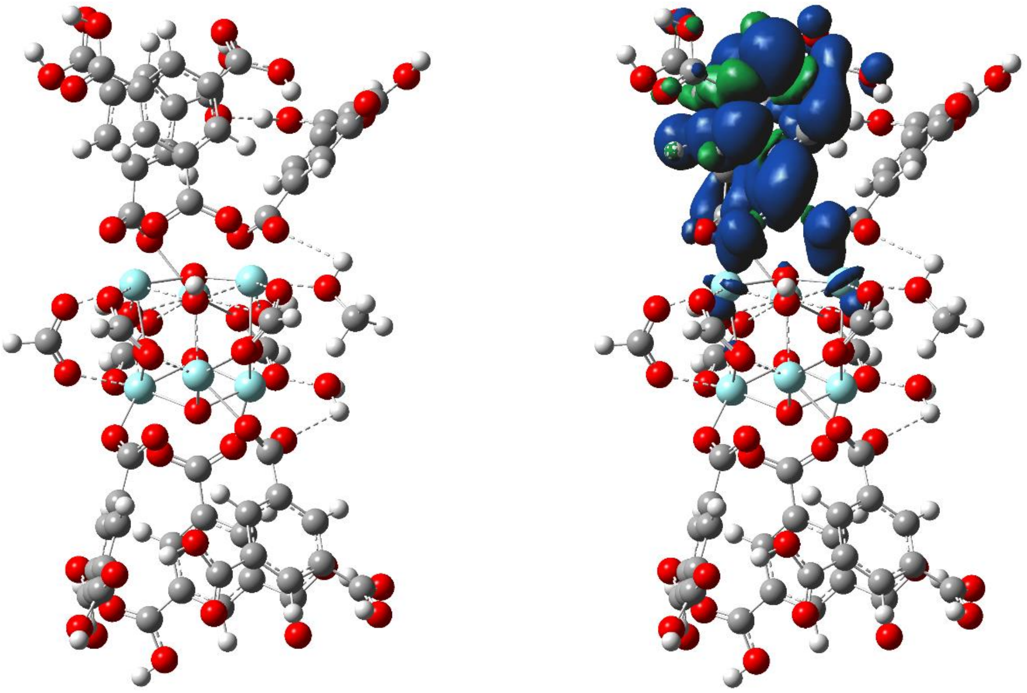

Spin Density Distribution

Atom	x	y	z
O	8.13056600	1.35468600	-1.85985600
O	7.14216700	3.41820400	-3.65142700
O	8.27820100	5.19215600	-2.84440300
O	9.35581000	-0.00273100	-0.62550400
C	8.38223700	0.12248400	-1.37871700
C	7.40444500	4.17790900	-2.73106500
C	7.48711200	-0.99675400	-1.70491700
C	6.80708800	4.06249300	-1.37426800
C	6.12220900	-0.78187600	-1.87875600
C	8.00893900	-2.32142800	-1.63072400
C	5.69729700	3.22940800	-1.22827300
C	7.34026700	4.70697800	-0.25600400
H	5.70848900	0.22253200	-1.89418400
C	5.21235900	-1.86707200	-1.87390100
H	9.07164700	-2.47675400	-1.51044700
C	7.11262700	-3.40303100	-1.71411900
H	5.26449300	2.75831600	-2.10197900
C	5.13626500	3.00040600	0.02937800
H	8.17887100	5.38590200	-0.35662000
C	6.81436300	4.44788700	1.01438400
C	5.74076900	-3.18679900	-1.77094500
C	3.78825600	-1.64665700	-1.86958400
C	7.57929300	-4.81314500	-1.73552000
C	5.71908100	3.58584500	1.16100200
C	3.90696100	2.14418400	0.10982500
C	7.42712800	5.17599700	2.17884400
H	5.06505100	-4.03270500	-1.78119800
O	3.01677000	-2.59489600	-1.42011500
O	3.30683300	-0.52068300	-2.30904300
O	8.92735900	-4.92802700	-1.64762000
O	6.85898500	-5.79292400	-1.83787200
H	5.30111100	3.38180100	2.14003700

O	3.39549600	1.82759600	-1.01038700
O	3.45424400	1.81217800	1.24553900
O	7.57020300	4.49895800	3.32014100
O	7.77338500	6.34113800	2.08901000
Zr	1.35430300	-2.44656100	-0.02166100
Zr	1.60403200	0.68441200	-1.70796100
Zr	1.55555500	0.64575800	1.86281100
Zr	-1.49720500	-1.04536300	-1.69074700
Zr	-1.50718500	-1.20620800	1.72387500
O	3.20041300	-2.57093600	1.32566300
O	0.74549300	-1.38246600	-1.95542800
O	-0.66498600	-1.97187400	-0.01113000
O	0.76239900	-1.51020400	1.98790500
O	1.84542900	-0.37185700	0.06308100
O	0.65397900	-4.12149700	1.33804600
O	0.56586600	-4.04681700	-1.41674600
Zr	-1.32151600	1.76051800	0.14181000
O	-0.48289700	0.80064600	-1.52175500
O	0.95547500	2.02466100	0.06350300
O	0.99203500	0.46684400	-3.88623300
O	7.68482400	-4.93831900	1.22275000
O	3.24173400	-0.63878600	2.49470800
O	-0.48499200	0.62876900	1.64363600
O	1.18661500	2.68701600	2.72769600
O	0.94429000	0.27032000	4.01650100
O	7.34967700	1.87747500	3.04026000
O	8.99445200	1.49048000	1.59994500
O	9.58438200	-3.73450400	1.37415900
O	-3.46991800	-2.19442400	-1.19510500
O	-3.07714900	0.29362800	-2.51760800
O	-2.28154700	-0.07903200	0.04340200
O	-0.97765600	-0.67747300	-3.92173200
O	-1.22971200	-3.16573000	-2.50490100
O	-3.29587900	0.03001800	2.58447200
O	-3.25761300	-2.37914500	1.04409000
O	-1.17167200	-3.34606800	2.45119600
O	-1.02577000	-0.87590100	3.94846900
C	3.81241600	-1.61567500	1.89591500
C	-0.32353800	-4.22647900	2.13748600
C	-0.41840900	-4.08918700	-2.21274800
O	-3.12362000	2.20159900	-1.31896400
O	-2.99828300	2.01862400	1.56380600
O	-0.68405800	3.46045200	1.69291200
C	-0.02018700	-0.05169400	-4.45215100
C	8.36676200	-3.78197000	1.37889400
C	0.25529000	3.52684900	2.53028200
C	-0.07917000	-0.27482500	4.53030000
C	7.89096700	1.11434000	2.23656900
C	-3.93102900	-2.38963800	-0.03515000
C	-3.65187400	1.33212600	-2.07552800
C	-3.70542800	1.15106300	2.17148400
C	5.29935300	-1.58293500	1.86623800
C	7.47585400	-2.60780500	1.57138000
C	7.31143200	-0.22848800	1.98308200
C	-5.40019800	-2.63966000	0.14914500
C	-5.08749700	1.47476700	-2.49263200
C	-5.14466500	1.52898400	2.36162000
C	6.07867700	-2.72132400	1.59626900
C	5.92146000	-0.35313600	2.08347900
C	8.09222100	-1.36315600	1.73555300

C	-6.22280200	-3.19466900	-0.83366200
C	-5.94128300	-2.29013300	1.39055100
C	-5.74637200	2.69901800	-2.61758200
C	-5.76798600	0.28587900	-2.77746400
C	-5.94105300	1.04073200	3.39998700
C	-5.68760300	2.41827600	1.42905400
H	5.59787500	-3.67605300	1.42813500
H	5.31571600	0.51970900	2.29090600
H	9.17193500	-1.30469500	1.68578900
H	-5.80885400	-3.47373000	-1.79522800
C	-7.58415500	-3.41660800	-0.55923800
H	-5.29507900	-1.85302100	2.14064000
C	-7.30068600	-2.46589500	1.64856600
H	-5.22597500	3.62422100	-2.39878100
C	-7.07811100	2.72802700	-3.06917900
H	-5.24728100	-0.65651400	-2.66677800
C	-7.10699700	0.30807000	-3.16282700
H	-5.52563000	0.35535400	4.12910400
C	-7.27788600	1.46142400	3.51015700
H	-5.06167300	2.78462700	0.62554800
C	-7.02672900	2.80170100	1.50905800
C	-8.12571500	-3.03066000	0.67438500
C	-8.43605500	-4.08300900	-1.57876200
C	-7.87582900	-2.01146300	2.94573300
C	-7.76439800	1.53111800	-3.31319700
C	-7.74749300	4.04524700	-3.28742900
C	-7.84044800	-0.97145700	-3.38550200
C	-7.82488100	2.32348500	2.54981200
C	-8.09162900	0.99943200	4.66539900
C	-7.61010200	3.69941700	0.47035000
H	-9.17946900	-3.17100900	0.88346400
O	-9.60622300	-4.51255700	-1.09216900
O	-8.12488700	-4.25543800	-2.75453900
O	-6.94057500	-1.92372700	3.90523900
O	-9.05079500	-1.72961200	3.11068200
H	-8.81173700	1.52606100	-3.60077100
O	-8.65764900	4.12649500	-4.26818100
O	-7.51467800	5.05261100	-2.63139900
O	-7.00106100	-1.98512400	-3.64066400
O	-9.05325000	-1.08116900	-3.32782600
H	-8.86196300	2.63109400	2.60981200
O	-9.25817300	1.64483800	4.78542900
O	-7.75020300	0.12782400	5.46009300
O	-6.66205900	4.37913200	-0.18832100
O	-8.80473300	3.79239300	0.24235100
H	0.28503100	4.42576000	3.16661100
H	-0.43944800	-5.20765400	2.62428600
H	-0.57919700	-5.05282800	-2.72189000
H	-0.05578500	0.05541800	-5.54824700
H	1.07777800	-1.84240700	-2.74135100
H	1.08644100	-1.99273700	2.76305200
H	1.29998000	2.93092100	0.04881100
H	7.44868400	1.36487200	-2.55617100
H	9.13347200	-5.88061100	-1.69825400
H	8.34500600	-5.64329500	1.08297900
H	9.21089800	0.90697400	0.81984800
H	7.49940400	3.51034800	3.18276400
H	8.64315600	5.16970500	-3.75025200
H	-8.66414700	3.32502300	-4.82096200
H	-7.04933700	4.78965500	-1.00723200

H	-9.73187800	1.26197600	5.54956100
H	-7.30095400	-1.38912100	4.65554600
H	-10.10835400	-4.91023100	-1.83028400
H	-7.48308300	-2.84660800	-3.54944100
H	-0.14287700	-0.22306500	5.62896300
O	-1.20913300	3.91463900	-0.83336700
H	-2.03599500	4.09909000	-1.31597500
H	-1.12927600	4.57519000	-0.11978200
C	0.52897100	3.36909300	-3.48030000
H	0.40769000	4.44220900	-3.31842500
H	0.82611000	3.15590600	-4.50976900
H	-0.39858700	2.85095300	-3.24397400
O	1.51148400	2.85458600	-2.55515600
H	2.36389500	3.31278300	-2.64504200

Sum of electronic and thermal Free Energies = -6806.671219 (Hartree/Particle).

Supplementary Table 25. DFT-optimized geometry of f-[Zr^{IV}($BTC^{\cdot-}$)(BTC)(H_2O)(CH_3)]$^{-}$ (singlet), computed at the B3LYP-D3/ LANL2DZ (Zr)/ 6-31G* (HCO) level in water solvent using PCM.

Atom	x	y	z
O	8.25249300	1.11910300	-2.02621300
O	7.21234200	2.83418000	-3.97975600
O	8.20796700	4.79831100	-3.49331800
O	9.35334500	-0.09572200	-0.55715300
C	8.44342000	-0.02789800	-1.37872800
C	7.41762000	3.75513400	-3.20016900
C	7.51897300	-1.17376700	-1.60726300
C	6.83340900	3.81770000	-1.83492800
C	6.14079700	-0.97674200	-1.75725900
C	8.04042900	-2.46945100	-1.50350900
C	5.74048000	2.99288600	-1.56453600
C	7.35973600	4.62401400	-0.82345500
H	5.70789300	0.01813000	-1.80014700
C	5.28037800	-2.08129800	-1.73391300
H	9.10476300	-2.60749300	-1.35886400
C	7.18290400	-3.57108800	-1.56089500
H	5.30534000	2.39940400	-2.35874300
C	5.18200500	2.94015100	-0.28650100
H	8.18579600	5.29591400	-1.02481400
C	6.84057800	4.54042400	0.47343200
C	5.80069400	-3.37123500	-1.63626100
C	3.79878200	-1.85949900	-1.68744700
C	7.68185500	-4.97353400	-1.48470300
C	5.75477200	3.69558100	0.74516200
C	3.95416600	2.09175000	-0.09363700
C	7.44149700	5.44049000	1.51721700
H	5.13438800	-4.22538500	-1.60559500
O	3.13411800	-2.67398200	-0.97535600
O	3.33513500	-0.86686100	-2.32231200
O	9.02008600	-5.04312200	-1.33399400
O	6.96848700	-5.95720000	-1.54669300
H	5.33489300	3.63665300	1.74304900

O	3.55269700	1.47344900	-1.11482800
O	3.40137700	2.07732000	1.05533100
O	7.57802900	4.94952800	2.75273700
O	7.78329700	6.58000000	1.25471900
Zr	1.34809300	-2.32384500	0.37104200
Zr	1.59498500	0.49814700	-1.84006700
Zr	1.52856500	0.97641500	1.73384200
Zr	-1.48179800	-1.30412900	-1.52771300
Zr	-1.53004200	-0.88794500	1.87644300
O	3.18753200	-2.26799200	1.73111900
O	0.77238900	-1.65779800	-1.72072000
O	-0.65899600	-1.93195900	0.29816400
O	0.74055200	-1.12651000	2.21279100
O	1.85518300	-0.30693500	0.14492700
O	0.70040700	-3.80047100	1.96934500
O	0.69466300	-4.17850800	-0.76350200
Zr	-1.34316700	1.77369600	-0.18017900
O	-0.48800900	0.55486100	-1.63644600
O	0.95742300	2.05207000	-0.27919200
O	1.00641800	-0.16830800	-3.96360500
O	7.54512100	-4.67931900	1.57618500
O	3.23096000	-0.18248100	2.59989700
O	-0.51325100	0.90961200	1.49434500
O	1.05808200	3.13693700	2.25344400
O	0.92154200	0.92792800	3.92307600
O	7.42541600	2.28902000	2.89983900
O	9.03654000	1.71311800	1.48893800
O	9.48164200	-3.52227100	1.58793900
O	-3.44844400	-2.36862100	-0.87732300
O	-3.07765000	-0.13661800	-2.56707000
O	-2.29031600	-0.07582400	0.02772000
O	-0.95825500	-1.30171600	-3.75142200
O	-1.16259200	-3.55370500	-1.92560500
O	-3.32674800	0.41467800	2.52407800
O	-3.25691600	-2.20402500	1.36446900
O	-1.15299200	-2.89963000	2.93576100
O	-1.07192500	-0.17563800	4.01369900
C	3.78913300	-1.23901300	2.16172400
C	-0.28628500	-3.80115300	2.76567400
C	-0.30892100	-4.38614700	-1.51108100
O	-3.14291600	1.93977200	-1.68528300
O	-3.03511000	2.21482400	1.19740800
O	-0.76870000	3.70736500	1.02709200
C	0.00523700	-0.80174200	-4.40296000
C	8.26693600	-3.54129400	1.65919300
C	0.13342500	3.91913600	1.88478400
C	-0.11614500	0.48873700	4.50490300
C	7.94148200	1.43185900	2.18602500
C	-3.92240400	-2.37209200	0.29471300
C	-3.65815400	0.95724600	-2.30062600
C	-3.73780800	1.45850800	1.93760800
C	5.28469700	-1.22652300	2.11927600
C	7.42035600	-2.32900100	1.83691000
C	7.32860800	0.07649600	2.08881000
C	-5.39889300	-2.56245100	0.50020100
C	-5.08890000	1.03110200	-2.75141000
C	-5.17387700	1.87072600	2.07212700
C	6.02456900	-2.39546100	1.91401600
C	5.93848800	0.00193500	2.22195800
C	8.07237700	-1.09152200	1.91020000

C	-6.22472200	-3.25717800	-0.38662400
C	-5.94324200	-2.00448100	1.66208000
C	-5.74874500	2.22191700	-3.06063600
C	-5.76453100	-0.18840200	-2.86476600
C	-5.96482800	1.57583100	3.18408500
C	-5.71591400	2.59783200	1.00743600
H	5.51021800	-3.34366100	1.81829700
H	5.35665900	0.90366100	2.36647300
H	9.15147700	-1.06847200	1.82648600
H	-5.80917700	-3.69957100	-1.28419600
C	-7.59297900	-3.40464900	-0.09577700
H	-5.29430000	-1.46349400	2.33864300
C	-7.30848100	-2.10898300	1.92895300
H	-5.23186700	3.17060300	-2.97418800
C	-7.07606200	2.18045300	-3.52399100
H	-5.24366700	-1.10246400	-2.61087700
C	-7.10007400	-0.22688800	-3.26068600
H	-5.54962600	1.01864600	4.01520400
C	-7.29497500	2.02845300	3.23280000
H	-5.09379100	2.81512100	0.14873400
C	-7.04766300	3.01223400	1.03428000
C	-8.13698700	-2.80794300	1.04964900
C	-8.44902800	-4.22003900	-0.99673500
C	-7.88748100	-1.44499700	3.13084400
C	-7.75810000	0.95860000	-3.59550600
C	-7.74691200	3.44871800	-3.93937400
C	-7.82979000	-1.52686100	-3.29074700
C	-7.84019000	2.72888300	2.14832000
C	-8.10449000	1.76870100	4.45198500
C	-7.62886000	3.73526600	-0.13418300
H	-9.19516900	-2.89100200	1.26648200
O	-9.65185100	-4.49592300	-0.47857300
O	-8.11459700	-4.63336400	-2.10402200
O	-6.96532900	-1.23937100	4.08432000
O	-9.05595000	-1.10914100	3.22837300
H	-8.80272500	0.90831500	-3.88806000
O	-8.64647900	3.38058200	-4.93092200
O	-7.52502900	4.54260300	-3.43595600
O	-6.98728500	-2.56549200	-3.39081300
O	-9.04215900	-1.63065900	-3.21551400
H	-8.87180700	3.05952400	2.16589900
O	-9.23952600	2.47620300	4.49669800
O	-7.78502200	0.99996700	5.35494000
O	-6.67665900	4.26010800	-0.91665900
O	-8.82518100	3.82634700	-0.35382700
H	0.12479500	4.91026500	2.36649700
H	-0.39122100	-4.70013900	3.39397400
H	-0.44368900	-5.42674300	-1.84782100
H	-0.03236800	-0.94460900	-5.49569700
H	1.07146600	-2.25630400	-2.42087200
H	1.05539700	-1.48640500	3.05517400
H	1.29125400	2.95214300	-0.41173500
H	7.59300200	1.09076500	-2.74819000
H	9.26472500	-5.98805500	-1.30490100
H	8.17551700	-5.41770200	1.47148700
H	9.24782800	1.03340600	0.79863700
H	7.52182800	3.95480900	2.77268300
H	8.57561200	4.65363100	-4.38700100
H	-8.64656300	2.50504700	-5.35685000
H	-7.06711200	4.53662200	-1.78839700

H	-9.71583200	2.22370000	5.31174400
H	-7.32531100	-0.59452700	4.74403000
H	-10.15092700	-5.01016600	-1.14289400
H	-7.46705600	-3.40146200	-3.16226700
H	-0.18596500	0.71014200	5.58203900
O	-1.15446400	3.67283300	-1.60362300
H	-1.96791400	3.74215200	-2.13678400
H	-1.10349700	4.47580200	-1.05267000
C	1.81787200	2.40821800	-3.12890900
H	2.11152000	3.29767200	-2.55243200
H	2.60446300	2.22794300	-3.88174200
H	0.88676100	2.63573100	-3.66864800

Sum of electronic and thermal Free Energies = -6730.854837 (Hartree/Particle).

Supplementary Table 26. DFT-optimized geometry of PBA-MV^{2+} (singlet), computed at the B3LYP-D3/ 6-31G* (HCNO) level in water solvent using PCM.

Atom	x	y	z
C	-1.47210200	-0.60778000	2.00486300
C	-1.19846800	-1.35064700	0.85267900
C	0.15838500	-1.55194400	0.48267500
C	1.20124900	-0.93150700	1.24210900
C	0.88236800	-0.13310000	2.38371900
C	-0.46129500	-0.01216300	2.75932900
C	0.53917200	-2.36492500	-0.64168200
C	2.56781400	-1.09330200	0.85973000
C	2.90870500	-1.91988300	-0.25423100
C	1.84670800	-2.55041100	-0.98422500
C	4.25923700	-2.07107100	-0.61206800
C	5.26008600	-1.40932900	0.09913800
C	4.93754200	-0.58587300	1.17793600
C	3.60156500	-0.42098800	1.58346500
C	3.24079500	0.40332200	2.70473000
C	1.94210900	0.53000500	3.09401700
H	1.68084400	1.14507400	3.95110700
H	4.03162200	0.91769000	3.24390900
H	-0.23007200	-2.85700300	-1.22551100
H	-2.49878000	-0.46166300	2.32159900
H	-0.71887500	0.57501400	3.63682900
H	2.10405200	-3.17841500	-1.83299100
H	6.29895800	-1.53137000	-0.19287100
H	5.72294100	-0.06947800	1.72304000
C	-2.72271100	1.85438100	-1.10473900
C	-1.44043000	1.56507300	-1.52111600
C	-0.34572300	1.81312300	-0.67198400
C	-0.62121700	2.36254000	0.58735700
C	-1.92578200	2.63639400	0.95832000
N	-2.95312800	2.38407700	0.12202600
H	-3.59252100	1.67464800	-1.72332200
H	-1.31336000	1.16409500	-2.51800400
H	0.15637200	2.55414400	1.31337200
H	-2.17327600	3.04741400	1.92783800
C	1.04231100	1.52113900	-1.10026800
C	2.14063000	2.16205200	-0.50376500

C	1.31038800	0.60429000	-2.12913400
C	3.42354700	1.89034200	-0.93607700
H	2.02261500	2.88939700	0.28815900
C	2.61138900	0.37436600	-2.53123900
H	0.52768200	0.03468500	-2.61084600
N	3.64567800	1.01709800	-1.94530100
H	4.29299600	2.36807700	-0.50515900
H	2.85659400	-0.32359300	-3.32025600
C	-4.34972600	2.68593400	0.52038700
H	-4.96072700	1.79461400	0.36302000
H	-4.71276300	3.51153900	-0.09414300
H	-4.36187900	2.97073300	1.57057300
C	5.02395500	0.81427800	-2.44929200
H	5.72007500	0.91382800	-1.61864800
H	5.22829700	1.56695700	-3.21307100
H	5.10203900	-0.18640500	-2.86949100
H	4.51634000	-2.70566900	-1.45605700
C	-2.31174800	-1.91033000	-0.01587500
H	-2.30895300	-3.00839000	0.03676500
H	-2.08575900	-1.66731900	-1.06308400
C	-3.71756700	-1.38846800	0.29486600
H	-4.04634400	-1.71775500	1.28720400
H	-3.70646500	-0.29530900	0.31926400
C	-4.73859100	-1.83995900	-0.75097900
H	-4.90858200	-2.92059200	-0.71662900
H	-4.37117200	-1.61801800	-1.76344900
C	-6.06259200	-1.12227200	-0.61512200
O	-6.22475500	-0.02249200	-0.11719600
O	-7.07667700	-1.83032200	-1.14912000
H	-7.88832500	-1.29235600	-1.06683000

Sum of electronic and thermal Free Energies = -1496.792232 (Hartree/Particle).

From TDDFT calculation, λ_{abs} = 596.01 nm.

Supplementary Table 27. DFT-optimized geometry of PBA-MV$^+$ (doublet), computed at the UB3LYP-D3/ 6-31G* (HCNO) level in water solvent using PCM.

Atom	x	y	z
C	-1.31718400	-0.62505400	1.82233800
C	-1.12205600	-1.29591000	0.61185500
C	0.20735400	-1.53735700	0.17578500
C	1.30810400	-1.08976500	0.97115500
C	1.07250200	-0.39576100	2.19799900
C	-0.25042100	-0.17952100	2.60033900
C	0.50352100	-2.21736700	-1.05792500
C	2.65085300	-1.31958600	0.54260500
C	2.90544800	-2.00151500	-0.68563600
C	1.78510000	-2.43893600	-1.46649800
C	4.23429400	-2.21402600	-1.09321400
C	5.29717200	-1.77020100	-0.30859300
C	5.05966600	-1.09847700	0.89162100
C	3.74882600	-0.86080300	1.33596100
C	3.47366000	-0.16228800	2.56258000

C	2.19471300	0.05923700	2.97255400
H	1.99735600	0.59030000	3.90053100
H	4.31350500	0.18904400	3.15672100
H	-0.31294200	-2.56418900	-1.68121800
H	-2.32372000	-0.42365100	2.17116600
H	-0.44472900	0.35006700	3.52958100
H	1.97821800	-2.95347200	-2.40455200
H	6.31854800	-1.94172600	-0.63654300
H	5.89397500	-0.74978100	1.49498500
C	-2.77836200	1.77304800	-1.15329700
C	-1.45492000	1.57558100	-1.42257700
C	-0.43038200	2.01442800	-0.52187800
C	-0.91609500	2.65776900	0.66126300
C	-2.25183200	2.83006100	0.88819200
N	-3.19711300	2.39621700	-0.00403100
H	-3.56296200	1.45096100	-1.82674400
H	-1.21293300	1.07216500	-2.34947300
H	-0.23946900	3.01845200	1.42434900
H	-2.62768100	3.31063200	1.78256600
C	0.96265500	1.82815300	-0.78547800
C	1.98595600	2.28381400	0.10558900
C	1.44690800	1.16506000	-1.95778500
C	3.31030300	2.10860700	-0.17593600
H	1.74463100	2.76608600	1.04307600
C	2.78333700	1.01426300	-2.19403700
H	0.76977200	0.74032900	-2.68637400
N	3.72755600	1.48536000	-1.32159800
H	4.09179200	2.44183300	0.49454600
H	3.16089200	0.50781300	-3.07300400
C	-4.63209700	2.60670000	0.22749100
H	-5.15913400	1.64909500	0.20583100
H	-5.03741700	3.26937400	-0.54249600
H	-4.77030600	3.06822400	1.20520000
C	5.15423000	1.40205400	-1.65193800
H	5.73337900	1.36191500	-0.72936800
H	5.45639200	2.27216500	-2.24281200
H	5.33854800	0.48747100	-2.21592300
H	4.42446400	-2.73157100	-2.02998700
C	-2.29033900	-1.74684100	-0.24707500
H	-2.23910000	-2.83589400	-0.38842000
H	-2.17037600	-1.31419100	-1.25013700
C	-3.68413300	-1.37828100	0.26621400
H	-3.87115300	-1.84392300	1.24122000
H	-3.75236200	-0.29765800	0.41946400
C	-4.77459400	-1.81011900	-0.71562800
H	-4.80796000	-2.89866000	-0.83201200
H	-4.57266900	-1.40444800	-1.71759900
C	-6.14784200	-1.31746300	-0.32129300
O	-6.38057000	-0.38116400	0.42127500
O	-7.12641600	-2.02889700	-0.91859900
H	-7.97984000	-1.63432400	-0.65310100

Sum of electronic and thermal Free Energies = -1496.943707 (Hartree/Particle).

Supplementary Table 28. DFT-optimized geometry of BNAH (singlet), computed at the B3LYP-D3/ 6-31G* (HCNO) level in water solvent using PCM.

Atom	x	y	z
C	-4.23586100	-0.94791800	-1.06231600
C	-2.90345100	-1.18830800	-1.40769900
C	-1.87034900	-0.72242500	-0.59165900
C	-2.15728500	-0.00938300	0.57890500
C	-3.49448800	0.23531900	0.91450900
C	-4.52905900	-0.23440200	0.10275100
H	-5.03911600	-1.30868300	-1.69886800
H	-2.66645300	-1.73742200	-2.31506500
H	-0.83699200	-0.90650900	-0.87175400
H	-3.72770700	0.79867600	1.81544800
H	-5.56198700	-0.03518800	0.37538500
C	-1.04812100	0.47442000	1.50215900
H	-0.81735600	-0.29322500	2.24904400
H	-1.38789300	1.35593900	2.05816900
N	0.19754700	0.80609400	0.81804900
C	1.24649600	-0.07566500	0.77055600
C	0.23907900	1.93218000	-0.02209600
C	2.34007000	0.09126000	-0.01858600
H	1.13248600	-0.94149500	1.41480100
C	1.25764300	2.17310100	-0.85590300
H	-0.61778100	2.59117200	0.06732100
C	2.44595400	1.25118500	-0.99596800
H	1.22910800	3.07436300	-1.46111700
H	3.38529100	1.80279100	-0.83123100
C	3.45285800	-0.87147800	-0.03005600
O	4.29151200	-0.86426100	-0.94201600
N	3.51991400	-1.81896500	0.97723100
H	4.38779500	-2.34106900	0.99010000
H	3.17580700	-1.57617300	1.89706600
H	2.52955800	0.87369000	-2.02770900

Sum of electronic and thermal Free Energies = -688.360262 (Hartree/Particle).

Supplementary Table 29. DFT-optimized geometry of $BNAH^{\bullet+}$ (doublet), computed at the UB3LYP-D3/ 6-31G* (HCNO) level in water solvent using PCM.

Atom	x	y	z
C	-4.36807400	-0.67547500	-1.04171000
C	-3.23451600	-1.49019800	-1.13220900
C	-2.14524200	-1.26624000	-0.29200400
C	-2.18141600	-0.22390900	0.64479900
C	-3.31532900	0.59100600	0.73044500
C	-4.40829300	0.36284100	-0.10954200
H	-5.21695400	-0.85217500	-1.69594800
H	-3.20120900	-2.30041000	-1.85469000
H	-1.26540100	-1.90059500	-0.36359100
H	-3.34970600	1.39778900	1.45800300
H	-5.28722200	0.99626700	-0.03408400
C	-0.99074100	0.02264600	1.54446400
H	-0.67388600	-0.88549700	2.05904500
H	-1.20752300	0.78289200	2.29924400
N	0.18343600	0.50756500	0.76487900

C	1.32342400	-0.24719300	0.69108500
C	0.07942400	1.70741000	0.09717400
C	2.40530300	0.15528000	-0.04017900
H	1.29568200	-1.18687100	1.22773800
C	1.10233000	2.17797300	-0.66247400
H	-0.86228200	2.22786500	0.21368300
C	2.37371400	1.42807700	-0.81683400
H	0.98696000	3.12927400	-1.16902100
H	3.22840500	2.06895700	-0.53632100
C	3.62867600	-0.70174500	-0.19696300
O	4.26780300	-0.64927100	-1.24649100
N	3.93731400	-1.52321000	0.83763000
H	4.79341600	-2.05866600	0.76325400
H	3.58180500	-1.36574200	1.77094700
H	2.57467100	1.20776900	-1.88022200

Sum of electronic and thermal Free Energies = -688.181603 (Hartree/Particle).

Supplementary Table 30. DFT-optimized geometry of BNA$^{\bullet}$ (doublet), computed at the UB3LYP-D3/ 6-31G* (HCNO) level in water solvent using PCM.

Atom	x	y	z
C	-4.29624700	-0.89141000	-1.00505200
C	-3.01238200	-1.39677300	-1.23496300
C	-1.94565800	-0.99083800	-0.43305300
C	-2.14806800	-0.07408300	0.60736800
C	-3.43290400	0.43318600	0.82701100
C	-4.50446300	0.02401500	0.02826500
H	-5.12654500	-1.20665000	-1.63095800
H	-2.84271800	-2.10660500	-2.04004800
H	-0.94831400	-1.38048600	-0.62042500
H	-3.59698400	1.15286900	1.62572800
H	-5.49728500	0.42687800	0.20930200
C	-0.99217300	0.35144300	1.49825300
H	-0.72206100	-0.45554700	2.18684600
H	-1.29037800	1.20988900	2.11183800
N	0.21166700	0.71056000	0.75005500
C	1.31303200	-0.10454400	0.72716700
C	0.17512200	1.85224700	-0.08499400
C	2.40062000	0.15464000	-0.06761200
H	1.24899900	-0.98053400	1.36240000
C	1.24252800	2.12569300	-0.91027300
H	-0.72505300	2.44793200	-0.02373000
C	2.37559300	1.30308100	-0.94644900
H	1.18453500	3.00779200	-1.54223900
C	3.57703300	-0.74958800	-0.09408400
O	4.36619600	-0.75166900	-1.04564500
N	3.73764600	-1.61770700	0.96462900
H	4.61750300	-2.11873700	0.97181900
H	3.39391900	-1.35546300	1.87881300
H	3.21743000	1.49962700	-1.59646200

Sum of electronic and thermal Free Energies = -687.750847 (Hartree/Particle).

Supplementary Table 31. DFT-optimized geometry of CO_2 (singlet), computed at the B3LYP-D3/ 6-31G* (CO) level in water solvent using PCM.

Atom	x	y	z
C	0.00000000	0.00000000	0.00000000
O	0.00000000	0.00000000	1.16909400
O	0.00000000	0.00000000	-1.16909400

Sum of electronic and thermal Free Energies = -188.592753 (Hartree/Particle).

Supplementary Table 32. DFT-optimized geometry of H_2O (singlet), computed at the B3LYP-D3/ 6-31G* (HO) level in water solvent using PCM.

Atom	x	y	z
O	0.00000000	0.00000000	0.12073800
H	0.00000000	0.75883000	-0.48295200
H	0.00000000	-0.75883000	-0.48295200

Sum of electronic and thermal Free Energies = -76.412854 (Hartree/Particle).

Supplementary Table 33. DFT-optimized geometry of H_3O^+ (singlet), computed at the B3LYP-D3/ 6-31G* (HO) level in water solvent using PCM.

Atom	x	y	z
O	0.00000000	0.00000000	0.09006100
H	0.00000000	0.92910700	-0.24016200
H	0.80463000	-0.46455400	-0.24016200
H	-0.80463000	-0.46455400	-0.24016200

Sum of electronic and thermal Free Energies = -76.796658 (Hartree/Particle).

Supplementary Table 34. DFT-optimized geometry of CO (singlet), computed at the B3LYP-D3/ 6-31G* (CO) level in water solvent using PCM.

Atom	x	y	z
C	0.00000000	0.00000000	-0.65018700
O	0.00000000	0.00000000	0.48764000

Sum of electronic and thermal Free Energies = -113.324279 (Hartree/Particle).

Supplementary Table 35. DFT-optimized geometry of CH_3OH (singlet), computed at the B3LYP-D3/ 6-31G* (HCO) level in water solvent using PCM.

Atom	x	y	z
C	-0.04746600	0.66424300	0.00000000
H	-1.09412400	0.98284200	0.00000000
H	0.44008600	1.08452700	0.89234700
H	0.44008600	1.08452700	-0.89234700
O	-0.04746600	-0.76102800	0.00000000
H	0.87847600	-1.04912700	0.00000000

Sum of electronic and thermal Free Energies = -115.691949 (Hartree/Particle).

Supplementary Table 36. DFT-optimized geometry of CH_4 (singlet), computed at the B3LYP-D3/ 6-31G* (HC) level in water solvent using PCM.

Atom	x	y	z
C	0.00000000	0.00000000	0.00000000
H	0.63154200	0.63154200	0.63154200
H	-0.63154200	-0.63154200	0.63154200
H	-0.63154200	0.63154200	-0.63154200
H	0.63154200	-0.63154200	-0.63154200

Sum of electronic and thermal Free Energies = -40.491054 (Hartree/Particle).

References:

1 Peng, Y. et al. A versatile MOF-based trap for heavy metal ion capture and dispersion. *Nat. Commun.* **9**, 187 (2018).

2 Furukawa, H. et al. Water Adsorption in Porous Metal–Organic Frameworks and Related Materials. *J. Am. Chem. Soc.* **136**, 4369-4381 (2014).

3 Kuehnel, M. F. et al. Selective Photocatalytic CO_2 Reduction in Water through Anchoring of a Molecular Ni Catalyst on CdS Nanocrystals. *J. Am. Chem. Soc.* **139**, 7217-7223 (2017).

4 Barman, S. et al. Metal-Free Catalysis: A Redox-Active Donor–Acceptor Conjugated Microporous Polymer for Selective Visible-Light-Driven CO_2 Reduction to CH_4. *J. Am. Chem. Soc.* **143**, 16284-16292 (2021).

5 Singh, A. et al. Unraveling the Effect on Luminescent Properties by Postsynthetic Covalent and Noncovalent Grafting of gfp Chromophore Analogues in Nanoscale MOF-808. *Inorg. Chem.* **59**, 8251-8258 (2020).

6 Karmakar, S. et al. Covalent grafting of molecular photosensitizer and catalyst on MOF-808: effect of pore confinement toward visible light-driven CO_2 reduction in water. *Energy Environ. Sci.* **14**, 2429-2440 (2021).
7 in *Infrared and Raman Spectra of Inorganic and Coordination Compounds* 149-354 (2008).
8 Zych, D. et al. Pyrene derivatives with two types of substituents at positions 1, 3, 6, and 8 – fad or necessity? *RSC Advances* **9**, 24015-24024 (2019).
9 Li, X. et al. Selective visible-light-driven photocatalytic CO_2 reduction to CH_4 mediated by atomically thin $CuIn_5S_8$ layers. *Nat. Energy* **4**, 690-699 (2019).
10 Li, J. et al. Self-adaptive dual-metal-site pairs in metal-organic frameworks for selective CO_2 photoreduction to CH_4. *Nat. Catal.* **4**, 719-729 (2021).
11 Sorcar, S. et al. Highly enhanced and stable activity of defect-induced titania nanoparticles for solar light-driven CO_2 reduction into CH_4. *Materials Today* **Mater. Today20**, 507-515 (2017).
12 Zhang, H. et al. Efficient Visible-Light-Driven Carbon Dioxide Reduction by a Single-Atom Implanted Metal–Organic Framework. *Angew. Chem., Int. Ed.* **55**, 14310-14314 (2016).
13 Li, R. et al. Integration of an Inorganic Semiconductor with a Metal–Organic Framework: A Platform for Enhanced Gaseous Photocatalytic Reactions. *Adv. Mater.* **26**, 4783-4788 (2014).
14 Wang, M. et al. Self-assembly of CPO-27-Mg/TiO_2 nanocomposite with enhanced performance for photocatalytic CO_2 reduction. *Appl. Catal. B* **183**, 47-52 (2016).
15 Neaţu, Ş. et al. Gold–Copper Nanoalloys Supported on TiO_2 as Photocatalysts for CO_2 Reduction by Water. *J. Am. Chem. Soc.* **136**, 15969-15976 (2014).
16 Jin, J. et al. A Hierarchical Z-Scheme CdS–WO_3 Photocatalyst with Enhanced CO_2 Reduction Activity. *Small* **11**, 5262-5271 (2015).
17 Sadeghi, N. et al. A porphyrin-based metal organic framework for high rate photoreduction of CO_2 to CH_4 in gas phase. *J. CO2 Util.* **16**, 450-457 (2016).
18 Wang, S.-Q. et al. Cu_2O@Cu@UiO-66-NH_2 Ternary Nanocubes for Photocatalytic CO_2 Reduction. *ACS Appl. Nano Mater.* **3**, 10437-10445 (2020).
19 Huo, Y. et al. Amine-Modified S-Scheme Porous g-C_3N_4/CdSe–Diethylenetriamine Composite with Enhanced Photocatalytic CO_2 Reduction Activity. *ACS Appl. Energy Mater.* **4**, 956-968 (2021).
20 Guo, F. et al. Size Engineering of Metal–Organic Framework MIL-101(Cr)–Ag Hybrids for Photocatalytic CO_2 Reduction. *ACS Catal.* **9**, 8464-8470 (2019).
21 Qin, J.-S. et al. Creating Well-Defined Hexabenzocoronene in Zirconium Metal–Organic Framework by Postsynthetic Annulation. *J. Am. Chem. Soc.* **141**, 2054-2060 (2019).
22 Xu, H.-Q. et al. Visible-Light Photoreduction of CO_2 in a Metal–Organic Framework: Boosting Electron–Hole Separation via Electron Trap States. *J. Am. Chem. Soc.* **137**, 13440-13443 (2015).
23 Zeng, L. et al. Photoactivation of Cu Centers in Metal–Organic Frameworks for Selective CO_2 Conversion to Ethanol. *J. Am. Chem. Soc.* **142**, 75-79 (2020).
24 Yan, Z.-H. et al. Photo-generated dinuclear $\{Eu(II)\}_2$ active sites for selective CO_2 reduction in a photosensitizing metal-organic framework. *Nat. Commun.* **9**, 3353 (2018).
25 Fu, Y. et al. An Amine-Functionalized Titanium Metal–Organic Framework Photocatalyst with Visible-Light-Induced Activity for CO_2 Reduction. *Angew. Chem. Int. Ed.* **51**, 3364-3367 (2012).

26 Sun, D. et al. Construction of a supported Ru complex on bifunctional MOF-253 for photocatalytic CO_2 reduction under visible light. *Chem. Commun.* **51**, 2645-2648 (2015).

27 Wu, L.-Y. et al. Encapsulating Perovskite Quantum Dots in Iron-Based Metal–Organic Frameworks (MOFs) for Efficient Photocatalytic CO_2 Reduction. *Angew. Chem. Int. Ed.* **58**, 9491-9495 (2019).

28 Gao, W.-Y. et al. A Mixed-Metal Porphyrinic Framework Promoting Gas-Phase CO_2 Photoreduction without Organic Sacrificial Agents. *ChemSusChem* **13**, 6273-6277 (2020).

29 Kong, Z.-C. et al. Core@Shell $CsPbBr_3$@Zeolitic Imidazolate Framework Nanocomposite for Efficient Photocatalytic CO_2 Reduction. *ACS Energy Lett.* **3**, 2656-2662 (2018).

30 Verma, P. et al. Charge-transfer regulated visible light driven photocatalytic H2 production and CO2 reduction in tetrathiafulvalene based coordination polymer gel. *Nat. Commun.* **12**, 7313 (2021).

31 Jeevan, A. K. et al. Photoinduced Electron Transfer in a Self-Assembled Bis(β-cyclodextrin)-Linked Pyrene/Bis(adamantane)-Linked Methyl Viologen Donor–Acceptor System in Aqueous Solution. *J. Phys. Chem. B* **125**, 4428-4437 (2021).

32 Miyasaka, H. et al. Picosecond Absorption Spectra and Relaxation Processes of the Excited Singlet State of Pyrene in Solution. *Laser Chem.* **1**, 950145 (1983).

33 Brown, K. E. et al. Direct Observation of Ultrafast Excimer Formation in Covalent Perylenediimide Dimers Using Near-Infrared Transient Absorption Spectroscopy. *J. Phys. Chem. Lett.* **5**, 2588-2593 (2014).

34 Jiao, X. et al. Partially Oxidized SnS_2 Atomic Layers Achieving Efficient Visible-Light-Driven CO_2 Reduction. *J. Am. Chem. Soc.* **139**, 18044-18051 (2017).

35 Zhang, R. et al. Photocatalytic Oxidative Dehydrogenation of Ethane Using CO2 as a Soft Oxidant over Pd/TiO2 Catalysts to C2H4 and Syngas. *ACS Catal.* **8**, 9280-9286 (2018).

36 Sun, S. et al. Ultrathin WO3·0.33H2O Nanotubes for CO_2 Photoreduction to Acetate with High Selectivity. *J. Am. Chem. Soc.* **140**, 6474-6482 (2018).

37 Wang, W. et al. Hybrid carbon@TiO_2 hollow spheres with enhanced photocatalytic CO_2 reduction activity. *J. Mater. Chem. A* **5**, 5020-5029 (2017).

38 Liu, L. et al. Photocatalytic CO_2 Reduction with H_2O on TiO_2 Nanocrystals: Comparison of Anatase, Rutile, and Brookite Polymorphs and Exploration of Surface Chemistry. *ACS Catal.* **2**, 1817-1828 (2012).

39 Tan, T. H. et al. Unlocking the potential of the formate pathway in the photo-assisted Sabatier reaction. *Nat. Catal.* **3**, 1034-1043 (2020).

40 Schädle, T. et al. Monitoring dissolved carbon dioxide and methane in brine environments at high pressure using IR-ATR spectroscopy. *Anal. Methods* **8**, 756-762 (2016).
